# Data fusion and integrated species distribution models for three endangered ferns (*Culcita macrocarpa, Diplazium caudatum, and Pteris incompleta*) in a Mediterranean biodiversity hotspot

**DOI:** 10.3389/fpls.2025.1650159

**Published:** 2025-12-02

**Authors:** Ángel Ruiz-Valero, Jaime Francisco Pereña-Ortiz, Ángel Enrique Salvo-Tierra

**Affiliations:** Departament of Botany and Plant Physiology, Faculty of Sciences, University of Málaga, Málaga, Spain

**Keywords:** endangered ferns, plant biogeography, spatial ecology, integrated species distribution model, bayesian hierarchical model, state-space model, data fusion

## Abstract

**Introduction:**

Accurately modeling the distribution and abundance of rare and threatened species is considered critical for informing conservation strategies under increasing environmental pressures. Three threatened paleomediterranean relict ferns, *Culcita macrocarpa, Diplazium caudatum*, and *Pteris incompleta*, are restricted to climatically stable microhabitats within Los Alcornocales Natural Park (southern Spain), rendering them particularly vulnerable to environmental change.

**Methods:**

A joint-likelihood framework was employed within Integrated Species Distribution Models (ISDMs) to estimate spatiotemporal abundance of the three fern species. Structured abundance data (2014–2023) from the Andalusian Fern Recovery Plan were integrated with opportunistic presence-only records obtained from Global Biodiversity Information Facility (GBIF). Twenty-two model configurations were tested to evaluate the benefits of multi-species modeling and data-fusion strategies.

**Results:**

Predictive performance was improved by multi-species modeling, with shared ecological and spatial structures being captured more effectively. Spatiotemporal random effects were found to be more influential than fixed effects, reflecting local-scale heterogeneity in fern distributions. Spatiotemporal patterns were captured most effectively by the model excluding GBIF data fusion. Signs of overfitting were observed in the model incorporating data fusion, with GBIF inclusion failing to consistently improve predictive performance due to limited observations and spatial biases. Population trends were indicated to be generally stable, with localized increases and limited declines documented in two *C. macrocarpa* populations.

**Discussion:**

The value of ISDMs in leveraging complementary data sources is demonstrated by these findings, providing an effective framework for conservation planning in data-limited systems facing environmental change.

## 1 Introduction

Baseline knowledge and ongoing monitoring of plant diversity are essential for the planning and sustainable management of natural resources, as well as for the effective implementation of conservation, utilization, mitigation, and restoration strategies (Sutherland et al., 2004; Lindenmayer and Likens, 2010; Magurran and McGill, 2010; Pereira et al., 2013). Pteridophytes, owing to their ancient evolutionary origin, exhibit distribution patterns shaped by major geological and climatic events. Currently, around 13,000 species are recognized, most of which are concentrated in the intertropical belt, while species occurring in extratropical regions typically exhibit restricted ranges (Tryon & Tryon, 2012; Given, 1993). These relict taxa display highly localized and relatively stable distributions, shaped by strong selective pressures resulting from the interaction between historical environmental events and limited adaptive capacity (Sermolli, 1979). As a result, their persistence is often confined to microhabitats characterized by topoclimatic conditions that partially replicate the bioclimatic environments of their ancestral ranges (Rodríguez-Sánchez, 2011).

Effective conservation of these species demands a thorough understanding of the processes and factors that have shaped their current patterns of diversity. This entails examining not only the distribution and diversification of populations but also the environmental, spatial, and historical covariates influences (Luck, 2007; Keck et al., 2025). In a context where climate change and human activities pose increasing threats to species viability and habitat integrity, it is crucial to identify the determinants of their geographic distribution and anticipate potential shifts in their range (Weiskopf et al., 2020). These information needs have driven extensive species distribution data collection, fostering the development of multiple analytical techniques for modeling and interpretation (Guisan et al., 2002; Drake et al., 2006; Pearce and Boyce, 2006; Elith et al., 2006; Aarts et al., 2012; Renner et al., 2015; Adde et al., 2021; Doser et al., 2024). Increasingly, research has focused on model-based procedures to estimate abundance, density, or presence–absence from population sampling data. These approaches make explicit assumptions about spatial variation in populations, both in relation to explanatory variables and their intrinsic spatial structure. This analytical framework, aimed at producing spatially explicit inferences, falls under the concept of Species Distribution Models (SDMs) (Elith and Leathwick, 2009).

Most SDMs have traditionally been developed as purely spatial models focused on estimating species’ geographic distributions. However, there has been growing interest in spatiotemporal modeling approaches, where spatial variation is explicitly modeled as a function of time (Fidino et al., 2022; Johnston et al., 2023; Seaton et al., 2024). This framework not only allows for estimating the influence of environmental covariates on species distributions but also quantifies spatially explicit variability and trends in population parameters over time. Such approaches are particularly relevant in the context of biodiversity monitoring programs and recovery plans for threatened species (Bled et al., 2013; Pocock et al., 2015; Meehan et al., 2019). Analyzing spatiotemporal changes in species abundance helps identify areas undergoing significant transformations, regions especially vulnerable to environmental pressures, and locations where specific factors disproportionately influence ecological dynamics (Ward et al., 2015). This information is essential for assigning conservation status (Doser et al., 2024), generating hypotheses about drivers of population change (Crossley et al., 2021), and delineating priority areas for conservation or restoration, including potential climate refugia (Ethier et al., 2017). In this context, Spatially Varying Coefficient (SVC) models within a hierarchical Bayesian framework offer a powerful tool to estimate log-intensity trends that vary across space while explicitly propagating full uncertainty. Their application has become increasingly widespread in ecological research due to their potential to address key conservation and management questions (Thorson et al., 2023). Furthermore, by capturing local variability in population trends, SVC models improve predictive accuracy of species distribution changes, particularly in the face of emerging threats such as biological invasions (Thorson et al., 2023) and projected impacts of climate change and land-use alteration (Gonthier et al., 2014; Barnett et al., 2020).

Despite the inferential advantages of spatiotemporal models over purely spatial ones, their application remains limited by several factors. (1) The greater amount of data required to effectively estimate parameters and achieve robust inference and predictive performance (Bakka et al., 2018). (2) The widespread lack of consistent species observations across both time and space, which increases uncertainty and compromises model accuracy (Koshkina et al., 2017; Peel et al., 2019; Moonlight et al., 2020). These limitations are particularly pronounced when working with threatened or rare species, for which data scarcity is even more severe (Sofaer et al., 2019; Zhang et al., 2020; Erickson and Smith, 2023; Mondanaro et al., 2023; Zurell et al., 2023). Given the predictive challenges, several authors have emphasized the indispensable need to incorporate uncertainty estimates in SDMs applied to rare species, as data limitations lead to high uncertainty levels and potential biases that must be carefully accounted for in conservation decision-making.

Integrated Species Distribution Models represent an emerging extension of traditional SDMs, offering the ability to effectively increase the number of informative observations by integrating multiple data sources (Fletcher et al., 2019; Miller et al., 2019; Isaac et al., 2020). Several studies have proposed alternative frameworks for constructing ISDMs based on the combination of heterogeneous data types within a unified analytical framework. Fletcher et al. (2019) summarized a broad spectrum of integrated modeling techniques, ranging from simple data pooling to more robust approaches such as ensemble modeling and frameworks based on joint likelihood and shared components. Joint-likelihood models (hereafter referred to as ISDMs) are characterized by their capacity to integrate different types of ecological data (e.g., presence, abundance, density, biomass) as well as data collected at varying spatial and temporal scales (Isaac et al., 2020). These models are structured around sub-models that link an unobserved latent state, representing the true distribution of a species, with one or more observation models that describe how the observed data were generated from this latent state. While observation models are specific to each data set, the latent state and its defining parameters are shared across all data sources through a joint likelihood formulation (Pacifici et al., 2017). By jointly modeling the different data sources and their respective observation processes, ISDMs allow inference of the latent species distribution (Doser et al., 2025). Numerous studies have demonstrated that joint models provide an efficient approach to data integration, improving estimation and accounting for collection biases (Fithian et al., 2015; Koshkina et al., 2017; Peel et al., 2019). Similarly, Pacifici et al. (2017) found that integrated models consistently outperformed single-source models in predictive accuracy, as long as the underlying assumption of relatedness between data sources was met.

The growing interest in ISDMs stems from their dual capability to simultaneously integrate multiple species and fuse different types of ecological data within a single modeling framework. (1) As conservation and management challenges increasingly shift toward a community-level perspective, single-species models have evolved into multi-species frameworks. These models are based on the premise that species coexisting within the same communities and/or sharing similar ecological niches are likely to respond similarly to environmental covariates and exhibit comparable spatiotemporal patterns (Erickson and Smith, 2023). This assumption is particularly relevant for ferns, where species with similar distributional patterns have been shown to share comparable hydrological and bioclimatic requirements (Sermolli et al., 1988; Márquez et al., 1997). Joint modeling of ecologically similar species enables statistical information to be shared, thereby increasing the models’ predictive power and precision (Erickson and Smith, 2023; Clark et al., 2025), improving robustness to biases, compensating for data deficiencies in rare species (Ahmad Suhaimi et al., 2021; Mäkinen et al., 2024), and facilitating the detection of ecological relationships that would otherwise go unnoticed in single-species models, often due to data scarcity (Jung, 2023). (2) Scientific species observations typically originate from standardized sampling protocols designed for specific research goals, offering high-quality data but often with limited spatial and temporal coverage due to cost constraints. In contrast, the rapid growth of citizen science has produced a complementary data stream for SDMs (August et al., 2015; Bayraktarov et al., 2019), albeit with less methodological control, as sampling locations are not randomly selected and often reflect preferential sampling biases (Ver Hoef et al., 2021). Traditionally, these data were discarded when scientific observations were available, leading to the loss of potentially valuable information. ISDMs provide a solution by enabling the integration of both structured and opportunistic data, leveraging the strengths of each and enhancing species distribution estimation and prediction (Dambly et al., 2023).

Current anthropogenic climate change (Oreskes, 2004) poses a major threat to sensitive ecosystems such as the Alboran Arc, which serves as a refugium for multiple relict fern species from the Paleomediterranean flora. Increasing rates of population extinction have been directly linked to the effects of climate change (Ovaskainen and Meerson, 2010), with the most vulnerable species typically being stenoecious taxa restricted to exceptional topoclimatic conditions (Guisan and Thuiller, 2005; RSCG, 2017). This study evaluates the capacity of ISDMs to characterize the spatial distribution and temporal abundance dynamics of three threatened fern species in the southernmost region of the Alcornocales Natural Park, Spain, *Culcita macrocarpa* C. Presl, *Diplazium caudatum* (Cav.) Jermy, and *Pteris incompleta* Cav. All three taxa, considered Paleomediterranean relicts, are legally protected and currently classified as endangered. The three species share a narrow and highly specialized ecological niche, characterized by pronounced sciophily and hygrophily, with common requirements for high atmospheric and edaphic humidity, mild temperatures, and low thermal variability. They are typically confined to specific microhabitats, locally referred to as *canutos*, such as deeply incised valleys, shaded ravines, and north-facing riparian zones. These environments are defined by persistent fog retention, perennial watercourses, and dense canopy cover, which together maintain stable and humid microclimatic conditions throughout the year (Salvo-Tierra, 1990).

In this study, 22 alternative ISDM structures were evaluated, integrating data from citizen science records (via the Global Biodiversity Information Facility, GBIF) and annual abundance monitoring (2014–2023) collected under the Andalusian Fern Recovery Plan (Junta de Andalucía, 2015). The main objective of this study is to evaluate the added value of simultaneously modeling multiple species within a spatiotemporal framework, using shared components that enable multi-species information exchange, and to assess the effectiveness of data-fusion strategies that combine presence-only and abundance datasets. Given the ecological similarity, frequent co-occurrence in sites with analogous microclimates, and consistent association within the same plant communities of the studied taxa, the application of ISDMs appears particularly appropriate. These models can capitalize on the spatial and ecological complementarity among species to improve the inference of spatiotemporal distribution patterns. It is hypothesized that incorporating shared modeling across species will enhance both model robustness and predictive performance, reflecting the ecological cohesion in their habitat preferences. This improvement may be especially relevant considering the limited number of observations for all three species, where information sharing could strengthen parameter estimation in contrast to independent modeling. However, data fusion is not necessarily expected to yield substantial gains in predictive accuracy, as when two data sources exhibit high spatial concordance, the informational content may be redundant, leading to minimal benefit from integration (Dovers et al., 2024). The restricted distribution of these species also limits the availability of GBIF records, whose spatial pattern, closely aligned with that of the structured abundance data, may result in the lack of improvement observed. Moreover, spatial heterogeneity in temporal abundance trends is anticipated, likely driven by local-scale factors that are not directly representable as spatial covariates and therefore cannot be incorporated into ISDMs, such as site-specific reductions in water availability for spore germination and fertilization, pressure from herbivores or livestock, microhabitat loss due to drought or landslides, anthropogenic disturbance associated with recreational use and even human sampling mistakes. Consequently, model estimates are expected to be less reliable in the northern sector of the park, due primarily to the restricted distribution of the studied species, which are largely confined to the central-southern portion.

## 2 Materials and methods

### 2.1 Study area

**Figure 1:**
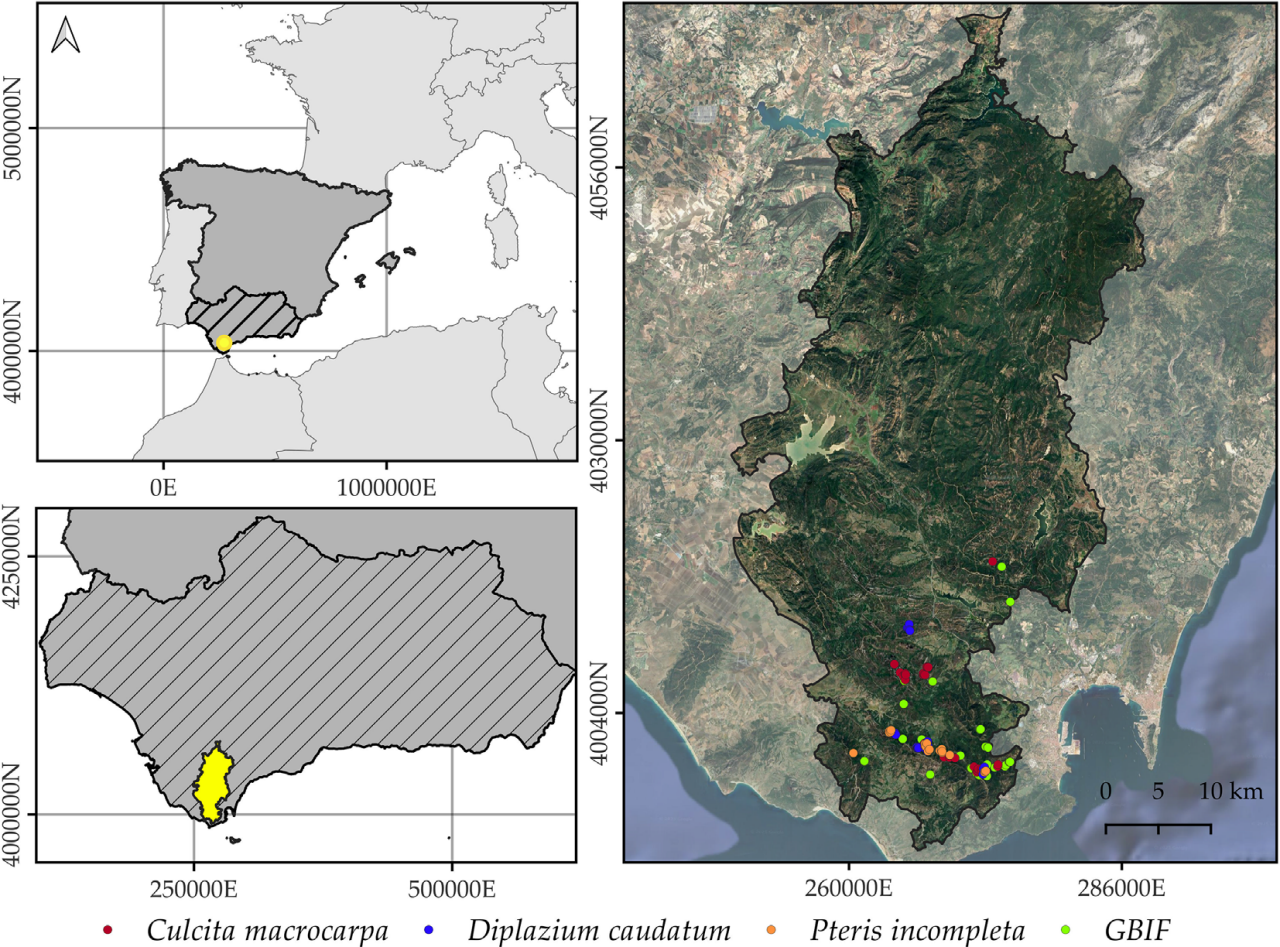
Geographic location of Alcornocales Natural Park. The map shows the spatial distribution of *Culcita macrocarpa*, *Diplazium caudatum*, and *Pteris incompleta* populations that are annually monitored as part of the Andalusian Fern Recovery Plan (Junta de Andalucía, 2015). GBIF-derived occurrences are not species-differentiated in this figure. The orthophotography background corresponds to the 2022 National Plan for Aerial Orthophotography (NIG, 2022). Coordinate Reference System: EPSG 25830.

The Alcornocales Natural Park (**Figure 1**), covering approximately 174,000 hectares, is a protected area located in the southwestern Iberian Peninsula, officially designated in 1989, by the Law 2/1989, of 18 July, approving the Inventory of Protected Natural Areas of Andalusia and establishing additional measures for their protection. It spans the provinces of Cádiz and Málaga in Andalusia, southern Spain, and harbors the southernmost cork oak (*Quercus suber* L.) forest in Europe (Pérez Latorre et al., 1999). Biogeographically, the study area lies within the Aljíbic Sector of the coastal Lusitanian-Andalusian Province, part of the Mediterranean Region (Rivas Martínez, 2007). According to Rivas- Martínez et al. (2001), the park’s climax vegetation generally corresponds to a climax cork oak (*Quercus suber* L.) woodland with wild olive (*Olea sylvestris* Mill.) (*Oleo sylvestris–Quercetum suberis*) in the lower elevations, transitioning to Andalusian gall oak forests (*Quercus canariensis* Wild) (*Rusco hypophylli–Quercetum canariensis*) in more humid and elevated zones.

The region is characterized by a relatively humid Mediterranean climate with strong oceanic influence and falls bioclimatically within the thermo- and mesomediterranean belts (Rivas-Martínez et al., 2011). Temperatures typically range from 7°C to 27°C, with a mean annual temperature of approximately 15.7°C and moderate seasonal variability. Annual precipitation averages 1,065 mm but may reach up to 1,300 mm in some areas, with pronounced seasonality (Chazarra-Bernabé et al., 2018). These climatic conditions, high rainfall, elevated relative humidity, and the convergence of moist Atlantic and Mediterranean winds, foster the formation of a persistent “fog belt” in the southern sector of the park, particularly in the Strait of Gibraltar region, which can last for over 200 days per year (Díez Garretas and Salvo Tierra, 1980).

These unique environmental and geographic features make the park an important biogeographic refuge within the Mediterranean Basin, distinguished by high floristic richness and the presence of numerous relict taxa, including the three focal fern species of this study (Rodríguez-Sánchez, 2011). *C. macrocarpa*, *D. caudatum*, and *P. incompleta* thrive in edaphohygrophilous plant communities associated with very humid conditions, frequently linked to phytosociological associations such as *Rusco hypophylli–Quercetum canariensis*, *Rhododendro pontici–Prunion lusitanicae*, *Frangulo baeticae–Rhododendretum pontici*, and *Scrophulario laxiflorae–Rhododendretum baetici* (Díez Garretas and Salvo Tierra, 1980; Pérez Latorre et al., 1999, 2000).

### 2.2 Data description

#### 2.2.1 Structured abundance data

Abundance data for *C. macrocarpa*, *D. caudatum*, and *P. incompleta* were obtained from restricted-access records provided by the Andalusian Fern Recovery and Conservation Plan. This plan systematically delineated known populations of the target species within Alcornocales Natural Park. Since 2014, the number of individuals in each population has been recorded, with sampling areas kept constant over time, an aspect that lends these data high value for rigorous population trend analysis. In total, 50 distinct populations are included: 22 for *C. macrocarpa*, 17 for *D. caudatum*, and 11 for *P. incompleta*. Data gaps vary substantially across populations, ranging from a minimum of 7.14% to a maximum of 81.8%, with a median missing rate of 27.2%. The first and third quartiles are 16.8% and 42.1%, respectively. At the species level, missing data account for 18.4% of *P. incompleta* records, 26.2% for *C. macrocarpa*, and 21.1% for *D. caudatum*. Sampled population areas range from 0.11 to 3.81 hectares, with a median of 0.68 hectares. Notably, the monitored areas for *C. macrocarpa* and *D. caudatum* are generally smaller than those for *P. incompleta*, with observed ranges of [0.24, 1.15], [0.34, 1.26], and [0.65, 2.6] ha, respectively. Finally, after accounting for years with missing data (i.e., years in which annual censuses were not conducted) for each population specifically, the structured abundance dataset contains a total of 1,037 observations available for modeling, distributed across populations and years.

#### 2.2.2 Citizen data: opportunistic occurrence data

Species occurrence data were retrieved from the Global Biodiversity Information Facility (GBIF) using the rgbif package v.3.8.1 (Chamberlain and Boettiger, 2017; Chamberlain et al., 2024). A series of quality filters were applied to ensure the reliability of the occurrence records. Only records with valid geographic coordinates and no reported geospatial issues were retained. Fossil and cultivated specimens, as well as records lacking a confirmed presence status, were excluded. Observations within 2 km of known administrative centroids (following standard GBIF filtering protocols to remove records with imprecise geographic coordinates assigned to country or provincial centers) were examined, though no such centroids existed within our study area, resulting in no data loss from this filtering step. Observations within institutions such as zoos or botanical gardens were removed. Observations with default or placeholder uncertainty values commonly linked to low coordinate reliability (e.g., 301, 3036, 999, or 9999 meters) were also discarded. Records with a spatial resolution lower than 0.01 degrees or a coordinate uncertainty greater than 1,000 meters were excluded, except in cases where these metadata were not reported. After data cleaning and filtering, a total of 47 georeferenced records were retained: 24 for *C. macrocarpa*, 13 for *D. caudatum*, and 10 for *P. incompleta.*

### 2.3 Covariables description

#### 2.3.1 Bioclimatic covariables

Based on monthly time series of maximum, minimum, and mean temperatures, as well as precipitation data for the period 2014–2019, obtained from CHELSA v.2.1 (Climatologies at High Resolution for the Earth’s Land Surface Areas) (Karger et al., 2017, 2018), we derived annual time series of bioclimatic variables. The selected variables included: annual mean temperature (BIO01), maximum temperature of the warmest month (BIO05), minimum temperature of the coldest month (BIO06), annual temperature range (BIO07), annual precipitation (BIO12), precipitation of the wettest quarter (BIO16), precipitation of the driest quarter (BIO17), precipitation of the warmest quarter (BIO18), precipitation of the and coldest quarter (BIO19). These variables were chosen from among the 19 commonly used bioclimatic predictors based on their ecological relevance, as they act as proxies for the main climatic requirements of the species: mild temperatures, low thermal variability, and continuous atmospheric and edaphic humidity throughout the year. Alignment was ensured between the CHELSA pixels and the 100 × 100 cells of the prediction grid, so that each cell within a CHELSA pixel inherits the corresponding value for the bioclimatic variables. To ensure temporal consistency with the abundance dataset, the annual time series were linearly extrapolated to obtain estimated values up to the year 2023.

#### 2.3.2 Topographic covariables

The Copernicus Digital Elevation Model (EEA, 2024) with a spatial resolution of 30 m (GLO-30) was employed. Considering the geomorphological characteristics of the areas where the target species typically occur, the following topographic variables were derived: elevation, slope, aspect, topographic solar radiation index (TSRI), topographic position index (TPI), topographic wetness index (TWI), profile curvature, plan curvature, and mean curvature. All variables were generated using the ‘WhiteboxTools’ library (Lindsay, 2016) via the R package whitebox v.2.20 (Wu and Brown, 2022). Additionally, based on hydrological maps at a 1:5000 scale (NIG, 2023), euclidean distance to watercourses was calculated. These topographic variables were selected primarily for their ability to capture the structure of *canutos*, deeply incised valleys, shaded ravines, and north-facing riparian zones; and secondly for their capacity to approximate water flow, water accumulation, and/or humidity. Consequently, they act as proxies for the hygrophilous trait of these species. Moreover, they represent the main alternative given the lack of variables with sufficient resolution to adequately capture these features.

#### 2.3.3 Forest canopy structure covariables

Airborne LiDAR data from the Second Coverage of the National Plan for Aerial Orthophotography (NIG, 2022), with a minimum point density of 1.5 points/m², were used to derive variables related to forest canopy structure, given the importance of closed riparian forests (“*canutos”*) in the distribution of the studied ferns (Salvo-Tierra, 1990). Point cloud processing was conducted using the lidR package v.4.1.2 (Roussel et al., 2020; Roussel and Auty, 2024). Canopy Height Models with a resolution of 2.5 m and a minimum height threshold of 3 m were generated to accurately identify trees and avoid inclusion of misclassified objects. Canopy height was estimated as the 95th percentile of return heights per pixel. Canopy cover was calculated as the percentage of returns above 3 m, while vertical structure was segmented into return percentages below 3 m, between 3–8 m, 8–15 m, and above 15 m. To avoid multicollinearity inherent in this type of compositional data (i.e., proportions summing to 1), an isometric log-ratio (ILR) transformation was applied using the compositions package v.2.0-8 (van den Boogaart et al., 2024).

Vertical stand heterogeneity has been addressed through the Height Variation Hypothesis, which proposes that increased vertical complexity in forest structure leads to a greater number of subhabitats and ecological niches, thereby enhancing species diversity (Torresani et al., 2020; Moudrý et al., 2023). The vertical distribution of canopy elements plays a key role in shaping the spatiotemporal dynamics of forest resources and is considered a major driver of ecosystem functions such as habitat diversification and environmental heterogeneity (Palmer et al., 2002). In this context, Ishii et al. (2004) demonstrated that structurally complex forests promote biodiversity by increasing environmental heterogeneity, including microhabitat variability and a broader range of microclimatic conditions, which directly influence understory plant diversity.

Vertical heterogeneity was quantified using Rao’s Q index (Rao, 1982), which simultaneously incorporates richness, relative abundance, and the magnitude of differences in height within each pixel. This index represents an improvement over the Shannon entropy index, which does not account for the magnitude of differences between categories (Rocchini et al., 2017). Rao’s Q is defined as the expected difference, calculated as the Euclidean distance, in height values between two pixels randomly selected with replacement from the evaluated set (Equation 1).

(1) $$Q=\;\sum_{i=1}^{N}\sum_{j=1}^{N}d_{ij}p_{i}p_{j}$$

where, given the height values of different pixels i and j, dij represents the euclidean distance between those heights, and pi and pj ​ denote the relative frequency (representativeness) of those values within the total set of pixels considered. The calculation was performed using only the integer height values of the returns.

Similarly, using Rao’s Q index on canopy cover, horizontal heterogeneity was calculated over a 100 x 100 meter grid. This metric quantifies the spatial diversity of canopy cover degrees, which is useful for distinguishing between different habitat types, identifying both structurally diverse habitats and ecological transition zones.

#### 2.3.4 Site accessibility covariates

Most citizen science data are collected without a formal sampling design, and therefore, according to Kelling et al. (2019), there is no “fully statistically defensible” way to correct for the inherent biases in such data collection. Statistical bias occurs when the expected value of a statistical technique differs from the true value of the quantity being estimated. Some authors (e.g., Fletcher et al., 2019; Adde et al., 2021; Seaton et al., 2024) have incorporated specific structures into models that allow for correction of biases inherent in the data by including particular effects for each dataset that may influence the observed preferential sampling. In the present study, the inclusion of distance to access roads and population centers was evaluated. Only the logarithm of the distance to access roads was incorporated into the model, as it was the only variable that showed a significant association with the spatial pattern of GBIF data.

#### 2.3.5 Covariables processing and selection

To avoid issues of correlation and collinearity among explanatory variables, the Pearson correlation coefficient and the generalized variance inflation factor (GVIF) were calculated beforehand using the car package in R (v.3.0-12) (Fox and Weisberg, 2019). Pairs of variables showing high correlation (Pearson’s r > 0.7) or elevated GVIF (GVIF > 5) were identified, retaining only one variable per pair in the final model. Between the two variables, the one with greater ecological relevance for the species was selected, and when both had equal influence, the one with the higher linear correlation coefficient with abundance was chosen. The final set of selected covariates included: annual mean temperature (BIO01), annual temperature range (BIO07), precipitation of the wettest quarter (BIO16), distance to rivers, Topographic Position Index (TPI), Rao’s Q vertical heterogeneity index, and intrinsic log-ratio (ILR) transformations of canopy cover percentages in height classes between 3 and 8 meters, between 8 and 15 meters, and above 15 meters, as well as Rao’s Q horizontal heterogeneity index (**Supplementary Figure S1**). After this selection process, none of the covariates exceeded the established thresholds, with maximum observed values of 0.61 for correlation and 2.48 for GVIF. To facilitate model fitting in R-INLA, all covariates were standardized using a z-score transformation.

### 2.4 Model description

The evaluated ISDMs were based on the approach described by Isaac et al. (2020), which formulates the model as a state-space point process model. State-space models are hierarchical models composed of two main submodels: the observation process and the latent state process. The latent state represents the true species distribution and is modeled as a function of environmental covariates and spatiotemporal effects. Meanwhile, the observation process statistically describes how the data were generated, conditional on the latent state. Within this conceptual framework, an ISDM is characterized by including multiple observation submodels that share a common latent state; each submodel represents a data type for a given species, enabling joint inference on their underlying distribution (Isaac et al., 2020).

For each species, there exists a latent state representing its “true” distribution, and two observation models: one for count data and another for presence data obtained from GBIF. The true distribution for each species j, denoted as λ_j_(s,t), is modeled as the intensity of a Poisson point process as a function of covariates X and parameters associated with random effects ϕ_j_, such that p(λ_j_(s,t)∣X,ϕ_j_). The observation models, for each species j, link the observed data in each of its k = 2 data types to the underlying state, conditioned on this latent state and a set of parameters specific to the observation model θ_jk_, such that Pr(Y_jk_ ∣ λ_j_(s,t), θ_jk_). Thus, the joint likelihood of the model is defined as the product of the conditional observation distributions for all species and data types, conditional on the corresponding latent distribution, which is summarized in Equation 2.

(2) $$L({\left\{Y_{count,j},Y_{press,j}\right\}}_{j=1}^{N_{spp}=3}|X,\{\phi_{j}\},\{\theta_{j}\},\{\gamma_{j}\})\propto \prod_{j=1}^{N_{sps}=3}[p(\lambda_{j}(s,t)|X,\phi_{j})\prod_{i=1}^{n_{count,j}}Pr(Y_{count,j,i}|\lambda_{j}(s_{i},t_{i}),\theta_{j})\cdot \prod_{k=1}^{n_{press,j}}Pr(Y_{press,j,k}|\lambda_{j}(s_{k},t_{k}),\gamma_{j})$$

#### 2.4.1 Latent state model

A point process is a statistical description of the continuous spatial distribution of points (Diggle, 2014; Baddeley et al., 2015), thus representing the instantaneous locations of individuals or their populations. This framework forms the basis for integrating multiple data sources, since the degradation of the point process naturally leads to other data types such as counts or abundances, corresponding to the number of points within a given region, or presence/absence data, which merely indicate whether points exist in a specified area (Isaac et al., 2020). The process describing point locations is characterized by an underlying intensity function, λ(s,t), representing the expected density of points in space, or equivalently, the expected number of individuals per unit area. Under the assumption of a log-Cox point process model, that is, a Poisson process (complete spatial randomness) with spatially varying intensity, where the logarithm of this intensity is modeled via a Gaussian linear predictor, a Log-Gaussian Cox Process (LGCP) is obtained (Møller et al., 1998). LGCPs, which are computationally intensive, belong to the class of Latent Gaussian Models (LGMs), a particular case of Bayesian Hierarchical Models (BHMs) characterized by an additive structure in the linear predictor and an observation process conditional solely on this predictor and parameters specific to the chosen likelihood. LGMs can be efficiently estimated using the Integrated Nested Laplace Approximations (INLA) method and the approximation of Gaussian Fields as Gaussian Markov Random Fields via the Stochastic Partial Differential Equations (SPDE) approach with a discrete Delaunay triangulation mesh (Rue et al., 2009; Lindgren et al., 2011; Illian et al., 2012; Simpson et al., 2016).

The latent distributions of the three species were therefore modeled as LGCPs with intensity λ_j_(s,t), which defines the density of individuals at location s and year t for species j (Equation 3).

(3) $$\begin{array}{c} \lambda_{j}(s,t)=\;e^{\eta_{j}(s,t)}\\ \eta_{j}(s,t)=\;\sum_{i}^{P}\beta_{i}X_{i}(s,t)+u(s,t)+\delta_{j}(s,t)+\omega_{j}(s)\cdot T(s,t) \end{array}$$

Where the set {X_i_(s,t)} denotes the predictors. The set {β_i_} the fixed effects of the covariates, which can be jointly estimated across all species and data types under a shared component modeling (SCM) framework (Knorr-Held and Best, 2001; Held et al., 2005; Simmonds et al., 2020). The term u(s,t) constitutes a separable spatio-temporal random effect, jointly estimated for all species as the Kronecker product between the precision matrix of the SPDE effect (Gaussian field approximation) and the precision matrix of the first-order autoregressive temporal structure. δ_j_(s,t) represents a species-specific separable spatio-temporal random effect, estimated exclusively from each species’ own count and presence data, allowing for the capture of species-specific deviations from the shared spatio-temporal pattern. The SPDE × AR(1) structure for spatio-temporal random effects was selected as it represents the most direct way to model spatial variability in the log intensity, while assuming a direct influence of the system’s state in the previous year. ω_j_(s) denotes the SVC effect for species j on the covariate T(s,t), which corresponds to the year. Under the SCM approach, it was assumed that the SVC effects of *D. caudatum* and *P. incompleta* correspond to a linear scaling of the effect estimated for *C. macrocarpa*.

#### 2.4.2 Observation models

##### 2.4.2.1 Observation model for structured abundance data

Abundances for *C. macrocarpa* and *P. incompleta* were modeled using a Poisson distribution. For *D. caudatum*, a Negative Binomial distribution was assumed due to observed overdispersion after accounting for fixed and random effects. The approximation proposed by Illian et al. (2012) was adopted, as the format of the count data (individual counts within polygonal spatial units) precluded the application of the method proposed by Simpson et al. (2016). Within the 100 × 100 m prediction grid, the number of individuals per cell was derived by proportional redistribution based on the area of intersection between populations and grid cells. This approach assumes a homogeneous distribution of individuals within each population, such that grid cells with a greater proportion of overlap with a population contain a correspondingly higher number of individuals.

The number of individuals of species *j* in cell *s* at time *t*, conditional on the underlying intensity λ_j_(s,t), is defined by the observational model (Equation 4). This is incorporated through a logarithmic link function that connects it to the linear predictor of the latent model.

(4) $$\begin{array}{c} Y_{j}^{count}(s,t)\;|\eta_{j}(s,t)∼Poisson(\mu_{j}(s,t)=|a_{s}|\cdot \lambda_{j}(s,t))\\ Y_{D.caudatum}^{count}(s,t)|\eta (s,t)∼NegBin(\mu (s,t)=|a_{s}|\cdot \lambda (s,t),\;\psi ) \end{array}$$

where ∣as∣ represents the sampled area within cell s. Thus, the logarithm of the intersection area between the polygon defining the population and the considered cell is incorporated into the model as an offset. On the other hand, Ψ represents the hyperparameter controlling overdispersion in the count data for *D. caudatum*.

##### 2.4.2.2 Observation model for opportunistic occurrence data

Given the limited number of occurrences and their restricted temporal coverage, two assumptions were adopted. (1) All observations after 1989, the year the study area was declared a Natural Park, were treated as timeless, assuming a single constant spatial pattern replicated throughout the temporal series. This approach is based both on the legal protection of the area and the species, and on the relict nature of the studied species, whose historical presence suggests spatial stability in their known locations. (2) The point pattern was simplified to species-specific presence-absence data, considering presence when the individual count in a grid cell is greater than zero. This approach also assumes that presence data represents potential distribution areas of the species. The integration of these data with the latent process model was performed through an observation model in which the probability that species j is present in cell s at time *t*, p_j_(s,t), is modeled using a Bernoulli distribution with a complementary log-log (cloglog) link function (Adde et al., 2021). This formulation allows expressing the presence probability as a direct function of the intensity λ_j_(s,t) of the underlying latent model (Equation 5).

(5) $$\begin{array}{c} Y_{j}^{pres}\sim \;Bernoulli(p_{j}(s,t))\\ \;p_{j}(s,t)=Pr(Y_{j}^{count}(s,t)>0)=1-Pr(Y_{j}^{count}(s,t)=0)=1-e^{-\lambda_{j}(s,t)}=1-e^{-e^{\eta_{j}(s,t)}}\\ cloglog(p_{j}(s,t))=\log(-\log(1-(1-p_{j}(s,t))))=\eta_{j}(s,t) \end{array}$$

where the term η_j_(s,t), the log-intensity, includes the shared components of the latent model, and species-specific intercepts are incorporated to capture differences in the observation processes among species. Additionally, the logarithm of the distance to access roads was included as an observational covariate due to the sampling bias present in the GBIF data, following approaches like those proposed in previous studies (e.g., Adde et al., 2021; Seaton et al., 2024).

#### 2.4.3 Mesh and prior distributions specification

The mesh was generated using the boundary of the Alcornocales Natural Park as the domain boundary, applying Delaunay triangulation. Due to the absence of data in the northern sector of the study area, a reduced study domain was defined using a non-convex hull, based on both the park boundaries and the spatial distribution of observations (**Figure 1**). The resulting meshes are shown in **Supplementary Figure S2**. The following parameters were used for its construction: (1) The maximum allowed edge length of the triangles within the domain was set to 1000 m, i.e., one fifth of the prior range of 5000 m, following general recommendations (e.g., Dambly et al., 2023). The prior range is defined as the distance at which spatial correlation approximately drops to 0.13. Given the lack of specific bibliographic information for this parameter, it was selected by measuring Euclidean distances between the centroids of the studied populations and choosing an average distance. (2) The triangulation was extended 2000 m beyond the study area boundary, with a maximum allowed edge length of 2000 m in this outer zone. This additional extension is solely intended to reduce boundary effects, and no predictions are generated within this space. (3) The minimum allowed distance between mesh nodes was set at 200 m, equivalent to one fifth of the maximum allowed edge length.

For the fixed effects in the model, Gaussian prior distributions with a mean of zero and a standard deviation of 1000 were used. In the case of the hyperparameters controlling overdispersion, ψ, a Penalized Complexity prior (PC) was applied to the gamma parameter (Simpson et al., 2017; Simpson, 2022), which progressively penalizes model complexity in favor of a Poisson distribution, i.e., toward the absence of overdispersion. For the spatio-temporal effects estimated via SPDE-AR, both the shared component u(s,t) and the species-specific components δ_j_(s,t), a joint prior distribution was specified using PC priors on the range and marginal standard deviation of the spatial field. These priors are weakly informative and penalize model complexity by shrinking the Matérn covariance function’s range parameter toward infinity and the marginal standard deviation toward zero (Fuglstad et al., 2018). It was assumed that the range has a 0.1 probability of being less than 5000 m, while the marginal standard deviation has a 0.5 probability of exceeding the value of 2. The autoregressive order 1 structure associated with these effects also included a PC prior, assuming a 0.9 probability that the temporal autocorrelation is greater than zero. For the SVC effect, ωj(s), a PC prior was also used, assuming a 0.5 probability that the spatial range is less than 3000 m, and a 0.5 probability that the marginal standard deviation is greater than 2. A lower range was chosen compared to the spatio-temporal effects to minimize potential confounding between them, and because annual trends in grid abundance are expected to display more localized patterns. The scaling parameter of the SVC across species was assigned a Gaussian prior distribution with a mean of zero and a standard deviation of 10.

### 2.5 Model selection and comparison

The final modeling dataset comprises 1,037 spatio-temporal observations from the structured abundance data (50 population locations × variable years of monitoring) and, when fused with GBIF occurrence data, an additional 470 observations (47 GBIF locations treated as i.i.d. presences in time across 10 years), yielding a combined dataset of up to 1,507 spatio-temporal observations depending on the analyses conducted.

A total of 22 different models were evaluated (**Table 1**), varying in terms of their structural complexity. The differences among models are based on the inclusion of different types of random effects: spatial, spatio-temporal, and Spatially Varying Coefficients (SVCs). As well as on the approach adopted for estimating fixed and random effects, whether shared across species and/or data types, or independently for each species. The models also differ in whether or not they incorporate data fusion between count data and presence-only data derived from GBIF. This comprehensive evaluation of model types allows for an analysis of the contribution of each component to predictive performance and provides insight into whether ISDMs improve predictive capacity by sharing information across species and/or data currencies.

**Table 1:** Description of the structure of the evaluated models, broken down by their components and/or estimation methods.

Model	Data fusion	Joint- model structure	Shared fixed effects estimation	Indep. fixed effects estimation	Spatial effect (SPDE)	Shared st-effect (SPDE-AR)	Indep. st-effect (SPDE-ARj)	Spatial Varying Effects (SVC)
M1	X	X	X	X	X	X	X	X
M2	X	X	✓	X	✓	X	X	X
M3	X	X	✓	X	X	✓	X	X
M4	X	✓	✓	X	X	✓	X	X
M5	X	✓	✓	X	X	✓	X	X
M6	X	✓	✓	X	X	X	✓	X
M7	X	✓	✓	X	X	✓	X	✓
M8	X	✓	✓	X	X	X	✓	✓
M9	✓	✓	✓	X	X	✓	X	X
M10	✓	✓	✓	X	X	X	✓	X
M11	✓	✓	X	✓	X	✓	X	X
M12	✓	✓	X	✓	X	X	✓	X
M13	✓	✓	✓	X	X	✓	✓	X
M14	✓	✓	X	✓	X	✓	✓	X
M15	✓	✓	✓	X	X	✓	X	✓
M16	✓	✓	✓	X	X	X	✓	✓
M17	✓	✓	X	✓	X	✓	X	✓
M18	✓	✓	X	✓	X	X	✓	✓
M19	✓	✓	✓	X	X	✓	✓	✓
M20	✓	✓	X	✓	X	✓	✓	✓
M21	X	✓	✓	X	X	✓	✓	X
M22	X	✓	X	✓	X	✓	✓	X

The symbol “✓” indicates that the model incorporates the corresponding structure, while “X” denotes its absence. The “Data fusion” category refers to the simultaneous integration of structured count data with presence-only data obtained from GBIF. “Joint-model structure” indicates that the model is built using multiple likelihood functions—one for each species and data type (count or presence)—representing distinct observational processes.

A Leave-Population Out Cross Validation (LPOCV) strategy, a type of Leave Group Out Cross Validation (LGOCV), was implemented to evaluate model predictive performance. The literature suggests that LGOCV is a more appropriate alternative than Leave-One-Out Cross Validation (LOOCV) for models incorporating structured random effects (Adin et al., 2024). In this study, LPOCV was applied using the methodological framework recently proposed by Liu and Rue (2025), which allows the computation of cross-validation metrics without the need to refit models for each resampling iteration. Under the LPOCV strategy, for each observation in the count dataset, its Posterior Predictive Distribution (PPD) is estimated by leaving out from the training set all observations from the same population to which the corresponding grid cell belongs (Equation 6). This exclusion is implemented to avoid highly correlated information, resulting from the proportional allocation of individuals from the same population across multiple grid cells based on area of intersection, from leading to overly optimistic estimates of model predictive performance. Thus, the approach yields a more realistic evaluation by removing structural dependencies between the held-out group and the target observation.

(6) $$\pi (Y_{i}\;|\;y_{-I_{gi}})=\int \pi (Y_{i}\;|\Theta ,\;y_{-I_{i}})\pi (\Theta \;|\;y_{-I_{i}})\;d\Theta$$

Where Yi​ is the observed value, and π(Y_i_∣y_−Igi_) denotes its PPD computed by excluding from the training dataset all observations belonging to the same population g_i_​, according to the structure I_gi_ ( $y_{-I_{gi}}$).

The predictive performance of the model, based on the PPDs of each observation, is assessed using the LPOCV-based log-score function (hereafter, log-score) (Equation 7).

(7) $$log-score=\frac{1}{n}\sum_{i=1}^{n}log\;\pi (Y_{i}\;|\;y_{-I_{gi}})$$

Higher values of this metric indicate that the model assigns greater probability to the observed data, which translates into improved predictive performance. In addition to the log-score, the Watanabe-Akaike Information Criterion (WAIC) (Watanabe and Opper, 2010), model likelihood, and computational time required for model fitting were reported for each evaluated model.

## 3 Results

### 3.1 Model comparison

The results presented hereafter refer to the reduced study area, due to the high uncertainty associated with model estimates in regions distant from observation points. Results concerning the posterior mean and the 95% coverage probability of the log-intensity distribution for the full-size study area from M13, M16 and M21 can be found in **Supplementary Figures S3**-**S8**.

Although both the marginal likelihood and WAIC are reported, model selection was primarily based on the log-score due to its suitability for evaluating predictive performance on new populations, as defined by the LPO-CV framework. Model performances can be consulted in **Table 2**. A substantial improvement in this metric is observed with the inclusion of spatial effects (M2) and further enhanced by spatio-temporal effects (M3), compared to the baseline model without random effects (M1). Fitting models under a ISDMs framework also improves predictive capacity (M3 vs. M4 and M5).

**Table 2:** Comparison of model performance and computation time.

Model	Model likelihood	Log-score	WAIC	Time (h)
M1	-3268,95	-3,097	6421,40	0,0002
M2	-3072,05	-2,837	5909,57	0,0034
M3	-2978,12	-2,495	5589,96	0,42
M4	-2831,04	-2,467	5159,56	2,99
M5	-2932,93	-2,574	5455,71	36,98
M6	-2737,65	-2,317	4844,30	5,69
M7	-2788,28	-2,383	4996,23	1,28
M8	-2744,50	-2,316	4842,38	13,95
M9	-2835,38	-2,465	17910,68	0,82
M10	-2863,61	-2,324	5433,63	5,03
M11	-3357,42	-2,450	5942,29	0,80
M12	-2948,45	-2,307	5231,95	7,45
M13	-2945,78	-2,299	5238,07	13,89
M14	-2946,02	-2,300	5233,45	13,28
M15	-2793,26	-2,381	18829,73	3,13
M16	-2869,45	-2,305	5320,26	9,67
M17	-3318,44	-2,384	5767,31	1,75
M18	-2954,78	-2,321	5358,63	12,05
M19	-2948,34	-2,457	5556,05	27,51
M20	-2948,63	-2,456	5553,06	28,09
M21	-2824,329	-2,297	4798,11	12,45
M22	-2824,341	-2,297	4798,19	13,30

Among the models that do not perform data fusion (i.e., excluding GBIF data), those incorporating both a shared spatio-temporal effect across species and species-specific spatio-temporal effects achieve the best performance (M21 and M22). The inclusion of SVC effects to model interannual trends does not lead to improved predictive capacity.

Within the set of models that incorporate data fusion (M9–M20), a similar pattern is observed. The best predictive performance is achieved by models that include both joint and species-specific spatio-temporal effects. Additionally, no substantial differences are observed between modeling covariate relationships independently for each species or jointly. The inclusion of SVCs in models using data fusion also fails to enhance predictive capacity and, in fact, leads to a notable decrease in log-score when combined with joint and species-specific spatio-temporal effects (M19 and M20).

Overall, the results suggest that data fusion does not improve model predictive performance (M13 vs. M21). Models combining shared and species-specific spatio-temporal effects consistently yield the best results. Consequently, results are presented for these models, along with M16, since as highlighted in the literature (e.g., Brodie et al., 2020; Thorson et al., 2023), SVCs typically do not improve predictive performance, but their value lies in their ability to capture context-dependent covariate effects, thereby providing nuanced insights into ecological processes.

### 3.2 Hyperparameter estimates

Overdispersion estimates were consistent and certain across the three models (**Table 3**). The inclusion of shared spatio-temporal effects across species (in M13 and M21, compared to M16) led to a reduction in the range and marginal standard deviation of species-specific spatio-temporal effects, as these now primarily account for deviations from the common pattern. All spatio-temporal effects, except for *P.incompleta* under M21, showed strong positive temporal autocorrelation, with values close to one. In the case of *P.incompleta* under M21, the 95% credible interval included zero, suggesting the absence of a consistent temporal pattern.

**Table 3:** Comparison of hyperparameter estimates for models M13, M16, and M21.

Hyperparameter estimates	M13	M16	M21
Overdispersion of the negative-binomial likelihood *D. caudatum*	0.05 [0.03, 0.09]	0.05 [0.03, 0.09]	0.05 [0.02, 0.08]
Shared ST-effect			
Range	72143 [14488, 239005]	—	20296 [9546, 38303]
Standard deviation	2.70 [0.58, 7.55]	—	5.14 [2.70, 9.02]
Temporal autocorrelation	1.00 [0.98, 1.00]	—	1.00 [0.99, 1.00]
*Culcita macrocarpa* ST-effect			
Range	3905 [2294, 6288]	4688 [2855, 7145]	2846 [1506, 4875]
Standard deviation	3.83 [2.47, 5.73]	5.38 [3.74, 7.50]	2.01 [1.25, 3.05]
Temporal autocorrelation	0.99 [0.98, 1.00]	0.99 [0.99, 1.00]	0.97 [0.93, 0.99]
*Diplazium caudatum* ST-effect			
Range	3619 [2107, 5838]	10587.33 [6019, 17542]	3881 [1717, 7729]
Standard deviation	3.59 [2.30, 5.35]	8.32 [5.14, 13.06]	1.63 [0.86, 2.87]
Temporal autocorrelation	0.99 [0.98, 1.00]	1.00 [0.99, 1.00]	0.93 [0.81, 0.99]
*Pteris incompleta* ST-effect			
Range	6200 [3721, 9764]	10042 [6033, 15797]	6690 [2544, 14418]
Standard deviation	5.51 [3.38, 8.57]	7.56 [4.73, 11.52]	0.42 [0.28, 0.61]
Temporal autocorrelation	0.99 [0.99, 1.00]	0.99 [0.99, 1.00]	-0.33 [-0.72, 0.17]
SVC-effect			
Range	—	3641.88 [1395, 8196]	—
Standard deviation	—	0.12 [0.06, 0.22]	—
Beta for *D. caudatum*	—	1.46 [0.96, 1.96]	—
Beta for *P. incompleta*	—	0.75 [0.20, 1.31]	—

Results are reported as the posterior mean along with the 0.025 and 0.975 quantiles. ST refers to the spatio-temporal effect, modeled using an SPDE-AR approach. SVC denotes a Spatially Varying Coefficient effect.

The SVC in model M16 was estimated to have a shorter spatial range than the other spatio-temporal effects, implying it operates at a finer spatial scale. Interannual dynamics associated with the SVC effect showed similar patterns across species, as reflected in the positive shared-effect β estimates.

Data fusion (M13 vs M21) resulted in an increased range for the shared spatio-temporal effect and a corresponding reduction for the independent species-specific effects. The shared spatio-temporal effect under M13 was estimated with nearly an order of magnitude greater 95% coverage range than in M21 (224,517 vs. 28,757). Regarding the species-specific spatio-temporal effects, a general decrease in the 95% credible interval was observed when moving from M13 to M21: from 3994 to 3369 for *C.macrocarpa*, from 3731 to 6012 for *D. caudatum*, and from 6042 to 11,873 for *P. incompleta.*

### 3.3 Fixed effects estimates

**Figure 2:**
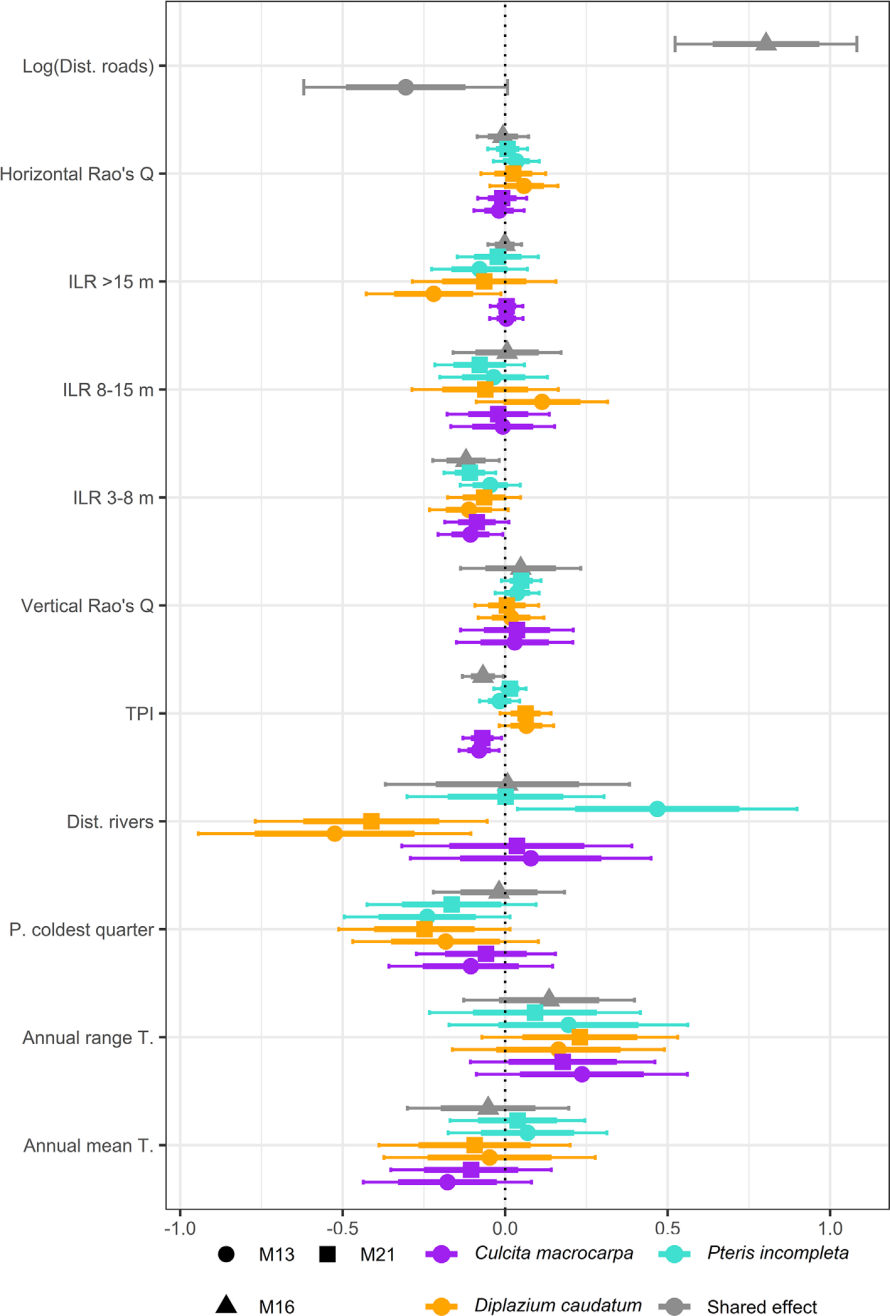
Summary of fixed effect estimates for *Culcita macrocarpa*, *Diplazium caudatum*, and *Pteris incompleta* obtained from models M13, M16, and M21. Model M16 provides joint estimates for all covariates due to its structural specification. For the covariate *log(distance to roads)*, estimates are always shared across GBIF-derived data, and the covariate is absent in M21 since this model relies exclusively on count data.

Overall, there are no major differences in the fixed effect estimates across models, and the estimates are generally consistent among the three species, except for certain covariate relationships (**Figure 2**). The estimates exhibit considerable uncertainty, and most effects are not significant understood in a Bayesian context as those whose 95% credible intervals include zero. Detailed numerical estimates can be found in **Supplementary Table S1**. Models M13, M16, and M21 estimate a negative relationship between TPI and intensity. This indicates a lower intensity in steeper terrains and ridges, and a preference for habitats located in valleys and canyons. Additionally, an increase in the dominance of the 3–8 m tree canopy stratum is associated with lower individual abundance. There is substantial variability in the estimated effect of distance to riparian zones across models and species. Both M13 and M21 estimate a negative relationship between D. caudatum intensity and distance to rivers, suggesting greater abundance in areas closer to watercourses. In contrast, no credible effect is observed for *C. macrocarpa*, and for *P. incompleta.* M13 estimates a negative relationship, indicating that for the populations analyzed, intensity is higher in areas not immediately adjacent to streams. Distance to rivers effect, when estimated as a shared effects, are not considered credible, as the joint estimation across the three species, with opposing responses, results in estimates centered around zero. Regarding effects estimated credible in specific models only, M13 finds a significant negative relationship between *D. caudatum* intensity and the proportion of canopy composed of trees taller than 15 meters. Meanwhile, M16 estimates a positive effect of the logarithm of distance to access roads.

Although not significant at the 95% level, the 75% credible intervals of the posterior distributions of fixed effects have been reported. Mean annual temperature generally shows a negative association with species abundance, except in the case of *P. incompleta*. For *C. macrocarpa*, M13 does estimate a likely negative effect at the 75% level. Consistently, and despite some uncertainty, all three species show a positive relationship with the annual temperature range, with this effect being likely (i.e., 75% credible interval not including zero) for *C. macrocarpa* and *D. caudatum* under models M13 and M21. Likewise, intensity tends to be negatively associated with precipitation during the coldest quarter. This effect is considered credible for *D. caudatum* and *P.incompleta* under models M13 and M21. *D. caudatum* shows a consistent positive association with TPI across models at the 75% level. Regarding canopy structure variables, although generally not significant, they tend to be estimated with lower uncertainty compared to climatic covariates. Vertical structure shows a positive relationship with intensity, with the 75% credible interval excluding zero only in the case of *P. incompleta* under model M21. Among the remaining canopy variables, only the relative dominance of the >15 m stratum is negatively associated with *D. caudatum* intensity according to model M13.

### 3.4 Predicted spatial patterns

**Figure 3:**
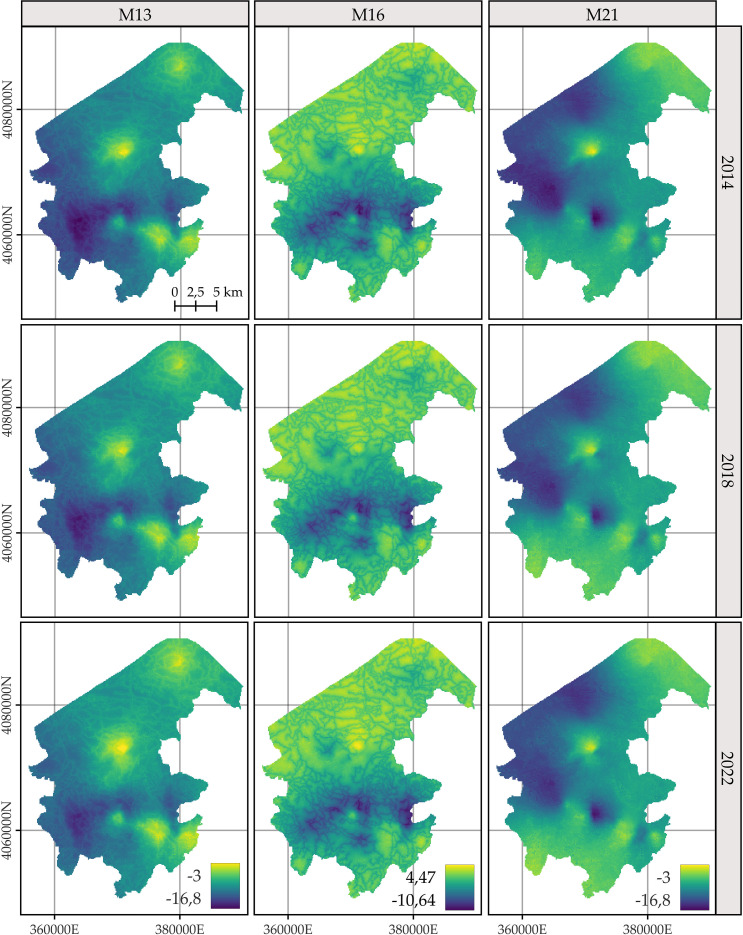
Comparison of the log-intensity of the state equation for *Culcita macrocarpa* for the years 2014, 2018, and 2022. Note that model-specific legends have been used, which are consistent across years within each model, to highlight the temporal evolution captured by each approach. For further details on the year-by-year temporal dynamics, refer to **Supplementary Figures S3**, **S5**, and **S7**.

**Figure 4:**
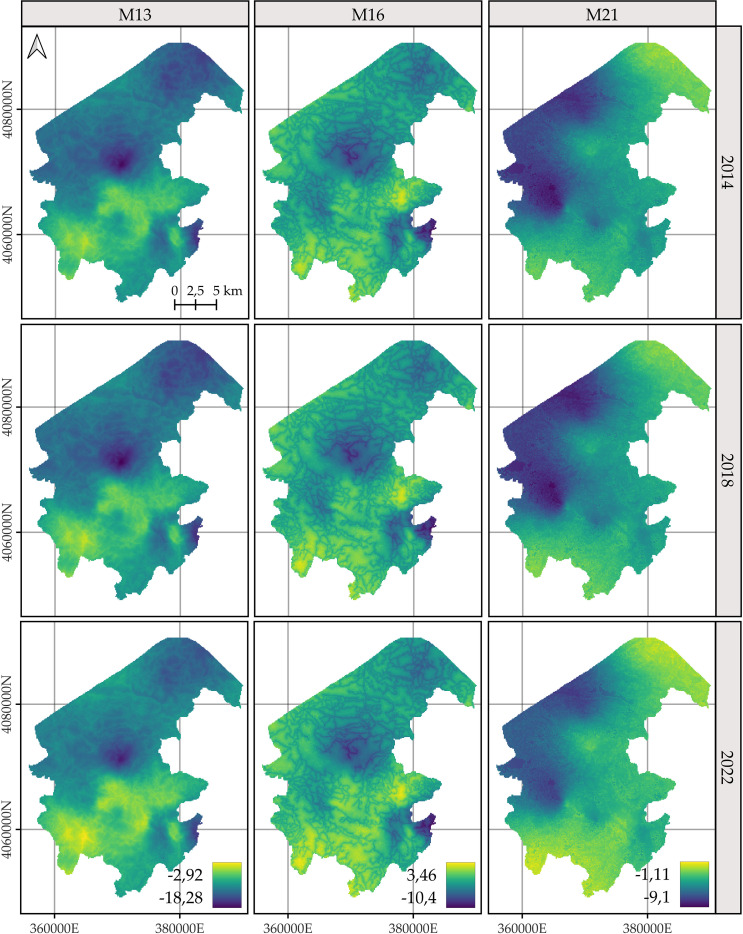
Comparison of the log-intensity of the state equation for *Diplazium caudatum* for the years 2014, 2018, and 2022. Note that model-specific legends have been used, which are consistent across years within each model, in order to highlight the temporal evolution captured by each approach. For further details on the year-by-year temporal dynamics, refer to **Supplementary Figures S3**, **S5**, and **S7**.

**Figure 5:**
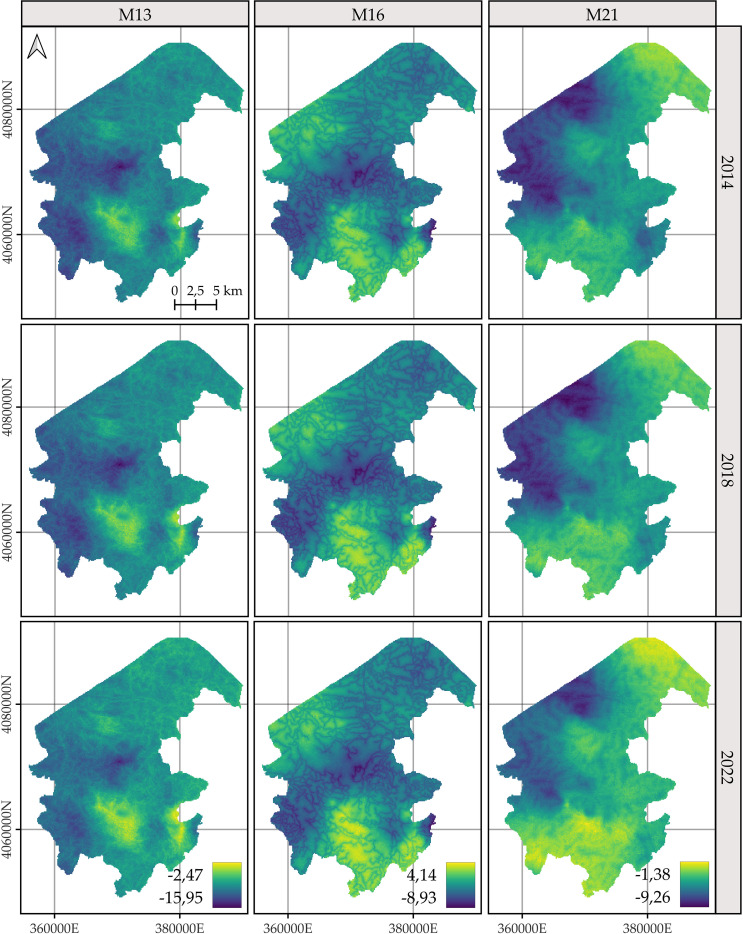
Comparison of the log-intensity of the state equation for *Pteris incomplete* for the years 2014, 2018, and 2022. Note that model-specific legends have been used, which are consistent across years within each model, in order to highlight the temporal evolution captured by each approach. For further details on the year-by-year temporal dynamics, refer to **Supplementary Figures S3**, **S5**, and **S7**.

For *C. macrocarpa*, *D. caudatum*, and *P. incompleta*, the predicted log-intensity maps for the years 2014, 2018, and 2022 are shown in **Figures 3**–**5**, respectively. **Supplementary Figure S9** presents a comparative visualization of the log-intensity by model for the year 2022, allowing for a side-by-side comparison of spatial patterns across the three species. For all three species (**Figures 3**–**5**), regardless of the model considered, clear stability in spatial patterns is observed over the years. Generally, the largest increases in log-intensity occur in areas that were already identified as high-intensity zones in 2014. Models M13 and M21 produce more similar spatial patterns to each other than to those of M16. Furthermore, M16 generates a wider range of estimated log-intensity values compared to the ranges observed between M13 and M21. This corresponds with its lower predictive capacity as shown in **Table 2**. In the case of *C. macrocarpa* (**Figure 3**), these differences are much more pronounced, with M16 substantially overestimating log-intensity in the northern and northwestern parts of the study area. For *D. caudatum* and *P.incompleta* (**Figures 4**, **5**), results from M16 show greater similarity to the other models, but it still predicts high log-intensity values in areas where the other two models do not.

The spatial patterns produced by M16 appear to be more influenced by fixed effects, particularly the distance to roads, compared to M13 and, to a lesser extent, M21, where the influence mainly arises from proximity to rivers. In models M13 and M21, the contribution of the space-time random effects exceeds that of the fixed effects. This pattern seems reversed in M16, where fixed effects have a high influence on the contribution to the log-intensity (**Supplementary Figures S10**–**S12**). The estimation of the shared space-time random effect among the three species under M13 (**Supplementary Figure S10**) results in a smaller magnitude, which can be interpreted as a residual spatial pattern, compared to the pattern estimated under M21 (**Supplementary Figure S12**). In M21, the shared effect captures the common spatial variation in the locations of observations across species, while the species-specific space-time effects represent deviations of each species from this common pattern. This situation appears to be inverted in M13, where the species-specific space-time random effects fully model the behavior of each species, and the shared random effect seems to reflect residual variation in the model.

The results of M13 compared to those of M21, for any of the species, show a more localized spatial pattern, where areas of higher log-intensity coincide with locations where observations exist (**Supplementary Figure S9**). M21 exhibits greater information sharing between species, since for a given species there are areas where M13 estimates a low log-intensity, but M21 estimates an increase in log-intensity associated with the presence of observations of the other two species. This pattern is also observed in the opposite direction, where areas lacking two species lead to lower log-intensity estimates under M21 for the remaining species compared to those from M13. This effect is especially noticeable when comparing M21’s estimates for *D. caudatum* and *P. incompleta* in the northern part of the study area with those of M13. Similarly, for *C. macrocarpa*, M21 tends to estimate higher log-intensity in the southwestern area than M13. Conversely, M21 estimates lower density in the western and northwestern areas compared to M13. Thus, M21 produces smoother estimates with greater information sharing between species than M13.

**Figure 6:**
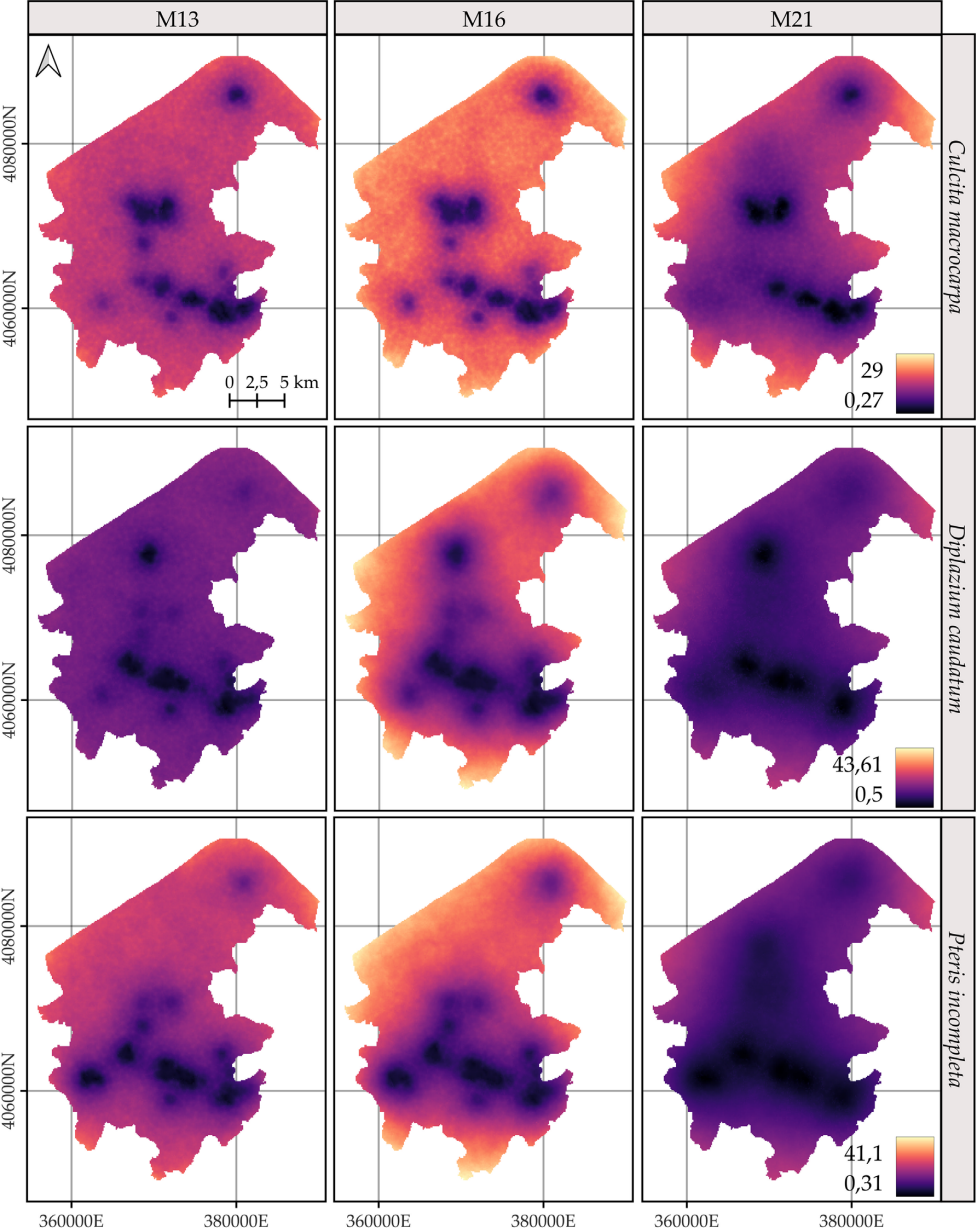
Comparison of the 95% coverage probability of the log-intensity for the three species. Due to the similarity in spatial patterns across years, only the results for 2022 are shown. Note that model-specific legends have been used, consistent across years within each model, to emphasize the temporal evolution captured by each approach. For further details on year-by-year dynamics, refer to **Supplementary Figures S4**, **S6**, and **S8**.

The spatial patterns exhibit considerable uncertainty regardless of the model. In general, species predictions are more reliable in areas closer to the species’ own observation points and, to a lesser extent, to those of the other species (**Figure 6**). M21 shows the lowest uncertainty for all species, as represented by the 95% credible interval of the posterior distribution of the log-intensity.

### 3.5 Net spatial change in log-intensity

**Figure 7:**
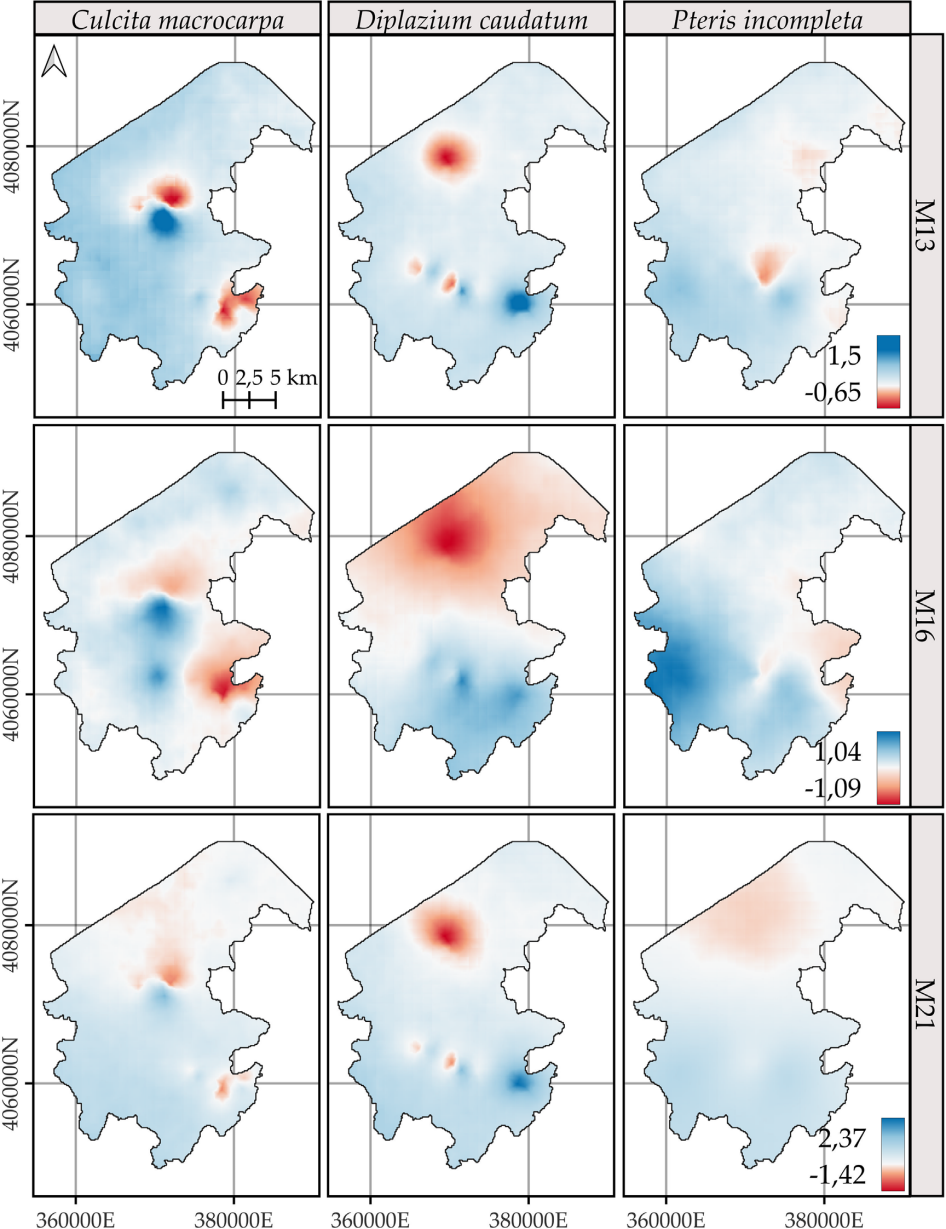
Comparison of the posterior mean of the difference in log-intensity between the years 2023 and 2014. Positive values represent areas with an increase in log-intensity over this period, while negative values indicate a decrease. Model-specific legends have been used to enhance the visualization of spatial change patterns for *Culcita macrocarpa*, *Diplazium caudatum*, and *Pteris incompleta*. For more detailed information, refer to species-specific figures: **Supplementary Figures S13**, **S14**, and **S15**.

**Figure 8:**
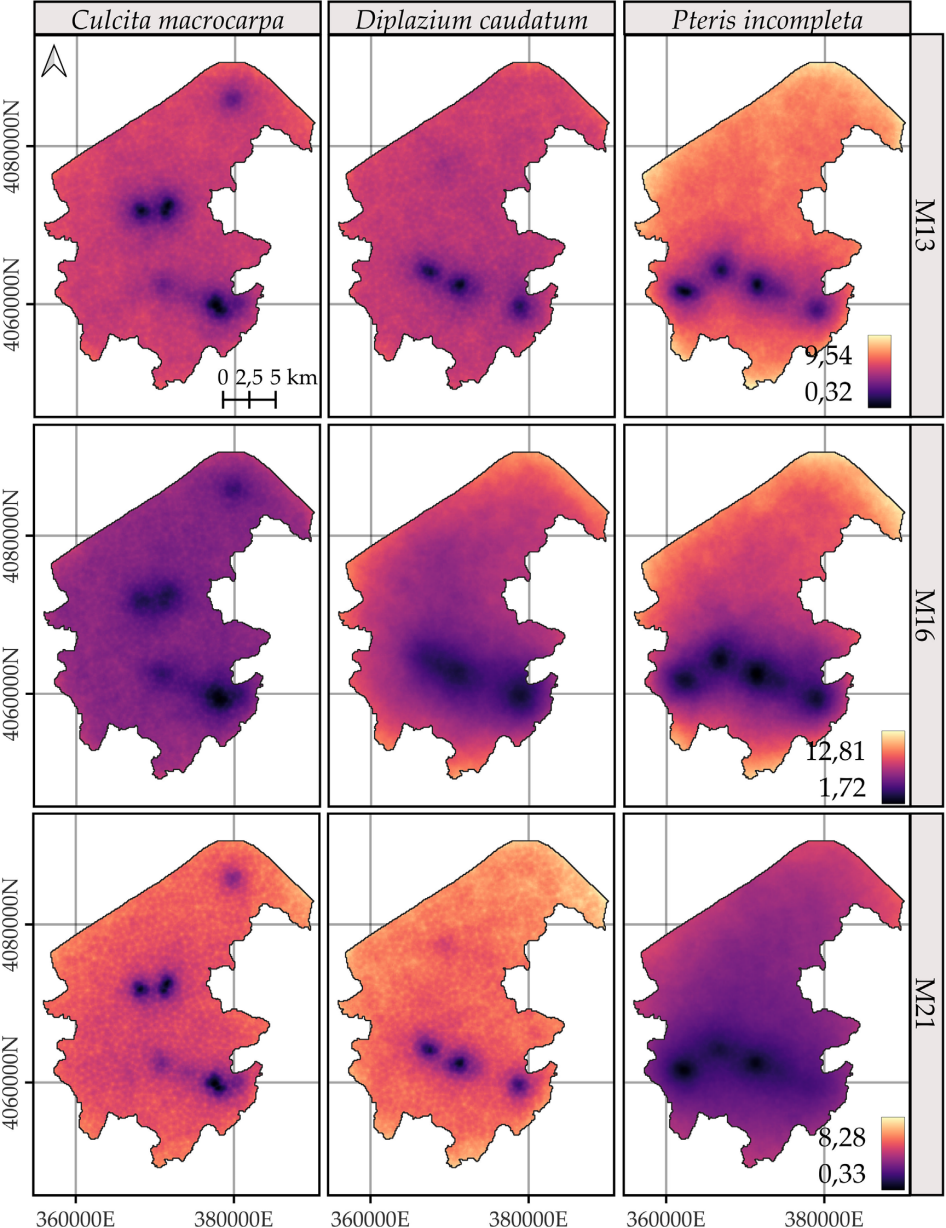
Comparison of the posterior 95% coverage probability interval of the difference in log-intensity between the years 2023 and 2014. Model-specific legends have been used to enhance the visualization of spatial change patterns for *Culcita macrocarpa*, *Diplazium caudatum*, and *Pteris incompleta*. For more detailed information, refer to species-specific figures: **Supplementary Figures S13**, **S14**, and **S15**.

The posterior mean and the 95% credible interval of the difference in the logarithm of intensities between the years 2023 and 2014 are shown in **Figures 7** and **8**, respectively. The spatial patterns indicate that the most significant changes over the analyzed decade are concentrated in specific areas associated with the presence of fern observations. Model M13 exhibits the most localized change patterns. Model M16 delineates the largest areas of change, displaying a pattern that is similar to M13 and M21 but more spatially extensive. M21 shows change patterns broadly consistent with those of M13, with the main discrepancies observed in the distribution of *P. incompleta* and in the northernmost area of *C. macrocarpa* (**Figure 7**).

Unlike the annual log-intensity patterns, the 95% credible interval for the difference does not appear to be primarily driven by the joint spatial distribution of all species (**Figure 8**). Instead, the uncertainty in the estimated change is largely confined to the spatial locations where species abundances were observed. The resulting uncertainty ranges reach an amplitude of nearly 13 (in terms of log intensity), which is substantially greater than the maximum observed magnitude of change, approximately 2.4.

**Figure 9:**
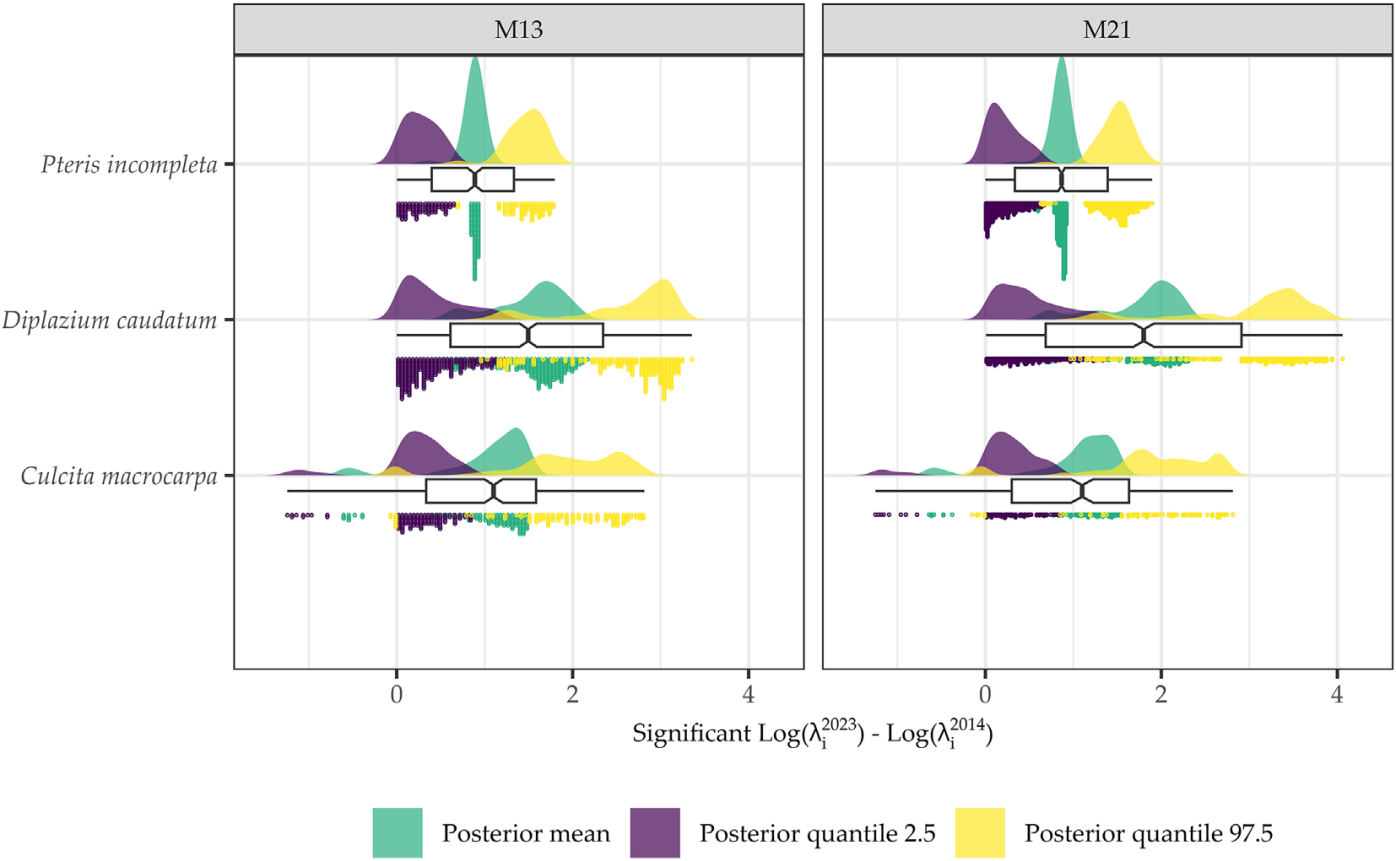
Statistical distributions of the posterior mean, 2.5% quantile, and 97.5% quantile values for the difference between 2023 and 2014 log intensities at locations identified as significant, i.e., where the 95% credible interval of the posterior distribution does not include zero for *Culcita macrocarpa, Diplazium caudatum*, and *Pteris incompleta*, as estimated by models M13 and M21.

Accordingly, when focusing on statistically significant areas (**Supplementary Figures S13**–**S15**), i.e., regions where the 95% credible interval for the difference in log intensities does not include zero, virtually the entire study area shows no change in log intensity. Model M16 does not estimate any change for any species in any location. A consistent pattern emerges between M13 and M21 in the delineation of areas with significant change, and in all cases, these areas correspond to locations with observed data. Comparisons of differences in log intensities across all areas with significant changes are shown in **Figure 9**. Overall, both the spatial patterns and the magnitude of change are consistent between models M13 and M21. Only *C. macrocarpa* exhibits areas of significant negative change (**Supplementary Figure S13**), with the mean across sites of the posterior means, and the 2.5th and 97.5th percentiles of the log-intensity difference estimated at −0.52 [−1.04, −0.02] for model M13 and −0.54 [−1.07, 0.06] for model M21. Areas showing an increase in the log intensity of *C. macrocarpa*, restricted to a single spatial location, present values of 1.18 [0.33, 2.03] for M13 and 1.18 [0.31, 2.04] for M21. For *D. caudatum*, three distinct populations with significant change are estimated. It also shows the highest net increase in log intensity, with values of 1.5 [0.41, 2.59] for M13 and 1.75 [0.48, 3.03] for M21. *P. incompleta* is the species with the smallest observed change, with estimated values of 0.87 [0.28, 1.47] for M13 and 0.85 [0.22, 1.49] for M21. Notably, it also shows the greatest model discrepancy, as M21 identifies significant areas of greater extent, including one that is not detected by M13.

### 3.6 Temporal interpolation capability

**Table 4** summarizes the temporal interpolation performance of models M13 and M2. Regarding the posterior coverage probability intervals (pPCPI-I), M21 consistently achieves higher percentages of observations captured at both the 75% and 95% levels. With respect to the amplitude of the 75% and 95% posterior intervals, M21 generally produces narrower intervals than M13 across species for observed data. Across observations there is also less variability in the amplitudes for M21 than for M13. The posterior predictive distribution coverage intervals (pPCPI-PPD) also favors M21, with a higher proportion of observed data being captured. The similarity in mean amplitude, particularly in light of the high coverage percentages observed, especially for M21, suggests that the residual variation not accounted for in the state model (i.e., the intensity) is effectively captured by the observation model component. For missing time points, the behavior of the two models differs substantially. M21 produces wider PCPI-I and PPD intervals, with higher associated standard deviations across all species. In contrast, M13 yields narrower intervals with lower variability. These narrower intervals in M13 are accompanied by lower coverage percentages compared to M21, particularly at the 95% level. This pattern, consistent across all three species and most pronounced in *P. incompleta*, indicates that M21 produces larger and variable uncertainty estimates under data missing conditions. The mean absolute error (MAE) between the observed counts and the posterior mean of the intensity is markedly lower in M21 for all species, indicating better point prediction alignment overall for M21.

**Table 4:** Comparison of temporal interpolation capacity by species for models M13 and M21.

Performance metrics	*Culcita macrocarpa*		*Diplazium caudatum*		*Pteris incompleta*	
	M13	M21	M13	M21	M13	M21
75% pPCPI-I	2.61	58.49	7.56	53.49	3.87	59.35
95% pPCPI-I	33.16	71.8	45.35	70.93	29.03	66.77
95% pPCPI-PPD	82.51	97.91	77.33	93.02	72.90	90.00
Mean 75% PCPI-I amplitude	3.98	3.03	4.10	3.96	6.07	3.55
Mean 95% PCPI-I amplitude	9.02	5.19	9.48	6.83	14.02	6.07
Mean 95% PCPI-PPD amplitude	10.29	10.88	10.96	15.71	15.46	14.37
Sd. 75% PCPI-I amplitude	6.92	3.51	5.22	4.45	7.63	3.41
Sd. 95% PCPI-I amplitude	15.61	5.97	12.01	7.61	17.62	5.79
Sd. 95% PCPI-PPD amplitude	16.02	9.77	12.71	14.79	18.15	10.01
Mean 75% PCPI-I amplitude for NAs	4.60	9.37	4.10	10.07	7.62	13.63
Mean 95% PCPI-I amplitude for NAs	11.08	17.48	9.88	19.62	18.20	24.92
Mean 95% PCPI-PPD amplitude for NAs	12.06	20.60	11.23	24.72	19.38	29.19
Sd. 75% PCPI-I amplitude for NAs	7.56	14.01	4.18	10.35	11.44	21.20
Sd. 95% PCPI-I amplitude for NAs	17.88	25.92	9.86	20.29	26.66	38.05
Sd. 95% PCPI-PPD amplitude for NAs	17.89	26.73	10.43	22.49	26.80	38.76
MAE	7.31	1.56	7.81	2.31	11.99	2.8

pPCPI-I refers to the percentage of observations covered by the X% posterior coverage probability interval of the intensity. pPCPI-PPD denotes the percentage of observations covered by the X% posterior predictive distribution coverage probability interval. Nas refers to missing data within the temporal time series. MAE is the Mean Absolute Error calculated between observed count data and the posterior mean of the intensity.

**Figure 10:**
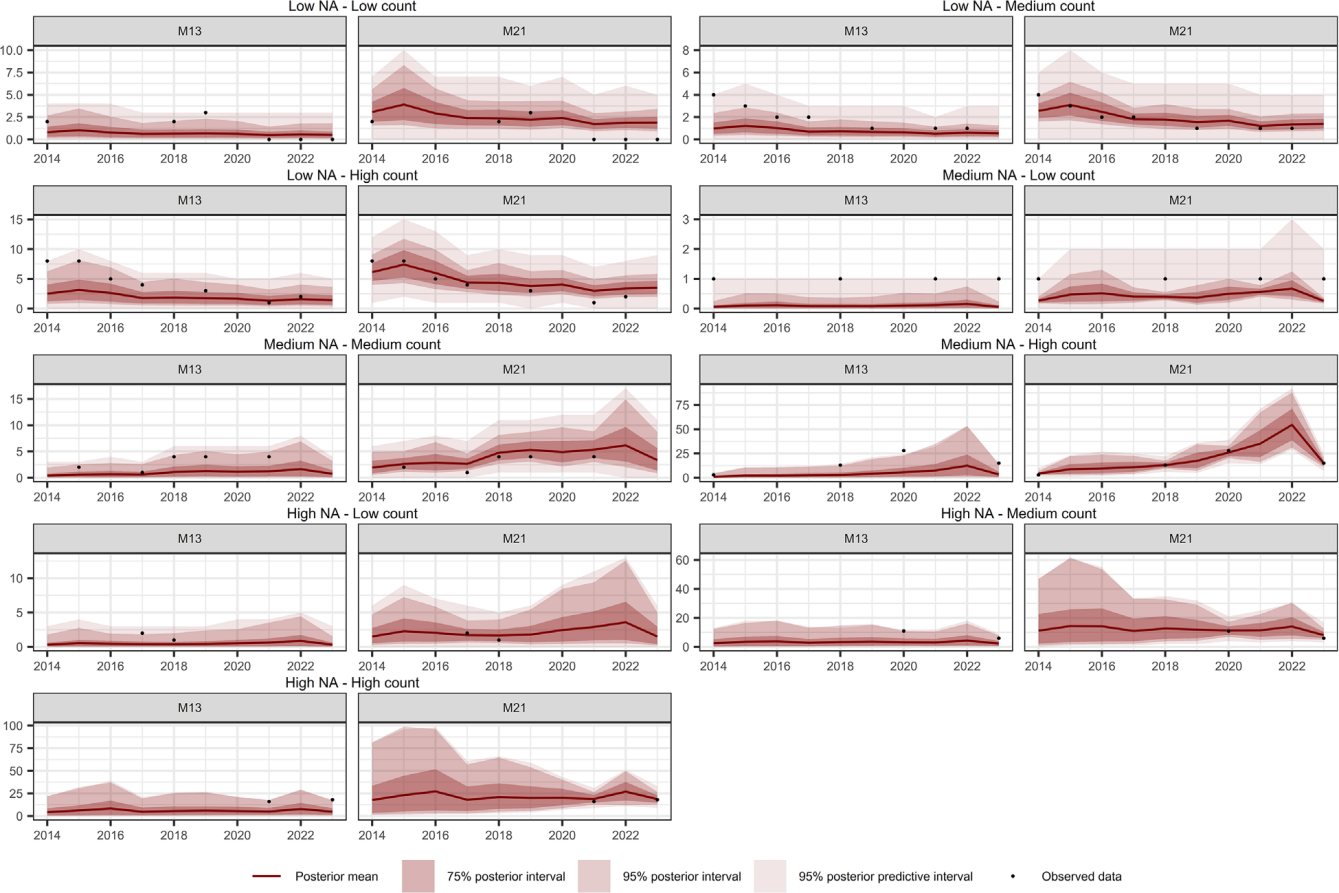
Comparison of temporal interpolation of abundance for *Culcita macrocarpa* grid cells between models M13 and M21. As an illustration, time series were randomly selected as representative of the following groups: Low NA – Low Count, Low NA – Medium Count, Low NA – High Count, Medium NA – Low Count, Medium NA – Medium Count, Medium NA – High Count, High NA – Low Count, High NA – Medium Count, and High NA – High Count. The NA typology refers to the proportion of missing values in the time series, while the Count typology refers to the average abundance across the series. Grouping was performed by dividing the time series based on quantiles of both the missing data proportion and the average abundance, in order to capture a range of interpolation scenarios and evaluate model performance across different conditions.

**Figure 11:**
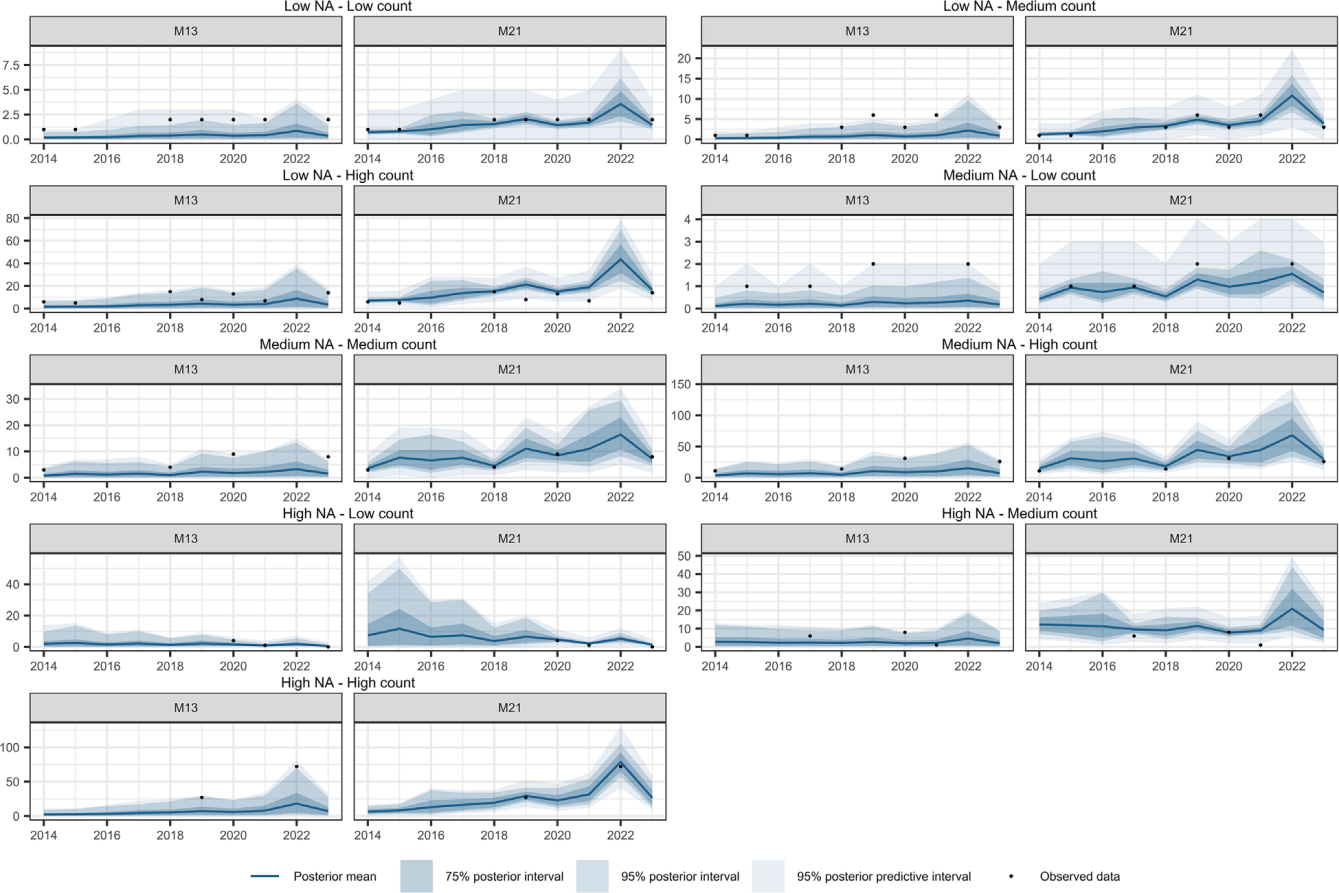
Comparison of temporal interpolation of abundance for *Diplazium caudatum* grid cells between models M13 and M21. As an illustration, time series were randomly selected as representative of the following groups: Low NA – Low Count, Low NA – Medium Count, Low NA – High Count, Medium NA – Low Count, Medium NA – Medium Count, Medium NA – High Count, High NA – Low Count, High NA – Medium Count, and High NA – High Count. The NA typology refers to the proportion of missing values in the time series, while the Count typology refers to the average abundance across the series. Grouping was performed by dividing the time series based on quantiles of both the missing data proportion and the average abundance, in order to capture a range of interpolation scenarios and evaluate model performance across different conditions.

**Figure 12:**
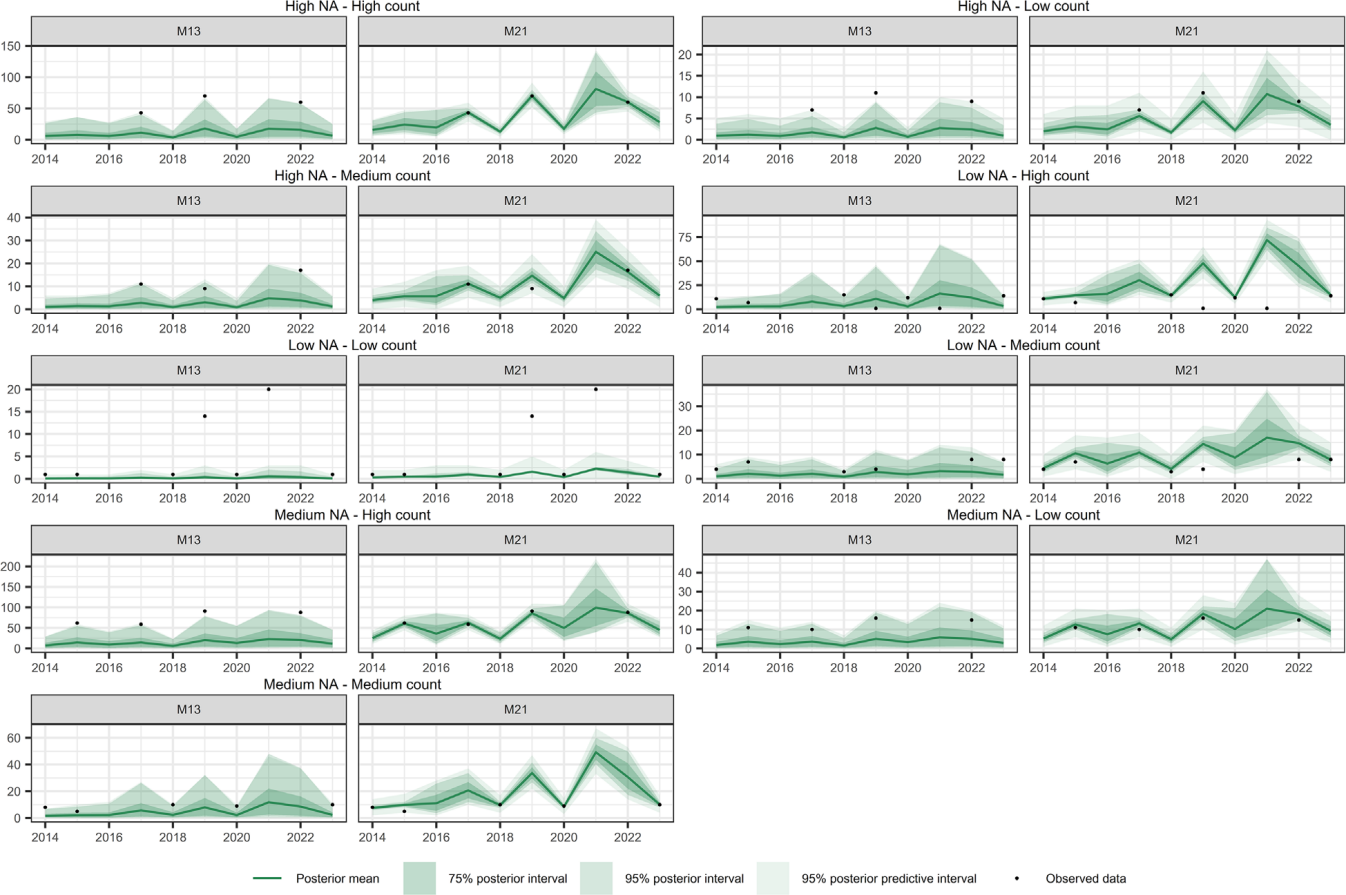
Comparison of temporal interpolation of abundance for *Pteris incompleta* grid cells between models M13 and M21. As an illustration, time series were randomly selected as representative of the following groups: Low NA – Low Count, Low NA – Medium Count, Low NA – High Count, Medium NA – Low Count, Medium NA – Medium Count, Medium NA – High Count, High NA – Low Count, High NA – Medium Count, and High NA – High Count. The NA typology refers to the proportion of missing values in the time series, while the Count typology refers to the average abundance across the series. Grouping was performed by dividing the time series based on quantiles of both the missing data proportion and the average abundance, in order to capture a range of interpolation scenarios and evaluate model performance across different conditions.

**Figures 10**–**12** show the temporal interpolation results for *C. macrocarpa*, *D. caudatum*, and *T. incompleta*, respectively, across nine randomly selected locations. These locations were chosen to represent a range of conditions in terms of missing data and average abundance over the decade, with the aim of evaluating scenarios where model performance is most challenged. Across all species, the posterior mean time series estimated by M21 tends to align more closely with the observed count data compared to M13. In general, both models exhibit poorer performance in locations with lower overall abundance, particularly in time series where the average count is close to one individual per year. Uncertainty ranges are generally wider under M21, although larger posterior coverage probability intervals are commonly observed in time series with high proportions of missing data or extended data gaps for both models. In the case of *P. incompleta*, strong interannual fluctuations are evident in the posterior estimates, driven primarily by the negative estimate of the temporal autocorrelation parameter in the species-specific spatiotemporal random effect.

**Supplementary Figures S16**–**21** display the posterior distributions of population-level intensity and abundance, where abundance was simulated from the PPD, according to population membership. As summarized in **Supplementary Table S2**, model M21 consistently outperforms M13 in terms of temporal interpolation accuracy across all three species. It achieves notably higher posterior coverage rates, especially under data-scarce conditions, while maintaining narrower predictive intervals and lower mean absolute errors, suggesting a more reliable estimation of latent intensity and uncertainty. However, the temporal interpolation of abundance shows poorer performance when aggregated at the population level compared to grid-level estimates.

## 4 Discussion

ISDMs have emerged as a key tool for integrating diverse data sources and multiple species within a single model, enabling ecologists and researchers to develop a more detailed and nuanced understanding of the factors driving species distributions. In this study, 22 ISDMs as conceptualized by Isaac et al. (2020), extended into a spatio-temporal framework aimed at modeling the distribution and temporal variation of *C. macrocarpa*, *D. caudatum*, and *P. incompleta* populations in Los Alcornocales Natural Park. The state-space framework enabled the integration of multiple data sources by incorporating distinct species and observation models, explicitly accounting for the underlying processes associated with each dataset. This hierarchical approach allowed to disentangle underlying species distribution from observation-related noise. Standardized and replicable abundance data from the Andalusian Fern Recovery Plan were combined with opportunistic GBIF records, treated as binomial data.

Initially, the evaluated models were calibrated using the entire extent of Los Alcornocales Natural Park. As expected, and in line with the known behavior of Gaussian Fields used to model spatial effects, these models often exhibit reduced performance in areas that are geographically distant from the observational data on which they are trained. This leads to a “distance decay” effect, where predictive accuracy diminishes with increasing distance from training locations, a phenomenon well documented in the literature (Diggle and Ribeiro, 2007; Banerjee et al., 2014). Since the three fern species under study exhibit highly localized distribution ranges, confined to the central-southern portion of the park due to specific microclimatic conditions (Salvo-Tierra, 1990), predictions were restricted to biogeographically plausible areas (Wang et al., 2023).

ISDMs offer clear advantages over single-source approaches, particularly under data limitations or spatial bias (Pacifici et al., 2017; Koshkina et al., 2017; Adde et al., 2021; Dovers et al., 2024). By incorporating multiple data types through joint likelihood frameworks, ISDMs improve model performance and ecological inference while preserving the unique structure of each dataset (Pacifici et al., 2019; Suhaimi et al., 2021; Morera-Pujol et al., 2022). Explicitly modeling observational processes was essential for making optimal use of GBIF data. Citizen science records are often subject to observational biases related to sampling effort and site accessibility (Ver Hoef et al., 2021). This issue significantly affects GBIF data for the three target species in our study area, where observations are heavily clustered around known fern populations. Rare species tend to be underrepresented in citizen science platforms, as they are less likely to be encountered and reported by the general public (Robinson et al., 2017; Fontaine et al., 2022). Instead, expert observers are typically responsible for these records, which introduces sampling bias toward areas where prior knowledge of the species’ presence exists (Chauvier et al., 2021). Additionally, the discovery of new individuals or populations is strongly influenced by accessibility and proximity to roads or trails. Accordingly, a preliminary filtering of GBIF records was conducted to remove outliers and improve the overall quality of the inference. Furthermore, “distance to road” was included as an observational covariate, and its effect was estimated with a 95% probability, confirming the critical role that data collection processes play in shaping distribution estimates. As a next step, it is necessary to calibrate ISDMs that explicitly account for the dependence between sampling locations and species abundance in order to reduce observational bias. However, as noted by Pennino et al. (2019), these methods can be computationally intensive and occasionally unstable, challenges that may be further exacerbated when modeling multiple species simultaneously and incorporating various data currencies.

To date, no studies have applied ISDMs to ferns, making this the first documented case of their application to this plant group. Results indicate that modeling the distribution of the three fern species jointly provides clear benefits. However, integrating GBIF data with count data did not lead to improvements in predictive performance. Regarding spatial generalization capacity M21 showed no differences and even slightly outperformed model M13, which incorporates GBIF data. M13 produced less smooth spatial patterns and exhibited distributions more tightly constrained to the observations. In terms of temporal interpolation capacity, M21 also exhibited lower MAE, reduced uncertainty, and higher coverage of observations within the defined probability intervals. M13 generated narrower predictive intervals for unobserved time points, resulting in overconfident outputs, which may hinder the model’s ability to adequately reflect real variability. Conversely, M21 yielded broader predictive intervals for missing time points, better accommodating the uncertainty associated with unsampled abundance data.

According to our findings, data fusion does not contribute to an improvement in either predictive capacity or inferential strength of the model, primarily due to redundancy in information content between GBIF data and structured count data from the Andalusian Fern Recovery Plan. The strongly constrained distribution ranges of *C. macrocarpa, D. caudatum, and P. incompleta*, all restricted to the central-southern part of Los Alcornocales Natural Park, result in GBIF observations providing minimal additional spatial information beyond what is already captured by the structured count data. While our three target species exhibit characteristics of rare species, restricted geographic ranges, high habitat specialization, and small population sizes (Lavergne et al., 2005; Medrano and Herrera, 2008), their modeling challenges do not align with the rare species modeling paradox as defined by Lomba et al. (2010). This paradox specifically addresses the contradiction that rare species are among the most in need of predictive distribution modeling for effective conservation planning, yet simultaneously the most challenging to model due to inherently limited data. Crucially, the paradox emphasizes that for rare species, every additional occurrence record can provide valuable information for model development, which contradicts our observed lack of benefit from GBIF data integration. As demonstrated in a recent ISDMs study (Dovers et al., 2024), the benefits of data fusion depend critically on the degree to which secondary data sources provide genuinely complementary information to primary datasets. When secondary data sources sample identical or overlapping spatial locations without contributing new environmental gradients or detection processes, integration offers minimal advantages despite potentially increasing computational complexity. In our specific case, the information contributed by GBIF records is effectively redundant, as it is already encapsulated within the structured count data collected through systematic monitoring protocols.

The redundancy of information across the two data currencies for each species may also be a key factor contributing to the more spatially constrained prediction patterns and the overconfident uncertainty intervals of M13. The high spatial proximity between structured count data and GBIF records, and in many cases, their overlap within the same grid cells, may result in over-optimistic predictions due to inflated data density in certain areas. In addition, the estimation of species-specific spatial patterns, when shared across data currencies, may also contribute to the narrowing of predicted ranges. This is likely driven by the same issue of spatial overlap, leading the model to infer more localized spatial distributions concentrated around areas with observed data. As a result, M13 may underestimate the potential range of each species, reinforcing spatial patterns that are overly centered on currently known populations.

Building on the considerations above, the simplification applied to GBIF data likely further contributed to the reduced performance of the data-fusion model (M13) compared to M21. Converting point-based GBIF records into annualized binomial data and assuming their temporal constancy over the decade analyzed represents a necessary trade-off, driven by the extremely limited number of occurrences available (*n* = 47). This replication of static spatial patterns across all years, combined with the informational redundancy with the structured count data, likely underlies the inability of the M13 incorporating data fusion to improve predictive performance. Given its superior performance and simpler structure relative to M13, M21 is therefore preferable for making inferences.

The estimated intensities are modeled as a function of ecological covariates and random effects. The covariates included in the model were selected based on the ecological requirements and niche characteristics of the species as reported in the literature. The vast majority of fern species inhabit low-light environments, typically within or beneath angiosperm canopies (Kawai et al., 2003; Schneider et al., 2004; Schuettpelz and Pryer, 2009; Azevedo-Schmidt et al., 2024). *C. macrocarpa*, *D. caudatum*, and *P. incompleta* exhibit strong ecological convergence, sharing a highly specialized niche defined by pronounced shade tolerance (sciophily) and dependence on high atmospheric and soil moisture (hygrophily). These ferns persist in humid, thermally stable forest understories, typically between 15°C and 30°C, with minimal annual temperature fluctuation (Suárez-Santiago et al., 2024; Elias et al., 2018; Rodríguez-Sánchez, 2011). Their distributions are tightly linked to specific microhabitats such as *canutos*, narrow, north-facing ravines, streambanks, and enclosed valleys, where fog retention, dense vegetation cover, and permanent water sources ensure consistently moist microclimatic conditions throughout the year (Salvo-Tierra & Escámez-Pastrana, 1988; Salvo-Tierra, 1990). Soil characteristics also play a key role: while *C. macrocarpa* tolerates a broad range of lithologies excluding limestone, and can occur in both deep and skeletal soils, *P. incompleta* shows preference for highly acidic, organic-rich substrates, and *D. caudatum* is strongly associated with siliceous environments (Moreno Saiz et al., 2019). All three species are highly sensitive to microclimatic disruptions, particularly during spore germination and early development (Schuler et al., 2021).

Overall, covariate–intensity relationships were found to exhibit high uncertainty. As expected, a negative relationship was observed for annual mean temperature. Proximity to rivers and the TPI, used as proxies for humidity and water availability, showed variable results across species. However, higher intensities were generally observed near rivers and in valley bottoms, as indicated by the TPI. Although credible relationships were found only for the percentage of LiDAR returns within the 3–8 m arboreal stratum, Rao’s Q vertical diversity index exhibited a consistent positive effect. This suggests that, vertical vegetation complexity, a common characteristic of riparian habitats, contributes to fern abundance. Contrary to expectations, and although not a credible effect, ferns showed an increased intensity in areas with greater annual temperature range and lower precipitation during the coldest quarter. It would not be unreasonable to assume that this pattern lies in the ecological characteristics of the gametophyte stage, which may lead to population distributions that deviate from expectations. Although gametophytes are often assumed to be highly sensitive to environmental stress, growing evidence indicates that they can tolerate broader climatic variability than sporophytes (Watkins et al., 2007; Pittermann et al., 2013; Pinson et al., 2017). This hidden gametophyte presence can facilitate sporophyte regeneration across a broader environmental gradient than anticipated, leading to unexpectedly wide distributions and non-intuitive responses to environmental covariates such as annual temperature range.

Uncertain or unexpected effects of covariables in species abundance can be influenced by two main factors. (1) ISDMs often rely on coarse-resolution environmental data that overlook critical microclimatic conditions shaping species distributions. Microclimates can vary substantially over very short distances due to physical features such as topography, vegetation structure, and soil composition (Potter et al., 2013). As a result, species dependent on these localized conditions may be poorly represented in broad-scale models based on averaged climatic data, potentially leading to inaccurate predictions of their range. Due to the coarse resolution of CHELSA (1 km), which does not consider microclimatic conditions within the canutos, its resolution may obscure canyon-slope temperature differences. In the future, adjustments based on data from portable micrometeorological stations could be incorporated. (2) Local abundance is determined by a variety of fine-scale ecological factors in addition to the broad environmental gradients shaping species’ distributions (MacArthur et al., 1972; Peterson et al., 2011; Staniczenko et al., 2017; Mertes and Jetz, 2018). These include prevailing disturbance regimes and successional stages (Morris et al., 2019), biotic interactions (Dallas and Hastings, 2018), dispersal barriers causing isolation (Reif et al., 2006), and environmental and demographic stochasticity (Lande et al., 2003). Since correlative models such as ISDMs do not incorporate these fine-scale ecological processes and stochastic factors, species local abundance cannot be only modeled via coarse-scale factors. Consistent with this, Sporbert et al. (2020) reported no clear correlation between coarse-scale climatic suitability predicted by species distribution models and observed local species abundance.

The results revealed that spatiotemporal random effects had a greater influence than fixed effects in explaining the underlying intensity of species distribution. This indicates that, even after accounting for the main environmental covariates through fixed effects, a substantial amount variation persists at the local level. Such unexplained variability is captured by the random effects, highlighting the importance of local-scale heterogeneity. Model M21, in contrast to M13, successfully estimates a joint spatiotemporal structure shared by the three species, taking advantage of ISDMs’ ability to model multiple species simultaneously. In M13, however, the species-specific components appear to be overfitted, resulting in the joint random effect absorbing residual variation that does not truly reflect shared spatial patterns among the species

### 4.1 Limitations and future research directions

While the present study has evaluated the benefits of ISDMs and data fusion and the spatio-temporal status for three endangered fern species, there are limitations deserving further studies:

1. A significant limitation of this study concerns the use of linear extrapolation to project bioclimatic variables beyond the observational period. Our analysis employed only 6 years of observed climate data to establish trends, which is substantially shorter than the 10–30 years typically recommended for robust climate trend detection. We acknowledge that such short time periods carry substantial uncertainty and may not represent ongoing climate trajectories. However, this temporal scope was necessitated by data availability constraints: the observational period aligns with the occurrence of species abundance surveys from the Andalusian Fern Recovery Plan, ensuring consistency between climate predictors and species response data. This temporal alignment is methodologically critical because predictor-response mismatches would introduce substantially greater uncertainty than the 6-year observational window itself.

To assess sensitivity to this methodological choice, we evaluated three distinct extrapolation approaches for the 2019–2023 projection period (**Supplementary Figure 23**): (1) repeating climatological patterns (last observed year), (2) applying long-term climatological means (2014–2019 average values), and (3) linear extrapolation of observed trends. All three approaches yielded similar central tendencies and comparable uncertainty ranges across the study region, suggesting that results are relatively robust to the choice of extrapolation method despite the short temporal window. Nevertheless, we recognize that this temporal limitation warrants careful interpretation. Future studies should ideally incorporate sensitivity analyses across longer observational periods and consider incorporating meteorological station data to enhance the robustness of climate projections.

2. Although we hypothesized that spatial redundancy between GBIF observations and structured abundance data from the Andalusian Fern Recovery Plan would limit the predictive performance of data fusion approaches, a preliminary evaluation of spatial overlap revealed findings warranting further investigation. Our analysis revealed minimal spatial redundancy at proximal distance (1.92% at 100 m, 3.23% at 500 m globally), indicating that GBIF records provided predominantly spatially independent information, i.e. non-replicated locations of populations and GBIF occurrences. Across species, mean spatial redundancy was minimal: 1.92% (95% CI: 0–12.5%) at 100 m and 3.23% (95% CI: 0–18.8%) at 500 m. *C. macrocarpa* showed the highest median overlap (6.25% at 100–500 m, 95% CI: 6.25–18.8%), while *D. caudatum* and *P. incompleta* exhibited negligible redundancy below 500 m (upper 95% CI limits: 3.75% and 11.1%, respectively). These findings demonstrate that >96% of GBIF records provided spatially independent information, with minimal overlap between GBIF presence locations and structured monitoring data.

However, spatial redundancy increased substantially when evaluated using buffer radii corresponding to the posterior median spatial range estimated from our SPDE-AR for M21 for each species: 2,800 m for *C. macrocarpa*, 6,700 m for *D. caudatum*, and 3,900 m for *P. incompleta*. *C. macrocarpa* exhibited 40.6% overlap (95% CI: 6.25–62.5%), *D. caudatum* showed 62.5% overlap (95% CI: 0–100%), and *P. incompleta* demonstrated 33.3% overlap (95% CI: 16.4–50.3%) (**Supplementary Figure 24**). These results reveal a critical distinction: while GBIF records were spatially independent at short distances, a substantial proportion fell within the effective spatial correlation range of our models. This may explain the marginal contribution of GBIF data to model performance despite their separation from monitoring locations at conventional distances, due to information already captured by the model.

Future work should explore how model performance incorporating data fusion varies as a function of the cumulative distribution of spatial overlap across buffer radii of increasing size, employing virtual species distributions. Simultaneously, it would be valuable to evaluate the effects of weighted likelihood functions when fitting ISDMs weighted by data source. Unweighted approaches may be dominated by the larger dataset because the joint log-likelihood function is additive. These analyses would provide evidence-based guidelines for determining when data fusion is appropriate, whether weighted ISDMs should be employed, and under which conditions they should be preferred, thereby contributing to improved ISDM frameworks for studying species populations and their distributions.

## 5 Conclusion

Species’ abundance across their geographic range has recently been proposed as one of the essential biodiversity variables, particularly relevant for rare and threatened species, as it provides key insights into population status and conservation needs. In this study, we assessed the capacity of Integrated Species Distribution Models (ISDMs) to model the spatiotemporal abundance intensities of three threatened paleomediterranean relict ferns, *Culcita macrocarpa*, *Diplazium caudatum*, and *Pteris incompleta*, by jointly modeling multiple species and integrating both structured count data and opportunistic citizen science records. Our findings show that joint species modeling improves abundance predictions; however, the inclusion of opportunistic data in ISDMs did not enhance, and in some cases reduced, temporal interpolation performance. Log-intensity trends over the last decade indicate general stability, with localized increases in some populations and declines estimated only for two populations of *C. macrocarpa*. Given the specific assumptions under which ISDMs are most effective, we recommend future research to explore the incorporation of co-occurring phytosociological species and microclimatic variables, which, enabled by advances in technologies such as LiDAR, could improve fine-scale predictions and offer a more comprehensive understanding of ferns ecological responses and distributional dynamics.

## Data Availability

The original contributions presented in the study are included in the article/**Supplementary Material**. Further inquiries can be directed to the corresponding author.
