# Supplementary material for: Data fusion and integrated species distribution models for three endangered ferns (Culcita macrocarpa, Diplazium caudatum, and Pteris incompleta) in a Mediterranean biodiversity hotspot
Source: Front Plant Sci. 2025 Dec 2;16:1650159. doi: 10.3389/fpls.2025.1650159 (PMC12705598; doi:10.3389/fpls.2025.1650159)
Supplement: Supplementary file 1 [file Table1.docx]

Supplementary Material

*Glossary*

**Credible Interval (CI).** A X% credible interval is a range of values containing the true parameter value with X% probability, based on its posterior distribution. Unlike frequentist confidence intervals, credible intervals allow direct probabilistic statements about the parameter: there is an X% probability that the parameter lies within the computed interval, given the observed data and specified model.

**Data generating process**. The underlying probabilistic mechanism that describes how observations are generated from true ecological processes (latent state model or latent structures).

**First-order autoregressive model (AR(1)).** A first-order autoregressive (AR(1)) process is a time series in which each observation depends linearly on its immediately preceding value plus a random noise term. In hierarchical or mixed-effects models, an AR(1) structure can be specified for random temporal effects to capture persistent correlation over successive time points.

**Gaussian Fields (GF).** Random fields where the constituent variables follow a Gaussian probability density function, where any finite collection of field variables follows a multivariate Gaussian distribution. A random field constitutes the representation of the joint probability distribution for a set of random variables. In spatial statistics, the relationship between spatial dimensions is modeled through a covariance structure that completely defines the field's behavior. The Gaussian field typically has zero mean and a spatially structured covariance matrix defined, which model the degree of spatial correlation between positions. The covariance matrix is commonly defined using the Matérn covariance function.

**Gaussian Markov Random Fields (GMRF)**. Probabilistic graphical models widely used in spatial statistics and related fields to model dependencies over spatial structures. GMRFs constitute the computationally efficient approximation of a Gaussian Field (GF), where the fundamental difference lies in that GMRFs possess a sparse precision (inverse covariance) matrix, while GFs have dense covariance matrices that are computationally expensive to manipulate. A GMRF is defined by the conditional Markov property, meaning that two variables are only conditionally dependent if they are neighbors in the associated graph. The SPDE (Stochastic Partial Differential Equation) approach enables explicit connection between GF and GMRF through finite element discretization of stochastic partial differential equations, obtaining sparse representations that reduce computational complexity in two-dimensional spatial problems

**Hierarchical Bayesian Models**. A class of Bayesian statistical models that enable structuring the modeling process across multiple interconnected hierarchical levels, facilitating complete uncertainty propagation throughout all model levels. This hierarchical structure reflects the natural organization inherent in ecological data and the processes that generate them. The typical architecture of these models comprises the following components organized hierarchically: hyperpriors, prior distributions, latent process, data generating process, observational model.

**Highest Posterior Density Interval (HPDI)**. HPDI can be defined as the narrowest Credible Interval (i.e. based on a probability distribution, it represents the possible range of values for a parameter given a specified probability mass) that contains the specified probability mass, meaning that all points within this interval have a higher probability density than any points outside the interval. For approximately symmetric distributions, the HPDI closely matches the equal-tailed credible interval.

**Hyperpriors**. Probability distributions that govern the parameters of prior distributions, allowing information to propagate from higher to lower levels of the model.

**Information Sharing via Partial Pooling**. A fundamental estimation mechanism in Bayesian hierarchical models where group-specific or observation-specific random effects "borrow statistical strength" from a common population distribution. This generating distribution, characterized by hyperparameters for central tendency and variability, exerts a shrinkage effect that pulls individual estimates toward the population mean. The magnitude of this shrinkage depends inversely on group sample size and available information: groups with less data experience greater shrinkage toward the population mean, while groups with more information retain estimates closer to their observed values, thus optimizing the bias-variance trade-off in estimation.

**Integrated Species Distribution Models (Joint Likelihood)**. A specific type of Bayesian Hierarchical Models that, when fitted in R-INLA, also constitute Latent Gaussian Models, with multivariate response variables. These models enable the formal integration of multiple ecological "data currencies" (presences, abundances, densities, biomass, etc.) that can originate from multiple species. Their structure is based on sub-models that link an unobserved latent state, representing the true distribution of a species, with one or more observation models that describe how the observed data were generated from this latent state. While observation models are specific to each dataset, the latent state and its defining parameters are shared across all data sources through a joint likelihood formulation, thus enabling simultaneous inference on the underlying distribution of multiple species using multiples data types.

**Joint likelihood.** A fundamental component of Bayes' theorem, necessary for obtaining the posterior distribution of model parameters. The likelihood function represents the probability of observed data as a function of model parameters, treating the data as fixed and parameters as variable. Within the framework of Integrated Species Distribution Models (ISDM), the joint likelihood is defined as the product of individual likelihoods for each data currency and/or species data, conditional on their corresponding latent distribution. This formulation enables the formal integration of multiple heterogeneous information sources under the assumption of conditional independence, where each component contributes proportionally to the global inference about shared model parameters.

**Latent Gaussian Models (LGM)**. A particular class of Bayesian Hierarchical Models characterized by an additive structure in the linear predictor and an observation process conditional solely on this predictor and parameters specific to the chosen likelihood. They are organized in three hierarchical layers: 1) the observation model, where observations yi are conditionally independent given a specified exponential family distribution; 2) the latent Gaussian field η, which follows a multivariate Gaussian distribution with specific covariance structure; and 3) the hyperparameters θ that govern the structure of the latent field. The linear predictor takes the form η = β₀ + Σβⱼxⱼ + Σf⁽ˡ⁾(uₗ), where β₀ is the intercept, βⱼ are linear effects, and f⁽ˡ⁾(·) represent smooth functions of covariates that can include nonlinear, spatial, or temporal effects. This class of models can accommodate everything from generalized linear models to complex mixed models with spatio-temporal dependence

**Latent structures**. Unobserved variables that can be subdivided into multiple levels according to the inherent hierarchy in the data, capturing unexplained variability and spatial or temporal heterogeneity of the system.

**Link function.** A link function defines how the linear predictor in a model relates to the expected value of the response variable’s probability distribution. It transforms the linear predictorso that it maps onto the valid range of the distribution’s mean.

**Linear predictor.** In a Bayesian hierarchical model, the linear predictor is the additive sum of fixed and random effects that defines the latent state. This predictor is connected to the observation or state model via the chosen link function.

**Log-Gaussian Cox Process (LGCP)**. A spatial point process model obtained under the assumption of a log-Cox process, that is, a Poisson process with spatially varying intensity where the logarithm of this intensity is modeled via a Gaussian linear predictor. Formally, it is defined as Λ(s) = exp{Z(s)}, where Z(s) is a linear predictor acting as a latent field and Λ(s) represents the stochastic intensity function. The LGCP constitutes a natural extension of the homogeneous Poisson process that enables modeling of point patterns with structured spatial heterogeneity, where intensity variation is modeled through a linear predictor enabling the incorporation of fixed and random effects. Conditional on the intensity Λ(s), the process behaves as an inhomogeneous Poisson process.

**Log-score.** The log-score is a proper scoring rule for assessing probabilistic predictions by assigning a numerical score to each observed outcome based on its posterior distribution. A scoring rule is proper if, on average, it is optimized when the distribution matches the true data-generating distribution. The log-score is computed as the logarithm of the posterior predictive distribution evaluated at the observed value; higher scores indicate that the model assigns greater probability to the actual data, thus reflecting improved predictive performance.

**Observational model**. The observational model specifies how the underlying ecological process is linked to the data we actually collect, accounting for measurement error, imperfect detection, and sampling constraints. It describes the statistical relationship between true state variables and observed values. By explicitly modeling these uncertainties, the observational component ensures that inferences about the true ecological process correctly reflect both biological variation and the limitations of data collection.

**Penalized Complexity Priors (PC Priors)**. Prior distributions founded on Occam's razor, invoking the principle of parsimony whereby simpler model formulations should be preferred until there is sufficient evidence to support a more complex model. PC priors penalize deviations from a simple base model through a function that decays as a measure of increased complexity between the more flexible model and the base model increases. The complexity measure is based on the Kullback-Leibler divergence (KLD), which quantifies the distance between the flexible model distribution and the base model distribution. PC priors assign higher probability mass to the base (simpler) model, providing automatic regularization against overfitting, numerical stability in estimation, and enhanced interpretability of results by favoring more parsimonious explanations unless the data strongly support greater complexity. These priors are invariant to reparameterizations, have natural connections to Jeffreys' priors, and provide excellent robustness properties.

**Poisson Point Process**. A statistical description of the continuous spatial distribution of points that assumes Complete Spatial Randomness (CSR), also defined as a homogeneous spatial process. It is a point process whereby point events occur within a given study area in a completely random fashion, without interactions between points or variation in spatial intensity. This process is modeled using only one parameter: the intensity of the process, defined as the density of points within the specified area. Under CSR, points have equal probability of occurring at any location within the study area and their distribution follows a Poisson distribution with mean proportional to the area considered.

**Posterior Distribution**. A conditional probability distribution of a parameter that results from updating the prior probability with information summarized by the likelihood via an application of Bayes' theorem. Formally, it is expressed as P(θ|data) ∝ P(data|θ) × P(θ), where P(θ|data) is the posterior, P(data|θ) is the likelihood, and P(θ) is the prior distribution. The posterior distribution describes the epistemic uncertainty about statistical parameters conditional on a collection of observed data, representing everything that can be known about those parameters after integrating prior knowledge with empirical evidence.

**Posterior Predictive Distribution (PPD).** Inferences about unknown observables (e.g., new observations) are typically referred to as predictive inferences, as the distribution pertains to a quantity that is observable rather than latent. Given an observation yi , the PPD is the distribution of the values of yi generated by the model, conditional on the data already observed. The PPD can be understood as an average of the model's conditional predictions for new data, weighted by the posterior distribution of the model parameters, which allows for accounting for uncertainty in parameter estimation

**Prior distributions**. Prior beliefs about model parameters that incorporate previous knowledge or express uncertainty before observing the data.

**Separable Spatio-Temporal Random Effect.** A spatio-temporal model is separable when its joint covariance structure factors into independent spatial and temporal components. In practice, this means the spatio-temporal covariance matrix can be written as the Kronecker product of a purely spatial covariance matrix and a purely temporal covariance matrix, greatly simplifying computation and interpretation. In contrast, non-separable models feature covariance structures that cannot be decomposed in this way, requiring more complex formulations to capture space–time interactions.

**Shared Component Modeling**. The joint estimation of fixed and random effects that are shared across different data currencies and/or species within the framework of Integrated Species Distribution Models (ISDM). These shared components include environmental parameters, spatial structures (such as Gaussian fields), and temporal terms that are estimated using information from all entities (species/data types) for which the shared component is relevant. This approach enables statistical information exchange between heterogeneous data sources, improving estimation precision by leveraging underlying common patterns, while data-specific scaling parameters accommodate differences in observation processes and sampling efficiencies.

**Spatially Varying Coefficient Models (SVC)**. An extension of spatial random effects where regression coefficients of covariates vary smoothly and continuously across space. This spatial variation is typically modeled using Gaussian processes with Matérn covariance functions, which assume that coefficients exhibit greater similarity at nearby locations and that their variation follows structured spatial patterns, reflecting local heterogeneity in ecological relationships.

**State model.** In a Bayesian hierarchical model, the state model characterizes the hidden ecological process through unobserved latent variables. These variables are specified by linear predictors and linked to the observational model via suitable link functions. The state model encapsulates the true temporal or spatial evolution of the system.

**State-space Models**. Hierarchical models composed of two main sub-models: the observation process and the latent state process. The latent state represents the true species distribution and is modeled as a function of environmental covariates and spatiotemporal effects. The observation process statistically describes how the data were generated, conditional on the latent state. A fundamental characteristic of the state-space formulation is the assumption of conditional independence between observations given the latent state, meaning that observations are independent of each other when conditioned on their respective latent values or linear predictors. This structure allows separation of biological variability in the underlying process from measurement error inherent in the sampling process.

**Stochastic Partial Differential Equations (SPDE).** The SPDE approach provides an approximation of a continuously indexed Gaussian Field (GF) with Matérn covariance function by representing it as a discrete zero-mean Gaussian Markov Random Field (GMRF). The SPDE approach uses the Finite Element Method (FEM) to solve the SPDE in spatial modeling. This method partitions the study area D into a set of non-intersecting triangles using a Delaunay triangulation, resulting in a mesh consisting of N vertices. The mesh serves as the foundation for constructing N piecewise linear basis functions, that approximate the solution to the SPDE.

**Watanabe–Akaike Information Criterion (WAIC).** The WAIC is a fully Bayesian extension of the Akaike Information Criterion, offering a more rigorous alternative to the Deviance Information Criterion (DIC). It measures predictive accuracy by combining model fit with a complexity penalty based on the estimated effective number of parameters. WAIC also serves as a fast approximation to leave-one-out cross-validation, incorporating parameter uncertainty into its evaluation of predictive performance.

**Weakly Informative Priors**. Prior distributions that represent a compromise between objective (non-informative) and strongly informative priors based on expert knowledge. These priors are constructed by recognizing that while you usually do not have strong prior information about the value of a parameter, it is rare to be completely ignorant. Weakly informative priors are fundamentally designed to allow data to dominate the derivation of the posterior distribution, relegating prior information to a secondary regularizing role. This use of weak prior knowledge is often sufficient to regularize the extreme inferences that can be obtained using maximum likelihood estimation or completely non-informative priors, providing stability to the analysis without imposing overly strong constraints that might bias results toward predetermined values.


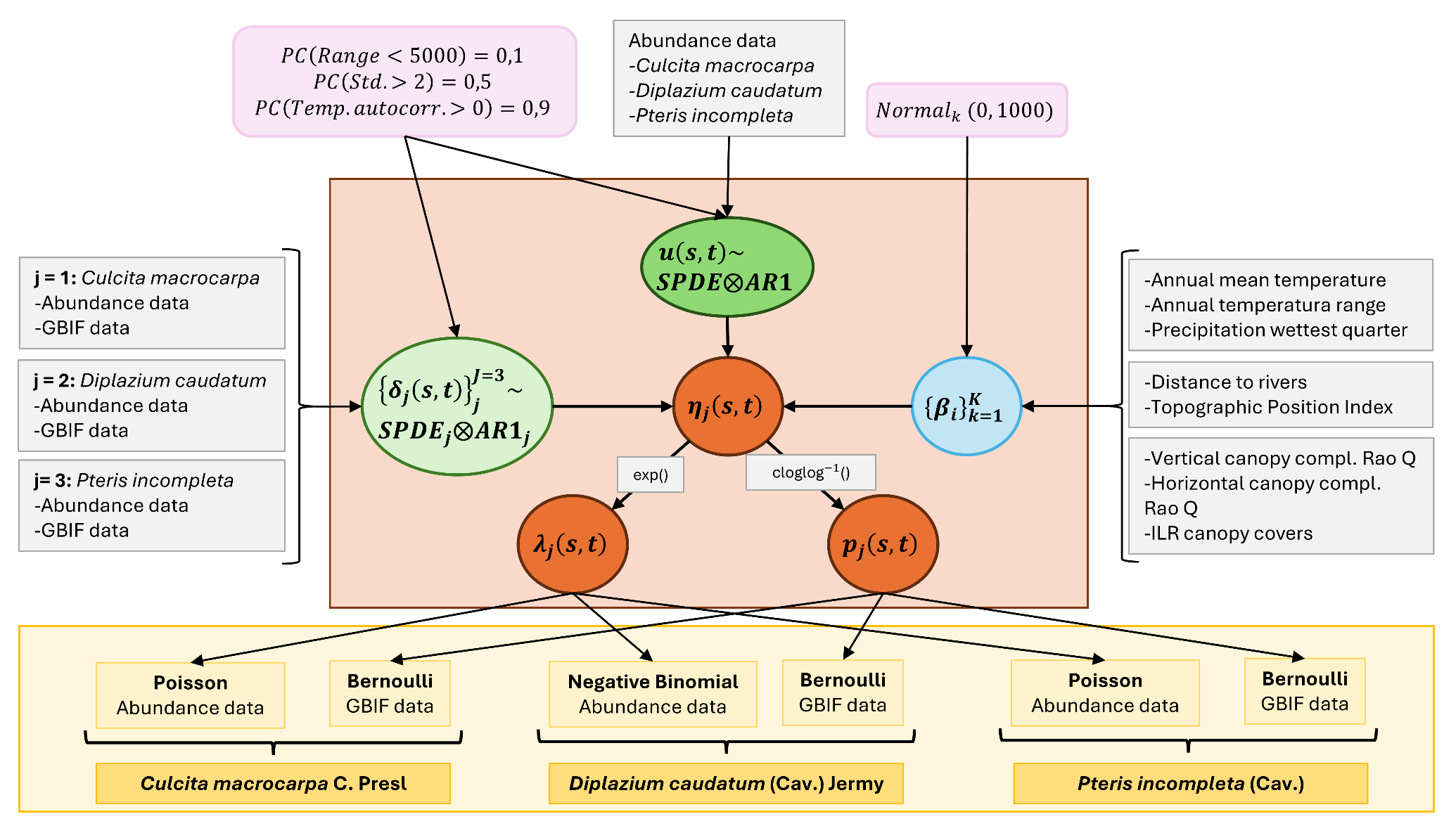


Supplementary Figure 1. Graphical representation of the structure of model M13. Elements belonging to the observation model are shown in yellow. The latent state model, the definition of the linear predictor, and the method for estimating its effects are shown in orange. Auxiliary information is represented in grey, while the specification of prior distributions is shown in purple.


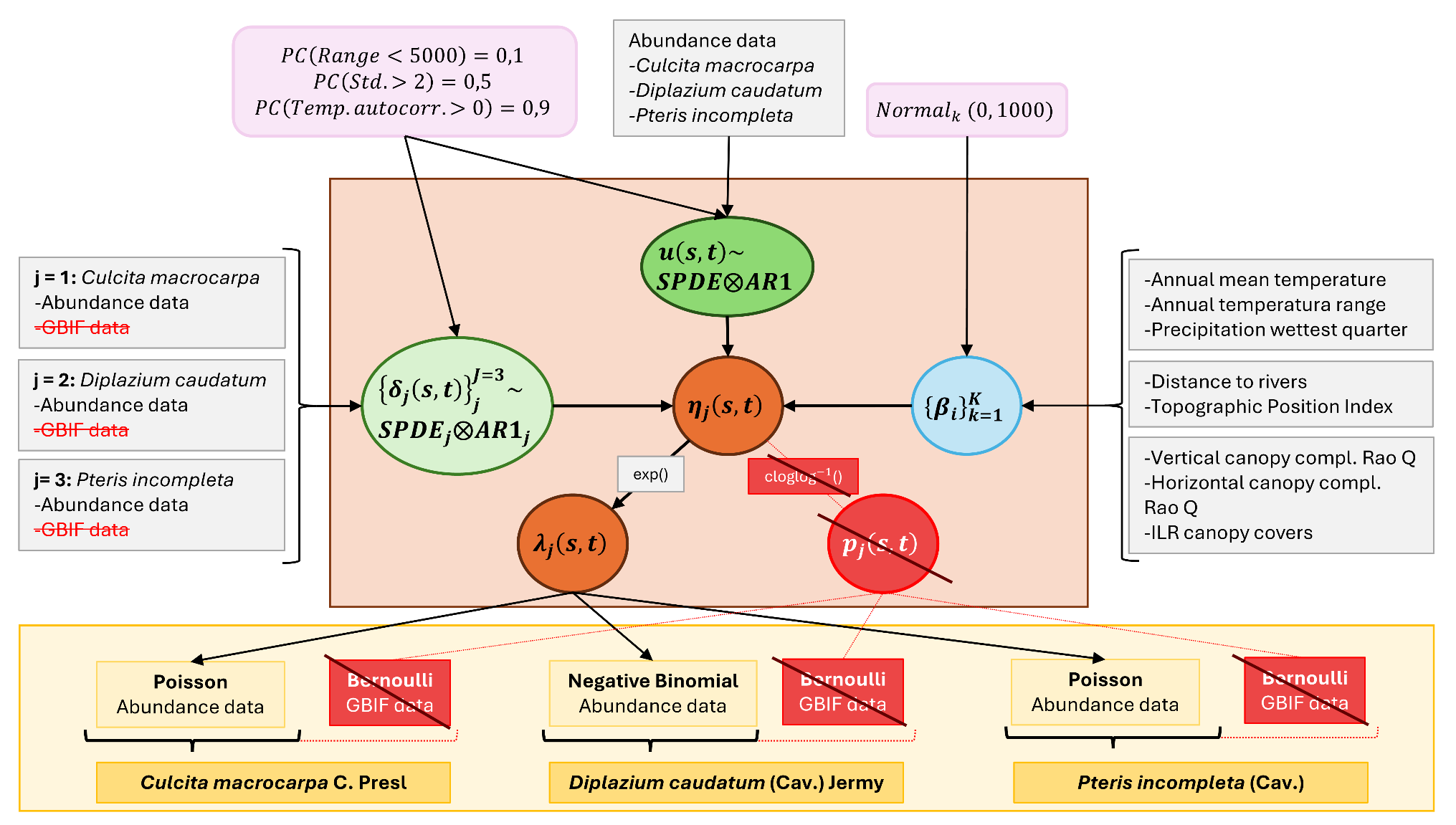


Supplementary Figure 2. Graphical representation of the structure of model M21. Elements belonging to the observation model are shown in yellow. The latent state model, the definition of the linear predictor, and the method for estimating its effects are shown in orange. Auxiliary information is represented in grey, while the specification of prior distributions is shown in purple. Components highlighted in red correspond to those specific to M13, as this model extends M21 by implementing data fusion, and are therefore not used in the fitting of M21
