# Supplementary material for: Data fusion and integrated species distribution models for three endangered ferns (Culcita macrocarpa, Diplazium caudatum, and Pteris incompleta) in a Mediterranean biodiversity hotspot
Source: Front Plant Sci. 2025 Dec 2;16:1650159. doi: 10.3389/fpls.2025.1650159 (PMC12705598; doi:10.3389/fpls.2025.1650159)
Supplement: Supplementary file 2 [file Table2.docx]

Supplementary Material


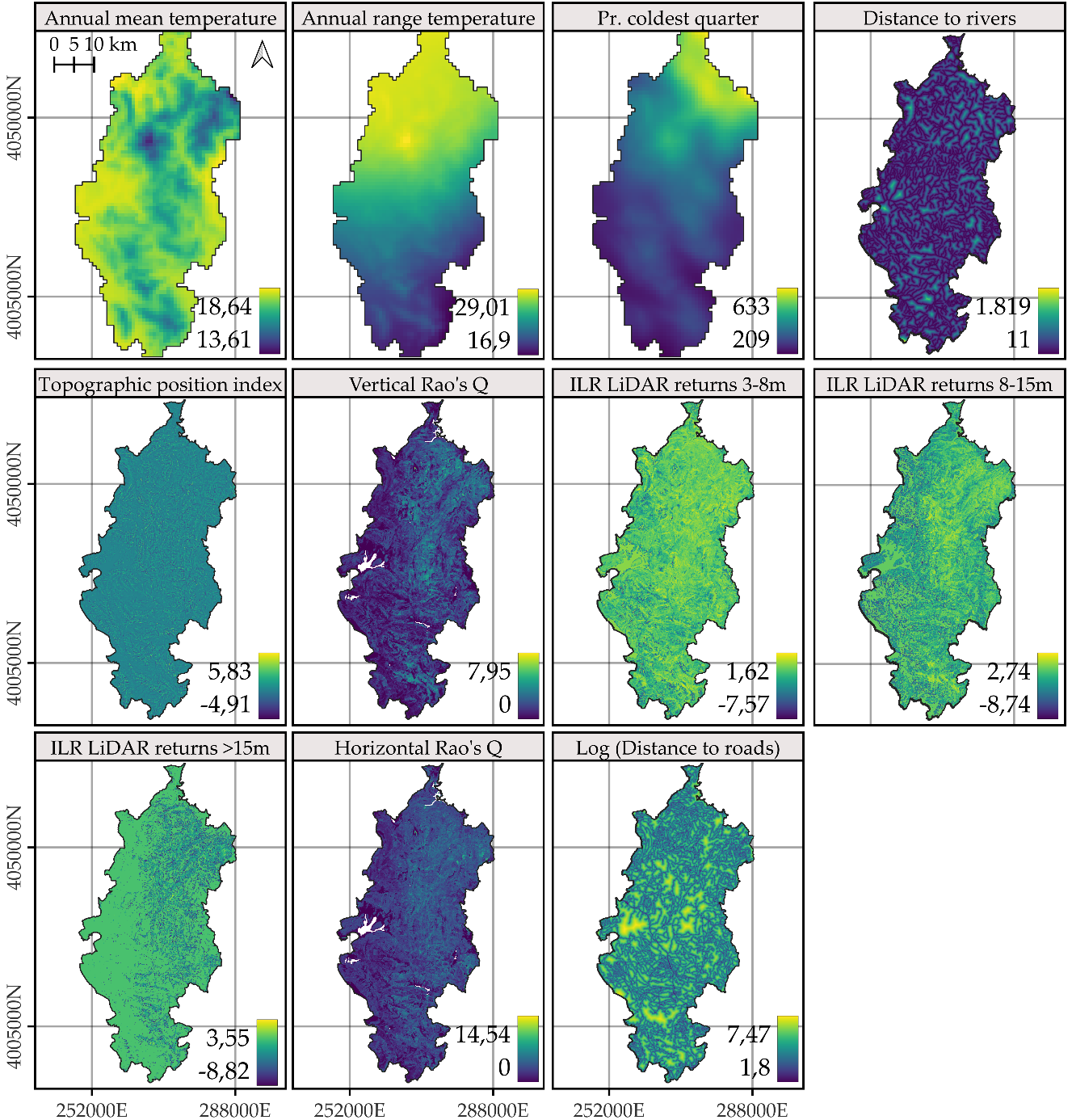


Supplementary Figure 1. Covariables used in the final model fitting.


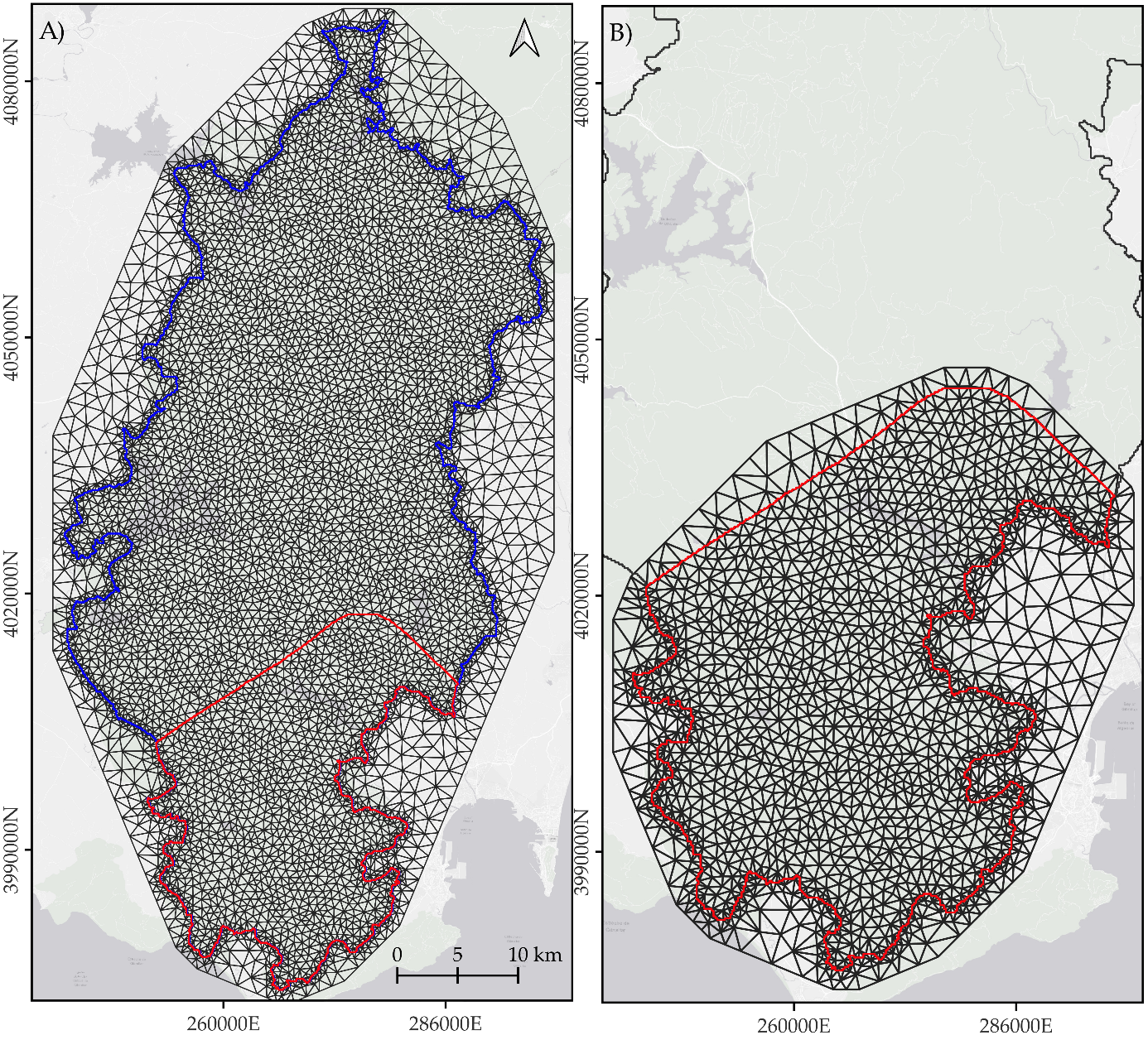
Supplementary Figure 2. Mesh generated using Delaunay triangulation for: (A) the entire Alcornocales Natural Park, and (B) the non-convex hull enclosing the observation points.


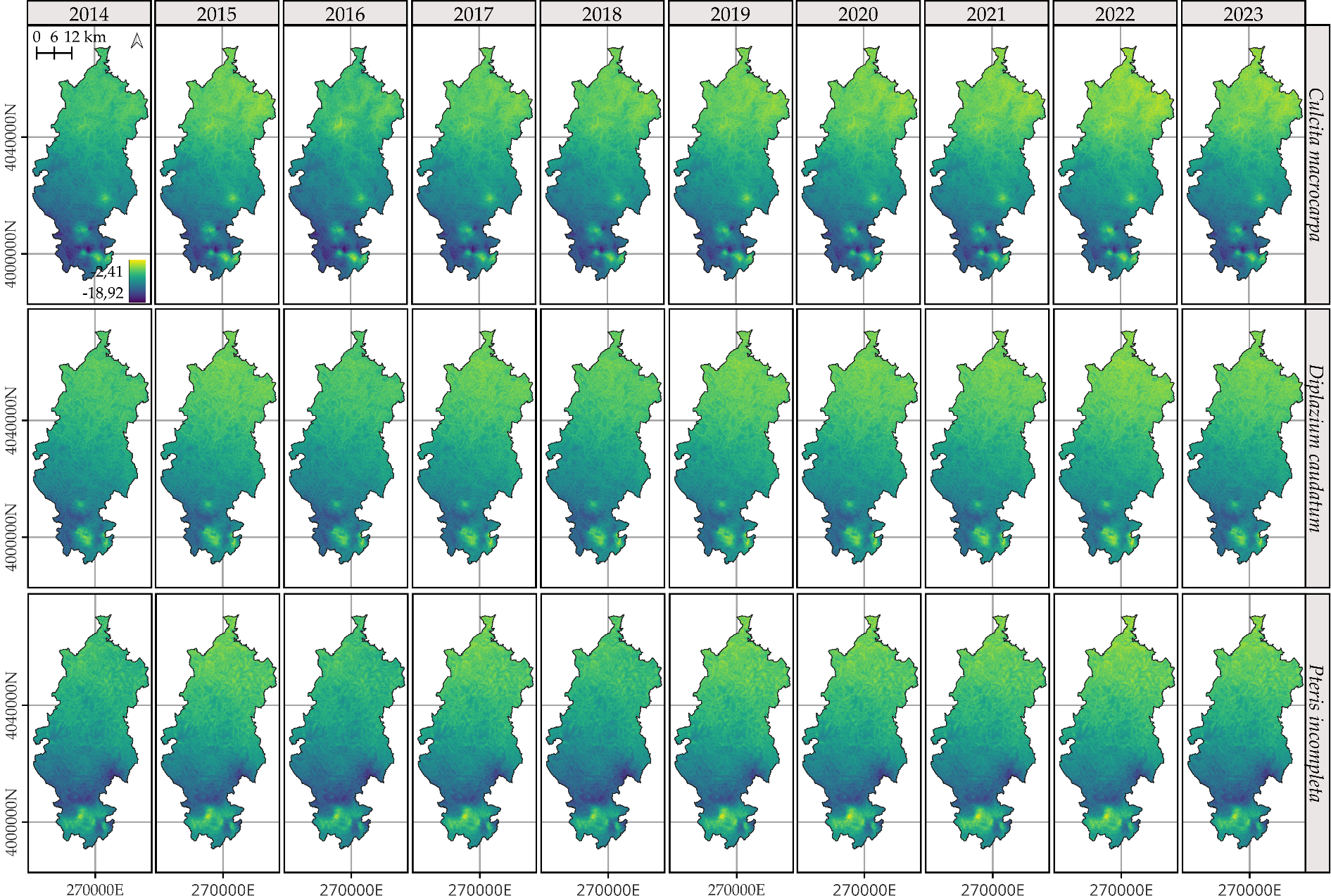


**Supplementary Figure 3.** *Posterior mean of the log-intensity for each species from 2014 to 2023, as estimated by model M13. A common legend is used across all maps.*

*
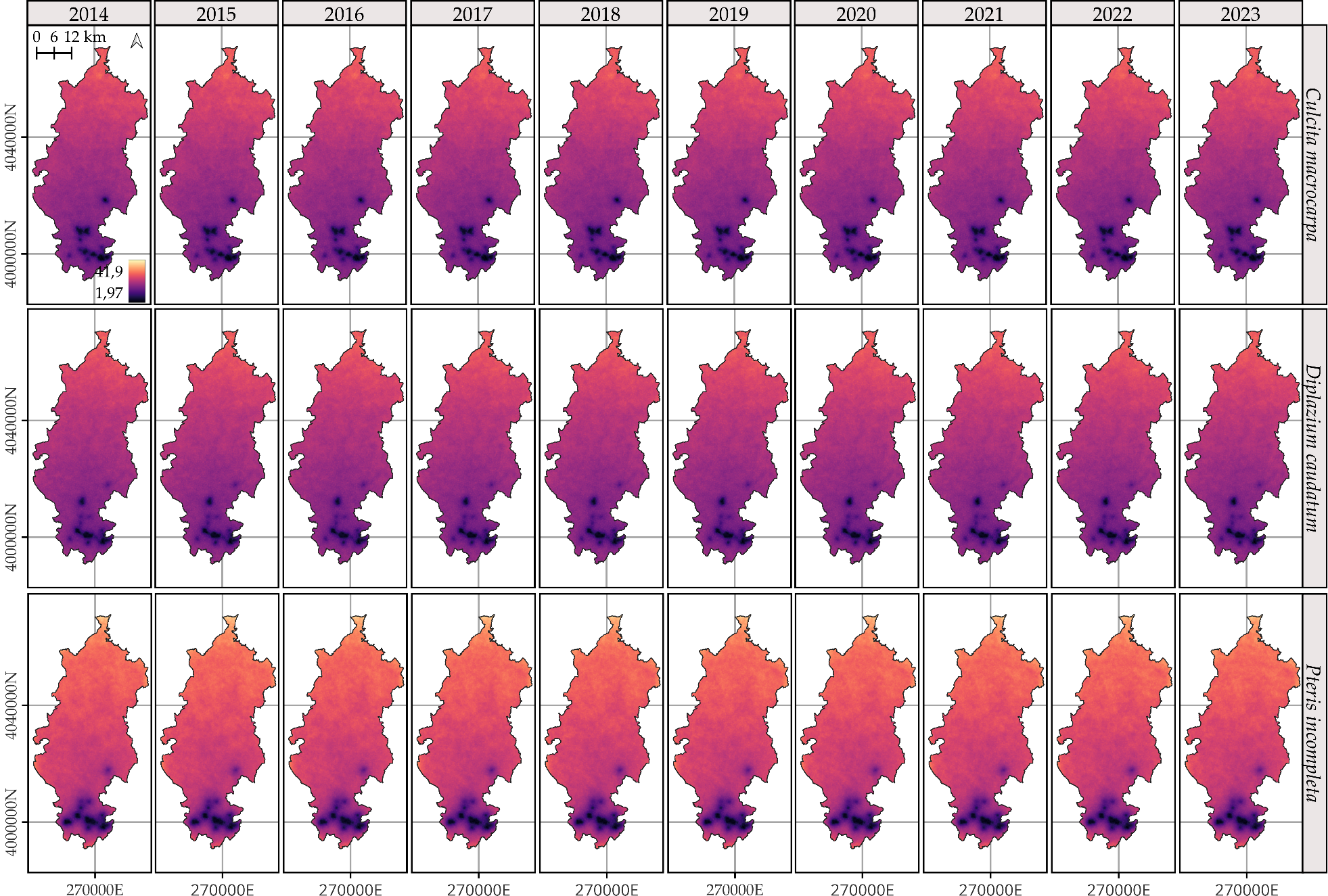
*

**Supplementary Figure 4.** *Posterior 95% coverage probability of the log-intensity for each species from 2014 to 2023, as estimated by model M13. A common legend is used across all maps.*


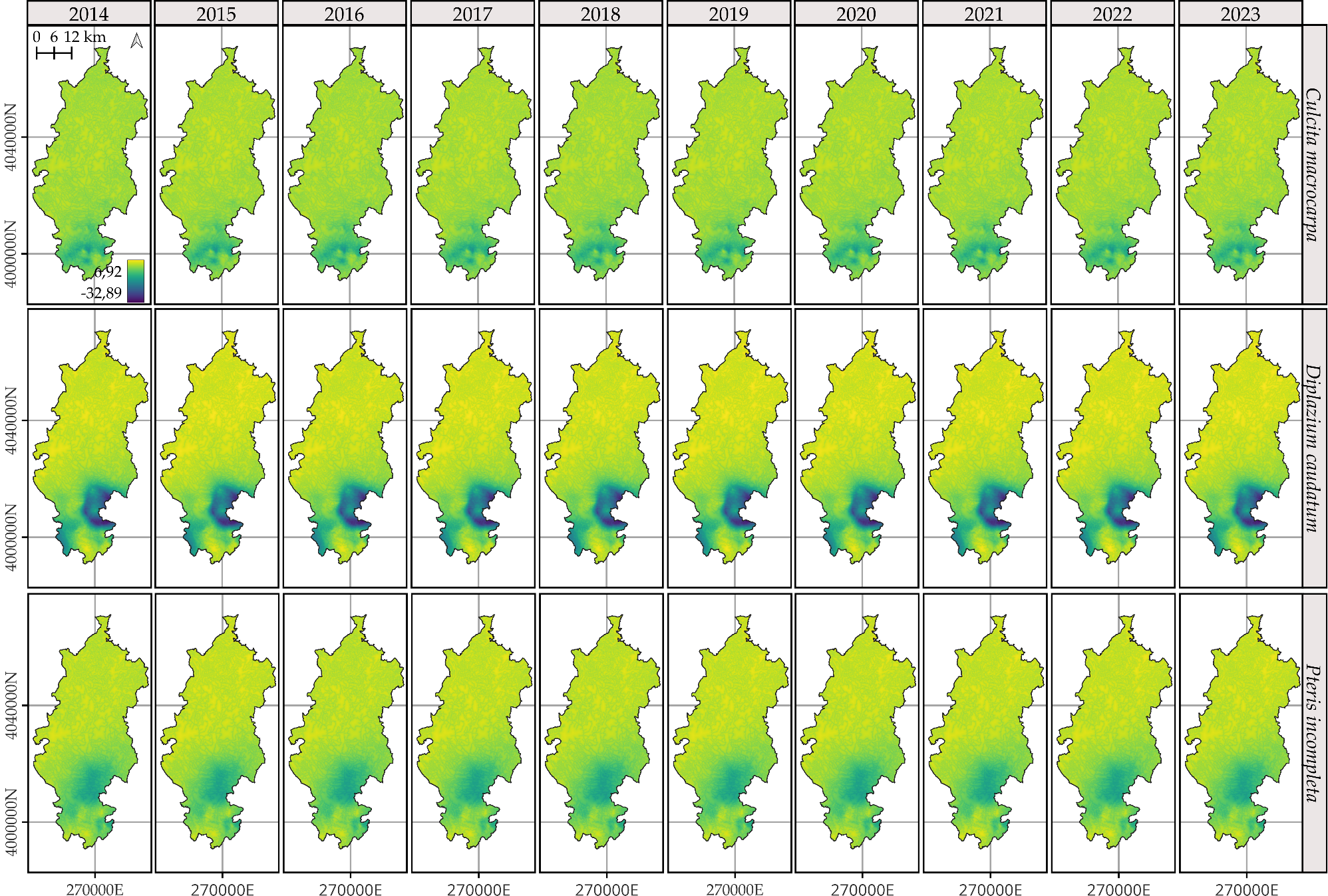


**Supplementary Figure 5.** *Posterior mean of the log-intensity for each species from 2014 to 2023, as estimated by model M16. A common legend is used across all maps.*


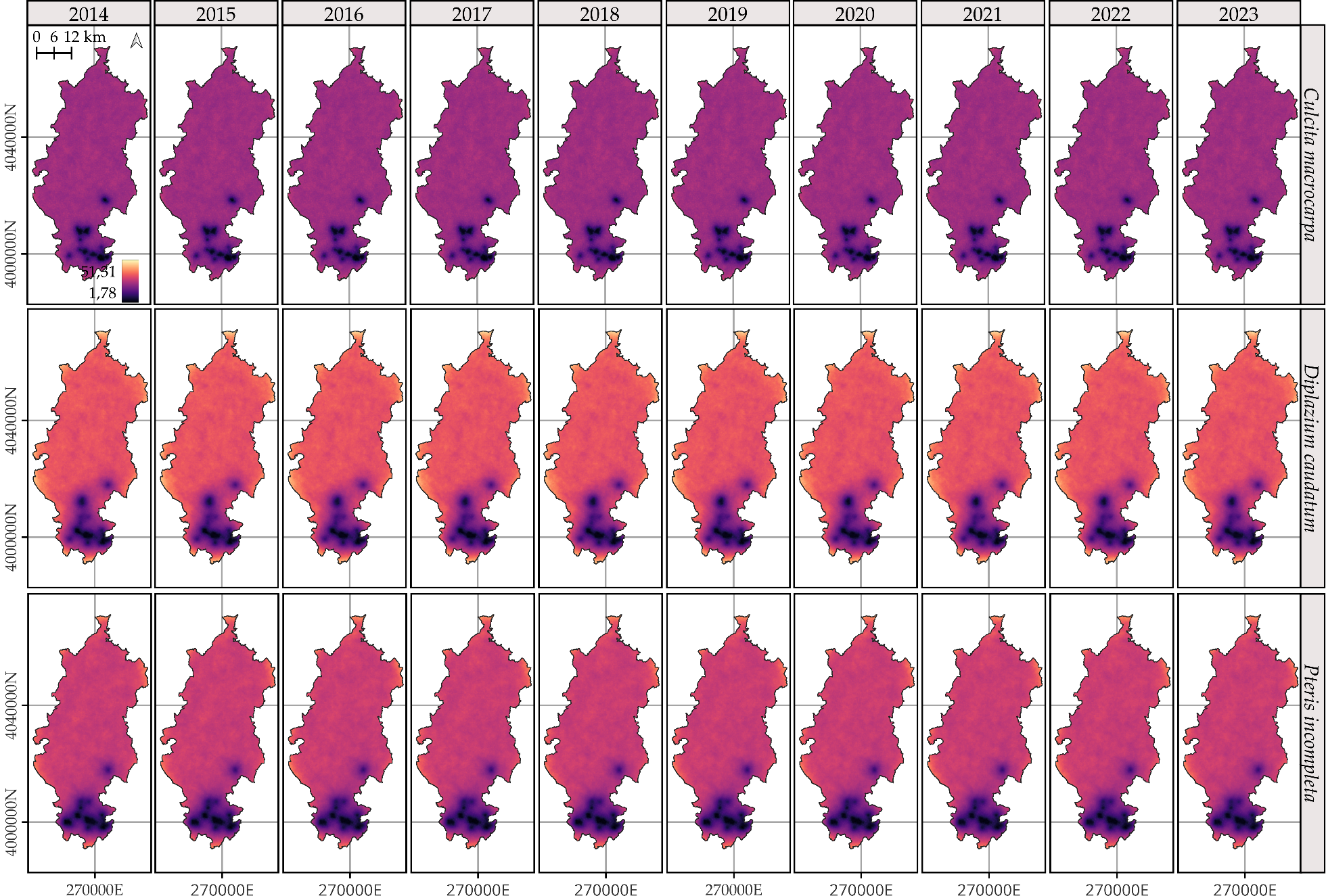


**Supplementary Figure 6.** *Posterior 95% coverage probability of the log-intensity for each species from 2014 to 2023, as estimated by model M16. A common legend is used across all maps.*


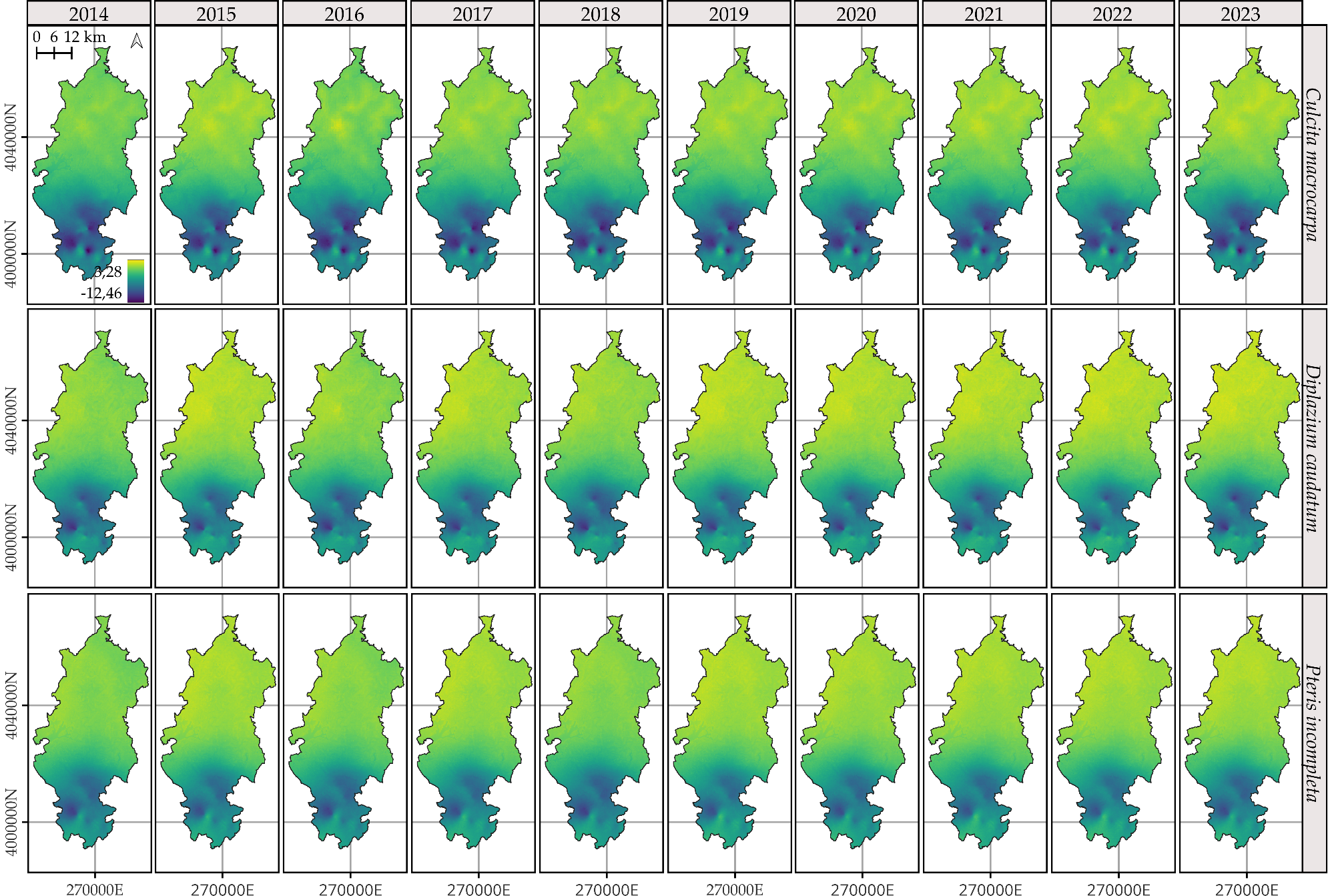


**Supplementary Figure 7.** *Posterior mean of the log-intensity for each species from 2014 to 2023, as estimated by model M21. A common legend is used across all maps.*


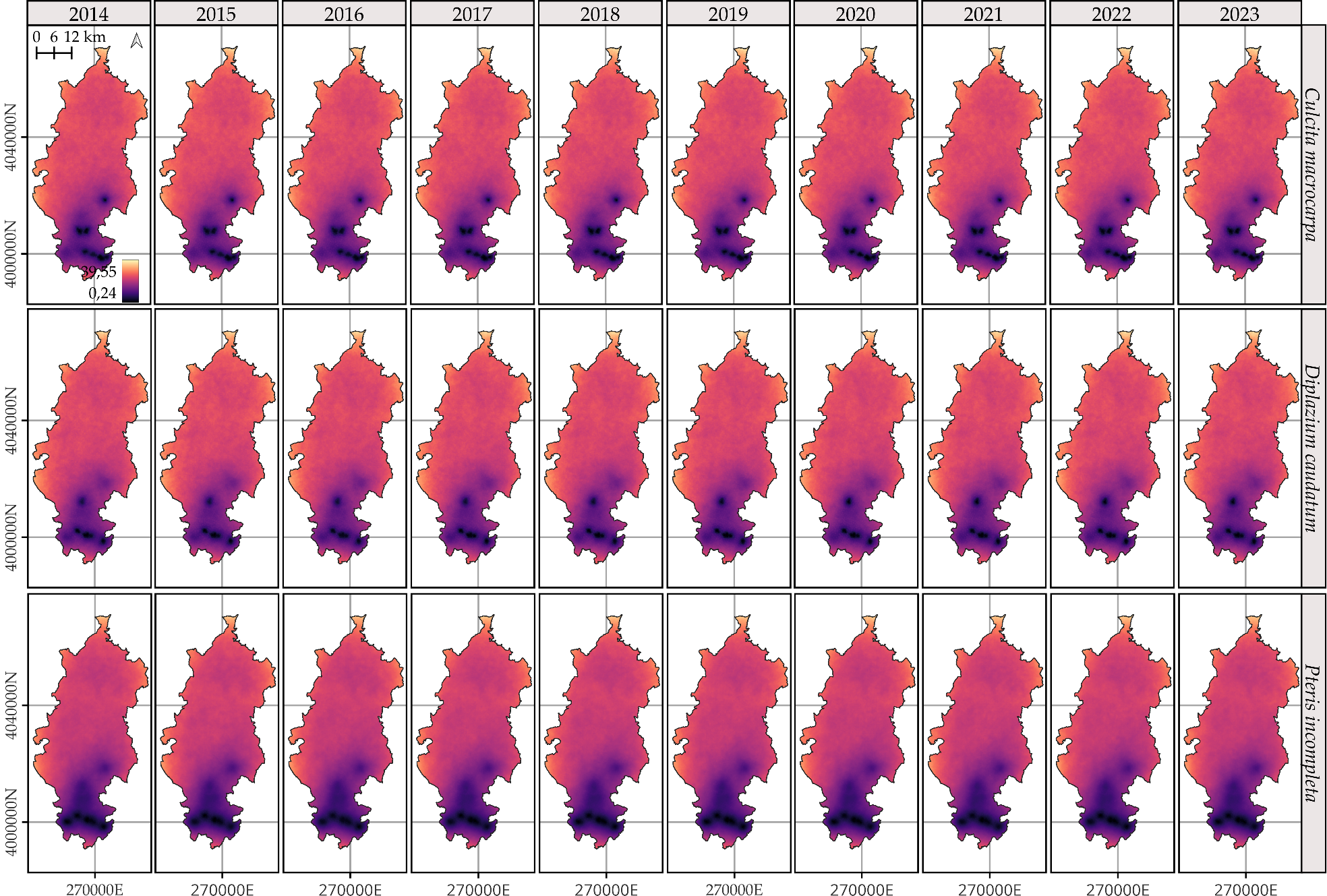


**Supplementary Figure 8.** *Posterior 95% coverage probability of the log-intensity for each species from 2014 to 2023, as estimated by model M21. A common legend is used across all maps.*

| **Table S1.** Comparison of fixed effect estimates across models M13, M16, and M21. CD refers to count data, used to estimate the linear predictor associated with species abundance. BD corresponds to Bernoulli data, used to model presence/absence based on GBIF records. | | | |
| --- | --- | --- | --- |
|  | M13 | M16 | M21 |
| Intercept CD Culcita | -0.366 [-1.923, 1.188] | -2.347 [-3.806, -0.890] | -0.398 [-1.894, 1.094] |
| Intercept CD Diplazium | 0.312 [-1.242, 1.861] | -0.484 [-2.073, 1.102] | -0.059 [-1.488, 1.367] |
| Intercept CD Pteris | -0.226 [-1.837, 1.382] | -1.166 [-2.730, 0.394] | 0.182 [-1.152, 1.514] |
| Intercept BD Culcita | 0.859 [-0.639, 2.353] | 1.077 [-0.434, 2.586] | — |
| Intercept BD Diplazium | -1.129 [-2.625, 0.363] | 0.101 [-1.496, 1.695] | — |
| Intercept BD Pteris | -0.003 [-1.584, 1.573] | 0.775 [-0.796, 2.343] | — |
| Annual mean temp. |  |  |  |
| Shared | — | -0.052 [-0.301, 0.196] | — |
| Culcita | -0.177 [-0.436, 0.081] | — | -0.105 [-0.352, 0.142] |
| Diplazium | -0.048 [-0.373, 0.277] | — | -0.094 [-0.388, 0.200] |
| Pteris | 0.069 [-0.175, 0.313] | — | 0.038 [-0.169, 0.245] |
| Annual range temp. |  |  |  |
| Shared | — | 0.135 [-0.128, 0.398] | — |
| Culcita | 0.236 [-0.089, 0.561] | — | 0.177 [-0.107, 0.461] |
| Diplazium | 0.164 [-0.162, 0.490] | — | 0.230 [-0.072, 0.531] |
| Pteris | 0.195 [-0.173, 0.562] | — | 0.092 [-0.233, 0.416] |
| Precipitation coldest quarter |  |  |  |
| Shared | — | -0.019 [-0.221, 0.183] | — |
| Culcita | -0.106 [-0.358, 0.146] | — | -0.059 [-0.273, 0.155] |
| Diplazium | -0.183 [-0.469, 0.102] | — | -0.248 [-0.512, 0.015] |
| Pteris | -0.240 [-0.495, 0.015] | — | -0.165 [-0.425, 0.095] |
| Dist. to rivers |  |  |  |
| Shared | — | 0.007 [-0.369, 0.383] | — |
| Culcita | 0.079 [-0.292, 0.449] | — | 0.036 [-0.318, 0.390] |
| Diplazium | -0.524 [-0.944, -0.105] | — | -0.412 [-0.769, -0.055] |
| Pteris | 0.468 [0.038, 0.898] | — | 0.001 [-0.302, 0.304] |
| Topographic Position Index |  |  |  |
| Shared | — | -0.068 [-0.132, -0.005] | — |
| Culcita | -0.080 [-0.142, -0.019] | — | -0.070 [-0.130, -0.011] |
| Diplazium | 0.066 [-0.018, 0.149] | — | 0.063 [-0.016, 0.141] |
| Pteris | -0.017 [-0.079, 0.045] | — | 0.015 [-0.035, 0.064] |
| Vertical Rao's Q |  |  |  |
| Shared | — | 0.048 [-0.137, 0.233] | — |
| Culcita | 0.029 [-0.150, 0.208] | — | 0.036 [-0.137, 0.210] |
| Diplazium | 0.018 [-0.083, 0.119] | — | 0.005 [-0.093, 0.103] |
| Pteris | 0.037 [-0.031, 0.105] | — | 0.049 [-0.012, 0.110] |
| ILR LiDAR retunrs 3-8 m |  |  |  |
| Shared | — | -0.120 [-0.223, -0.018] | — |
| Culcita | -0.107 [-0.206, -0.007] | — | -0.087 [-0.186, 0.012] |
| Diplazium | -0.112 [-0.233, 0.009] | — | -0.065 [-0.177, 0.047] |
| Pteris | -0.046 [-0.138, 0.046] | — | -0.109 [-0.188, -0.029] |
| ILR LiDAR retunrs 8-15 m |  |  |  |
| Shared | — | 0.006 [-0.160, 0.172] | — |
| Culcita | -0.007 [-0.167, 0.152] | — | -0.021 [-0.179, 0.136] |
| Diplazium | 0.113 [-0.089, 0.315] | — | -0.061 [-0.287, 0.164] |
| Pteris | -0.035 [-0.201, 0.130] | — | -0.078 [-0.216, 0.059] |
| ILR LiDAR retunrs >15 m |  |  |  |
| Shared | — | -0.001 [-0.053, 0.051] | — |
| Culcita | 0.004 [-0.048, 0.055] | — | 0.004 [-0.046, 0.055] |
| Diplazium | -0.220 [-0.428, -0.013] | — | -0.064 [-0.285, 0.156] |
| Pteris | -0.079 [-0.226, 0.068] | — | -0.023 [-0.147, 0.102] |
| Horizontal Rao's Q |  |  |  |
| Common estimate | — | -0.007 [-0.086, 0.072] | — |
| Culcita | -0.019 [-0.096, 0.058] | — | -0.009[-0.084, 0.066] |
| Diplazium | 0.058 [-0.047, 0.163] | — | 0.025 [-0.075, 0.125] |
| Pteris | 0.035 [-0.036, 0.105] | — | 0.008 [-0.054, 0.069] |
| Log (Dist. to roads) | -0.306 [-0.620, 0.007] | 0.803 [0.523, 1.082] | — |

**
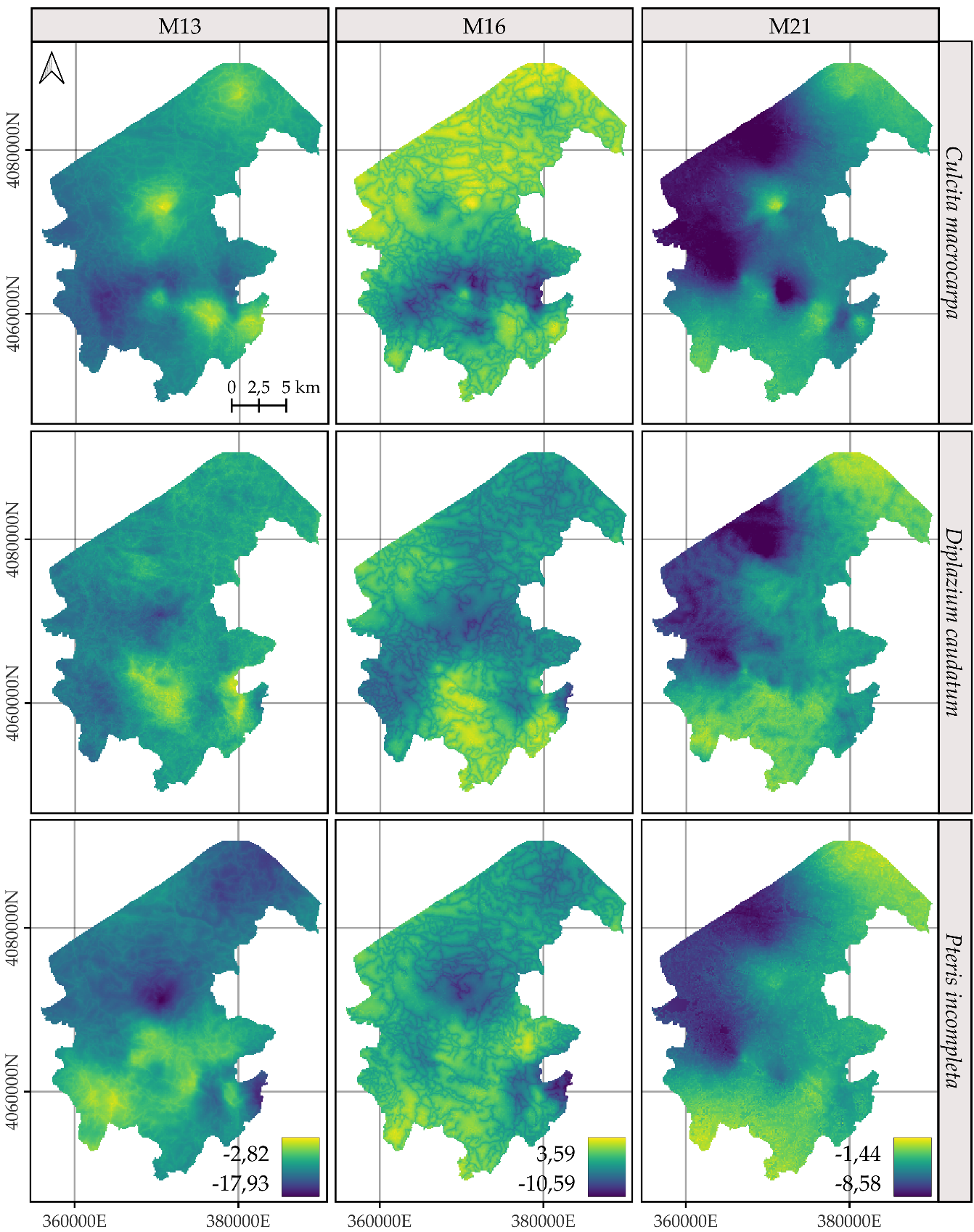
**

**Supplementary Figure 9.** Comparison of the log-intensity of the state equations across species for models M13, M16, and M21 in the year 2022. A common legend has been used across models to emphasize the comparison of spatial patterns among species under each modeling approach.

*
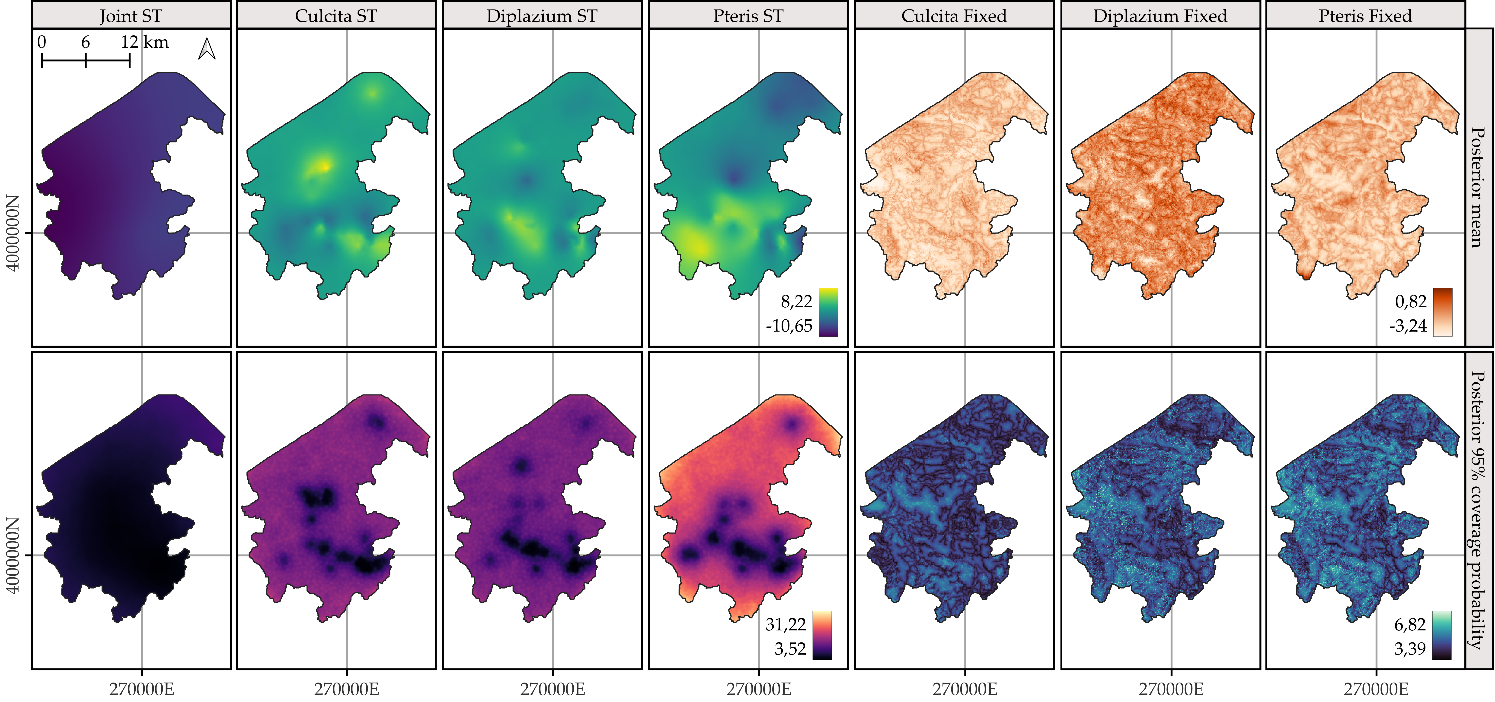
*

**Supplementary Figure 10.** Spatial marginal patterns associated with the spatio-temporal random effects and fixed effects in model M13. For each set of effects, both the posterior mean and the 95% coverage probability are shown. Common legends were used within each set of effects to allow for comparison of the magnitude or contribution of that component across species.

*
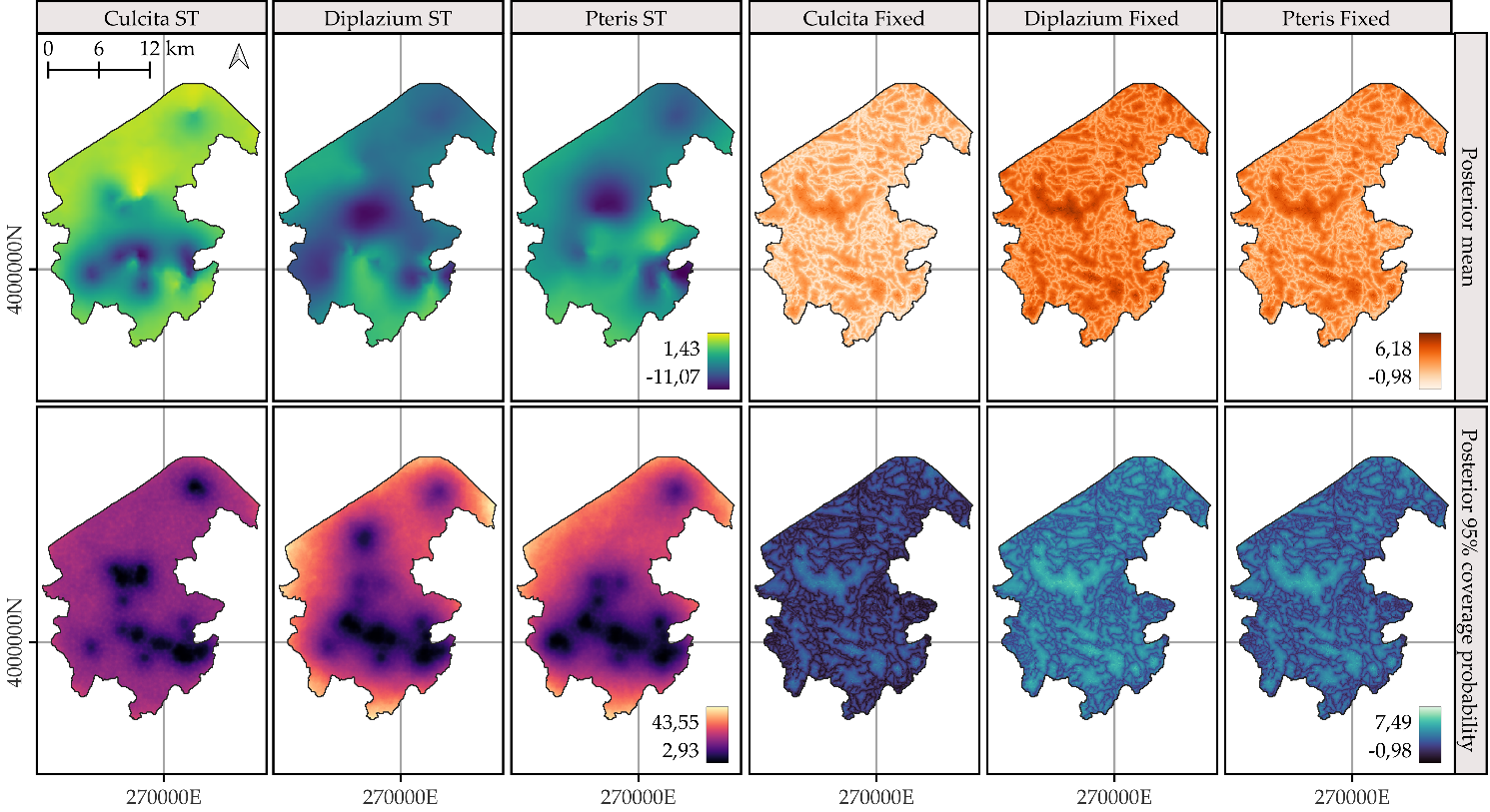
*

**Supplementary Figure 11.** Spatial marginal patterns associated with the spatio-temporal random effects and fixed effects in model M16. For each set of effects, both the posterior mean and the 95% coverage probability are shown. Common legends were used within each set of effects to allow for comparison of the magnitude or contribution of that component across species.


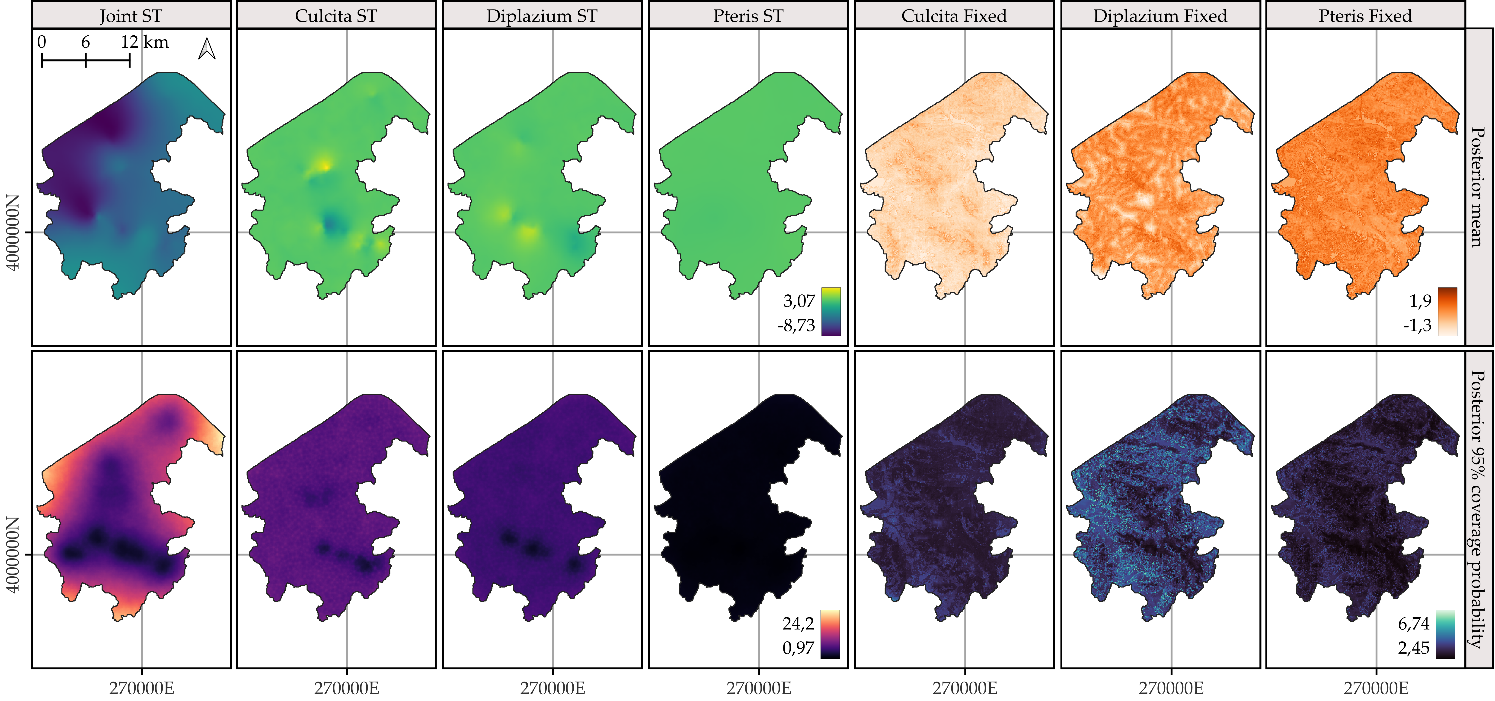


**Supplementary Figure 12.** Spatial marginal patterns associated with the spatio-temporal random effects and fixed effects in model M21. For each set of effects, both the posterior mean and the 95% coverage probability are shown. Common legends were used within each set of effects to allow for comparison of the magnitude or contribution of that component across species.

**
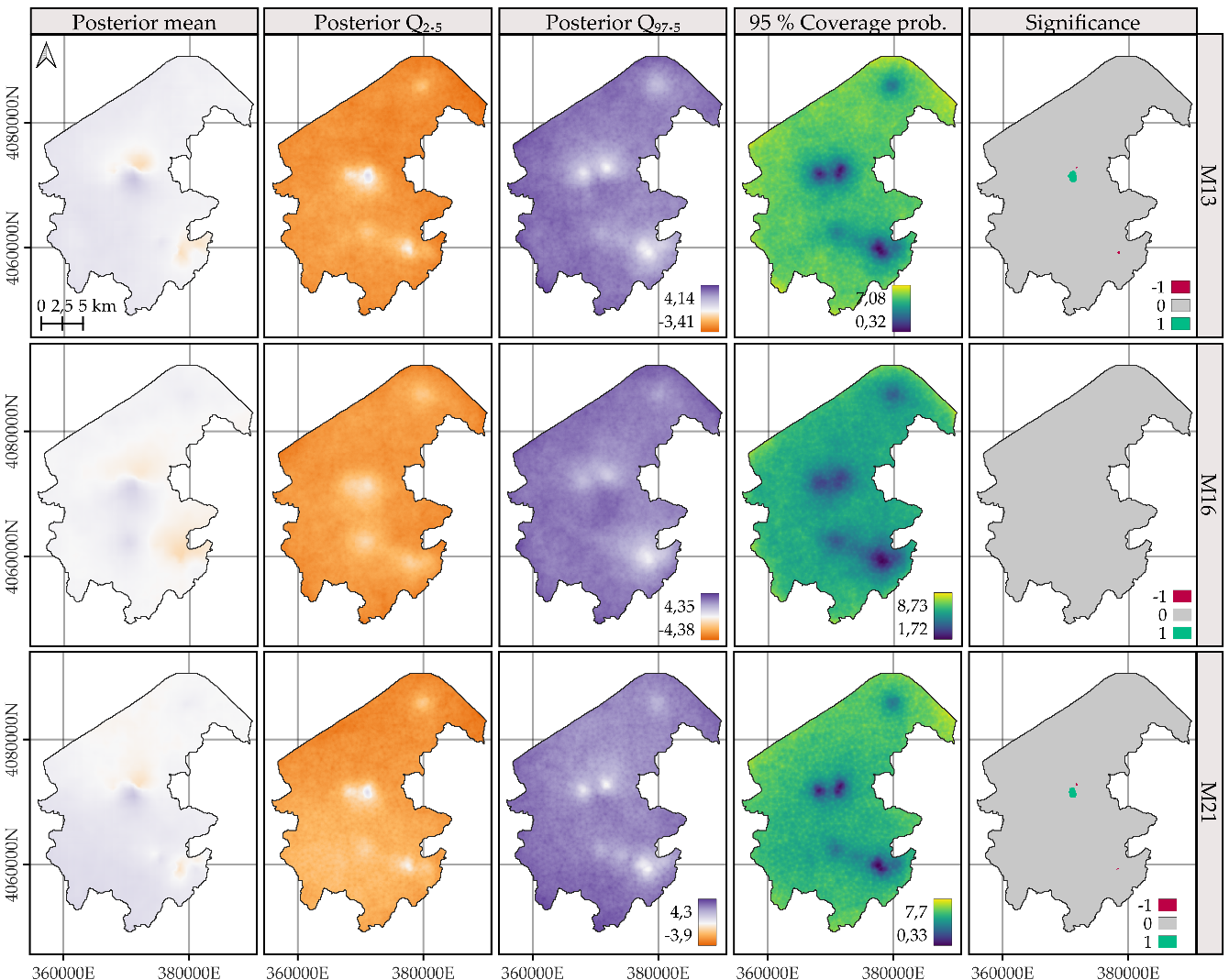
**

**Supplementary Figure 13.** Comparison of the net change in the log-intensity for *Culcita macrocarpa* between 2014 and 2023. Results are shown for each model based on the posterior distribution of the difference between 2023 and 2014. Positive values represent areas with an increase in log-intensity over this period, while negative values indicate a decrease. For each pixel, the posterior mean, the 2.5th and 97.5th percentiles, the 95% coverage probability interval, and the significance are reported. In a Bayesian context, significance is defined as those posterior distributions of log-intensity differences whose 95% coverage probability interval does not include zero. Accordingly, a value of -1 indicates a 95% probability of a decrease in intensity, a value of 1 indicates a 95% probability of an increase, and a value of 0 indicates that the 95% credible interval likely includes zero, suggesting no significant change over time in that area.

**
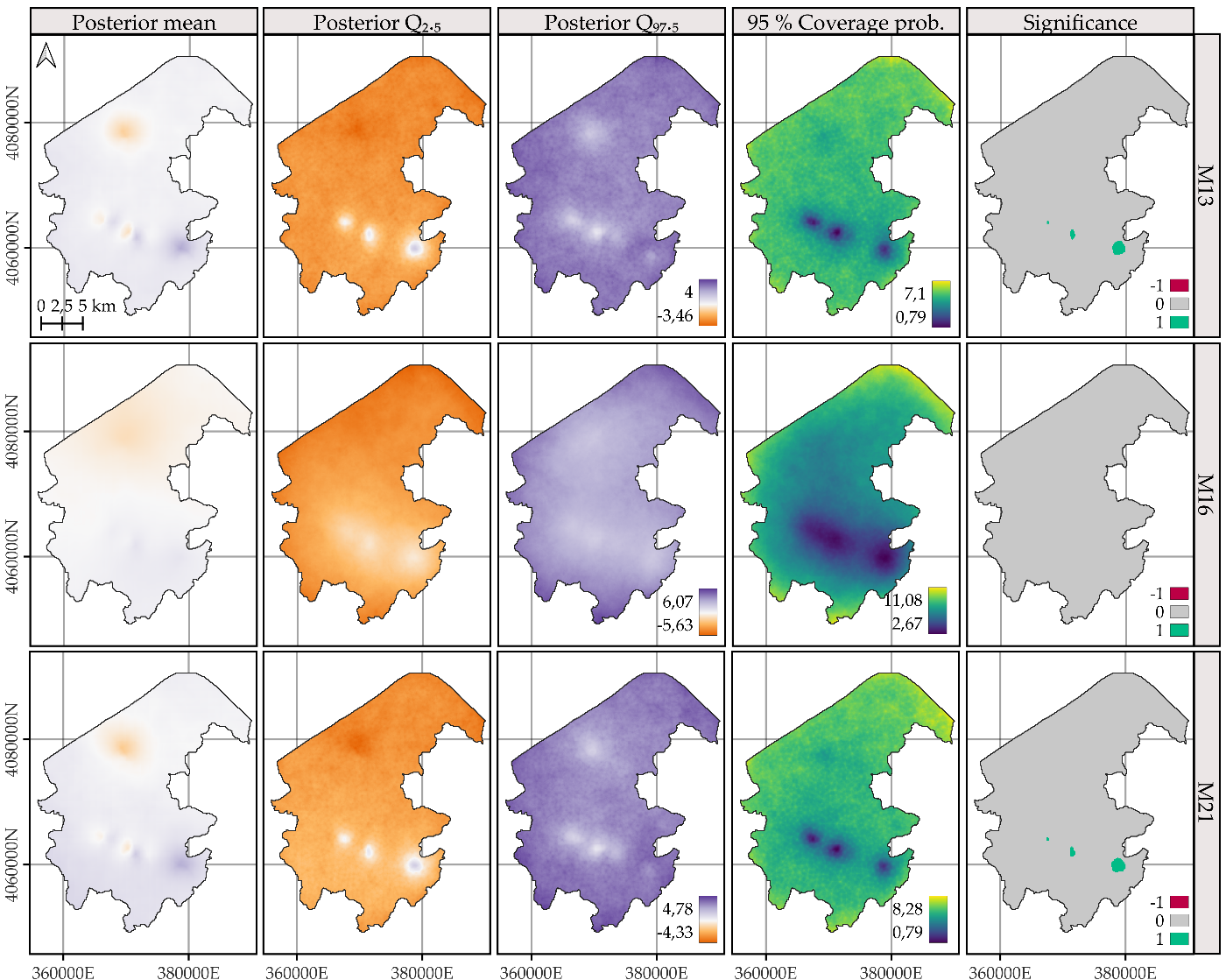
**

**Supplementary Figure 14.** Comparison of the net change in the log-intensity for *Diplazium caudatum* between 2014 and 2023. Results are shown for each model based on the posterior distribution of the difference between 2023 and 2014. Positive values represent areas with an increase in log-intensity over this period, while negative values indicate a decrease. For each pixel, the posterior mean, the 2.5th and 97.5th percentiles, the 95% coverage probability interval, and the significance are reported. In a Bayesian context, significance is defined as those posterior distributions of log-intensity differences whose 95% coverage probability interval does not include zero. Accordingly, a value of -1 indicates a 95% probability of a decrease in intensity, a value of 1 indicates a 95% probability of an increase, and a value of 0 indicates that the 95% credible interval likely includes zero, suggesting no significant change over time in that area.

**
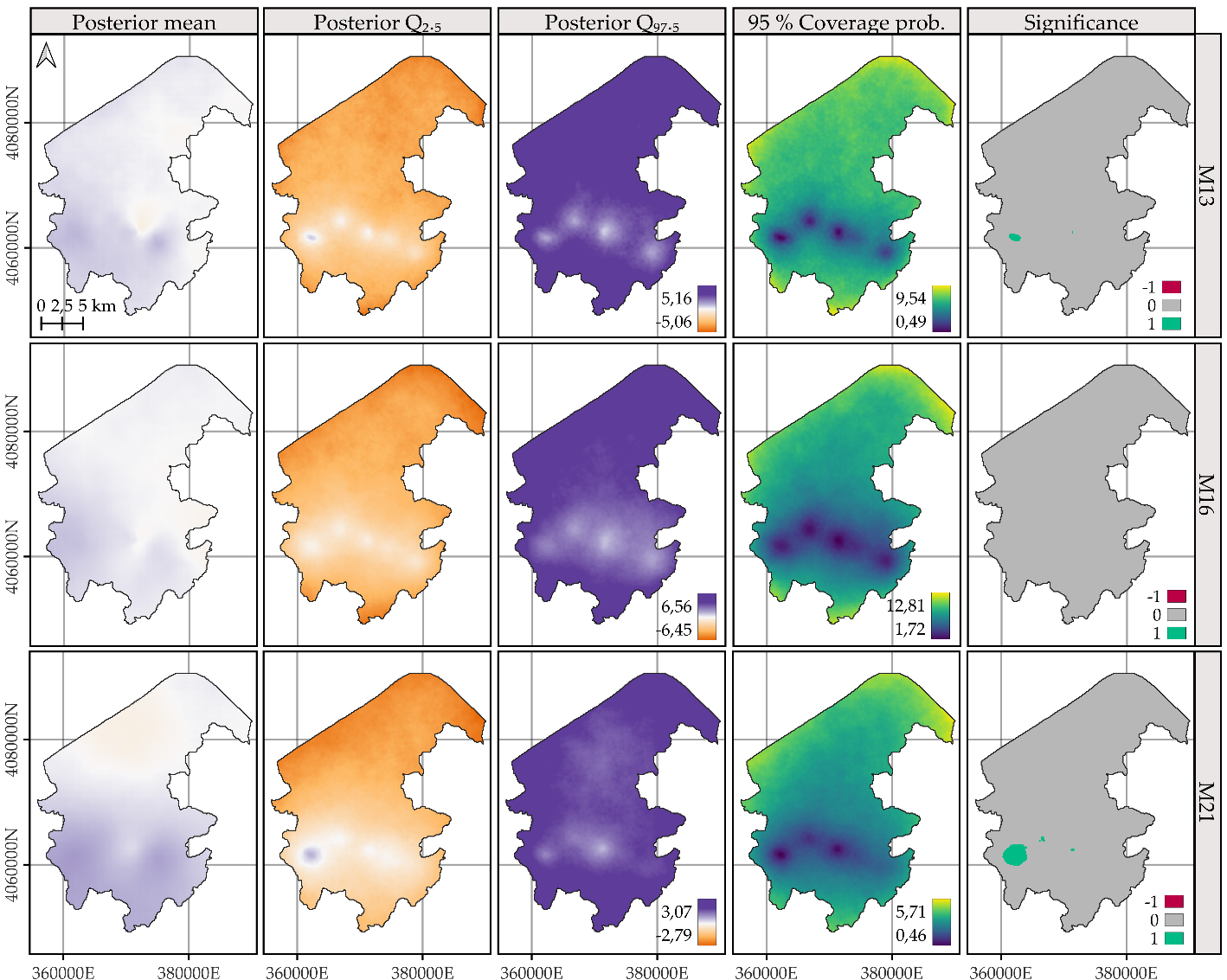
**

**Supplementary Figure 15.** Comparison of the net change in the log-intensity for *Pteris incompleta* between 2014 and 2023. Results are shown for each model based on the posterior distribution of the difference between 2023 and 2014. Positive values represent areas with an increase in log-intensity over this period, while negative values indicate a decrease. For each pixel, the posterior mean, the 2.5th and 97.5th percentiles, the 95% coverage probability interval, and the significance are reported. In a Bayesian context, significance is defined as those posterior distributions of log-intensity differences whose 95% coverage probability interval does not include zero. Accordingly, a value of -1 indicates a 95% probability of a decrease in intensity, a value of 1 indicates a 95% probability of an increase, and a value of 0 indicates that the 95% credible interval likely includes zero, suggesting no significant change over time in that area.

**
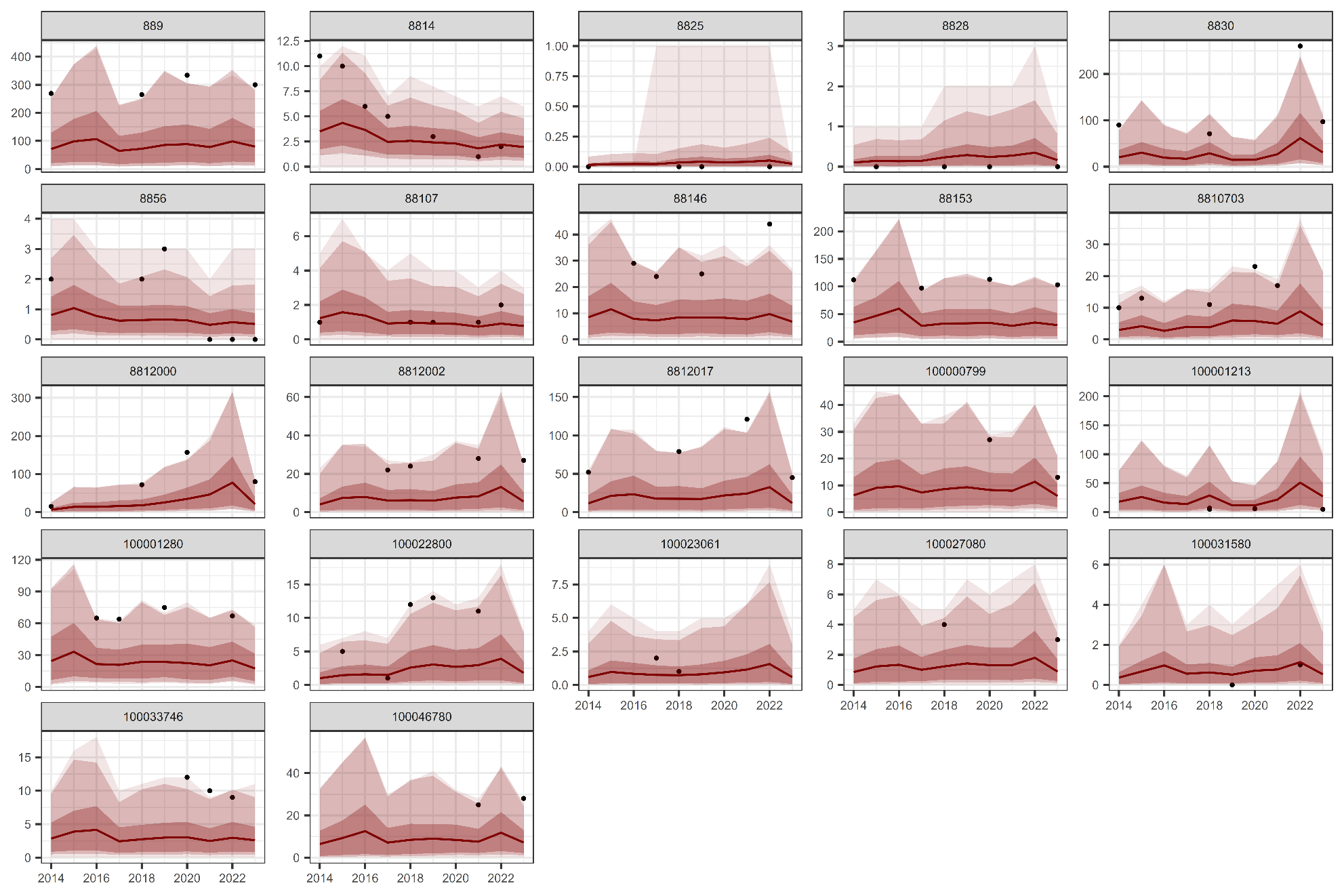
**

**Supplementary Figure 16.** Temporal interpolation of abundance for *Culcita macrocarpa* populations defined by the Fern Recovery Plan of Andalusia, based on model M13. Posterior distributions of the intensity and the predictive distribution per grid cell were aggregated according to their membership in each defined population, leveraging the Bayesian framework’s capacity to propagate uncertainty through posterior summaries. The figure shows observed count data alongside the posterior mean, the 75% credible interval, the 95% credible interval of the intensity distribution, and the 95% credible interval of the posterior predictive distribution.


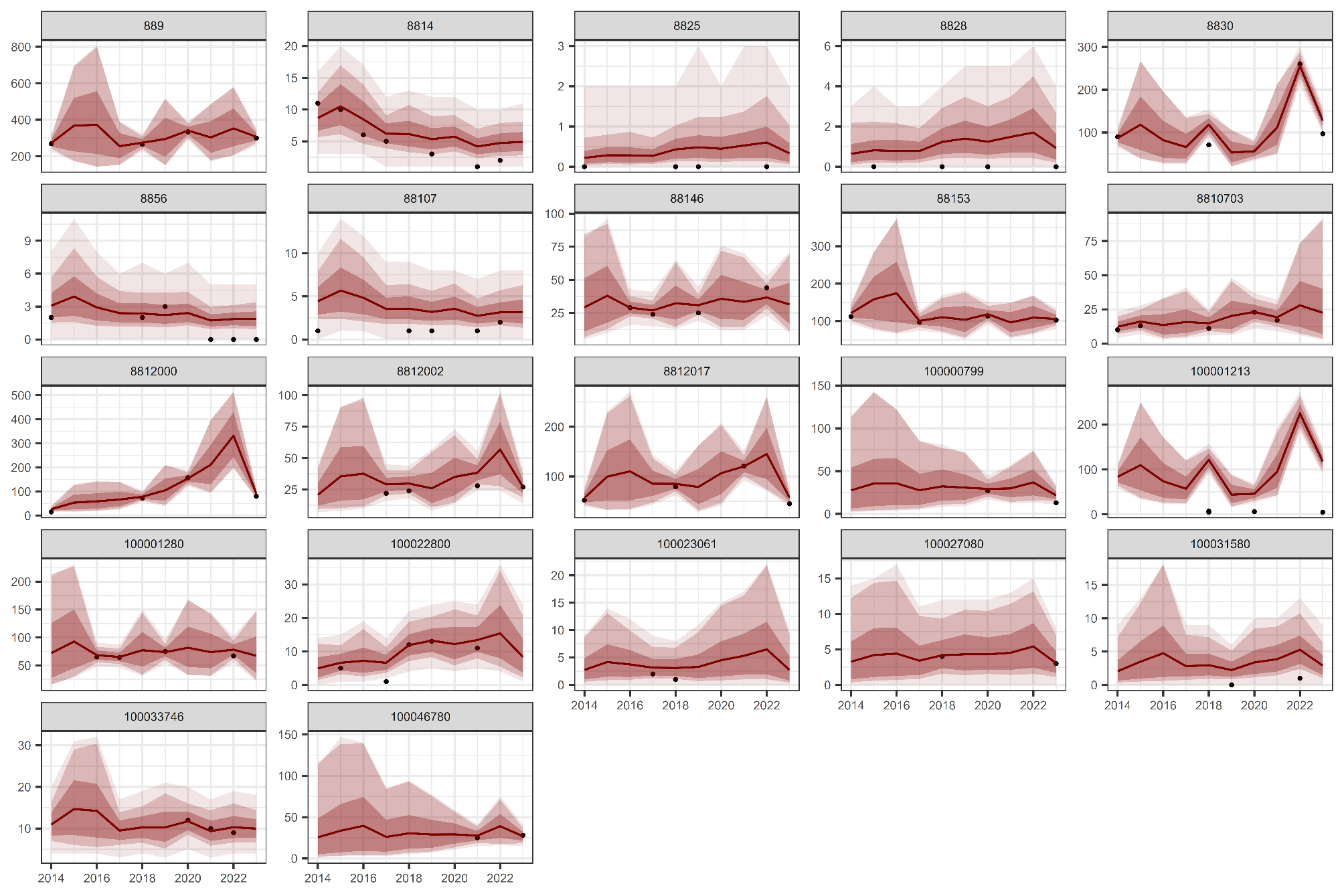


**Supplementary Figure 17.** Temporal interpolation of abundance for *Culcita macrocarpa* populations defined by the Fern Recovery Plan of Andalusia, based on model M21. Posterior distributions of the intensity and the predictive distribution per grid cell were aggregated according to their membership in each defined population, leveraging the Bayesian framework’s capacity to propagate uncertainty through posterior summaries. The figure shows observed count data alongside the posterior mean, the 75% credible interval, the 95% credible interval of the intensity distribution, and the 95% credible interval of the posterior predictive distribution.

**
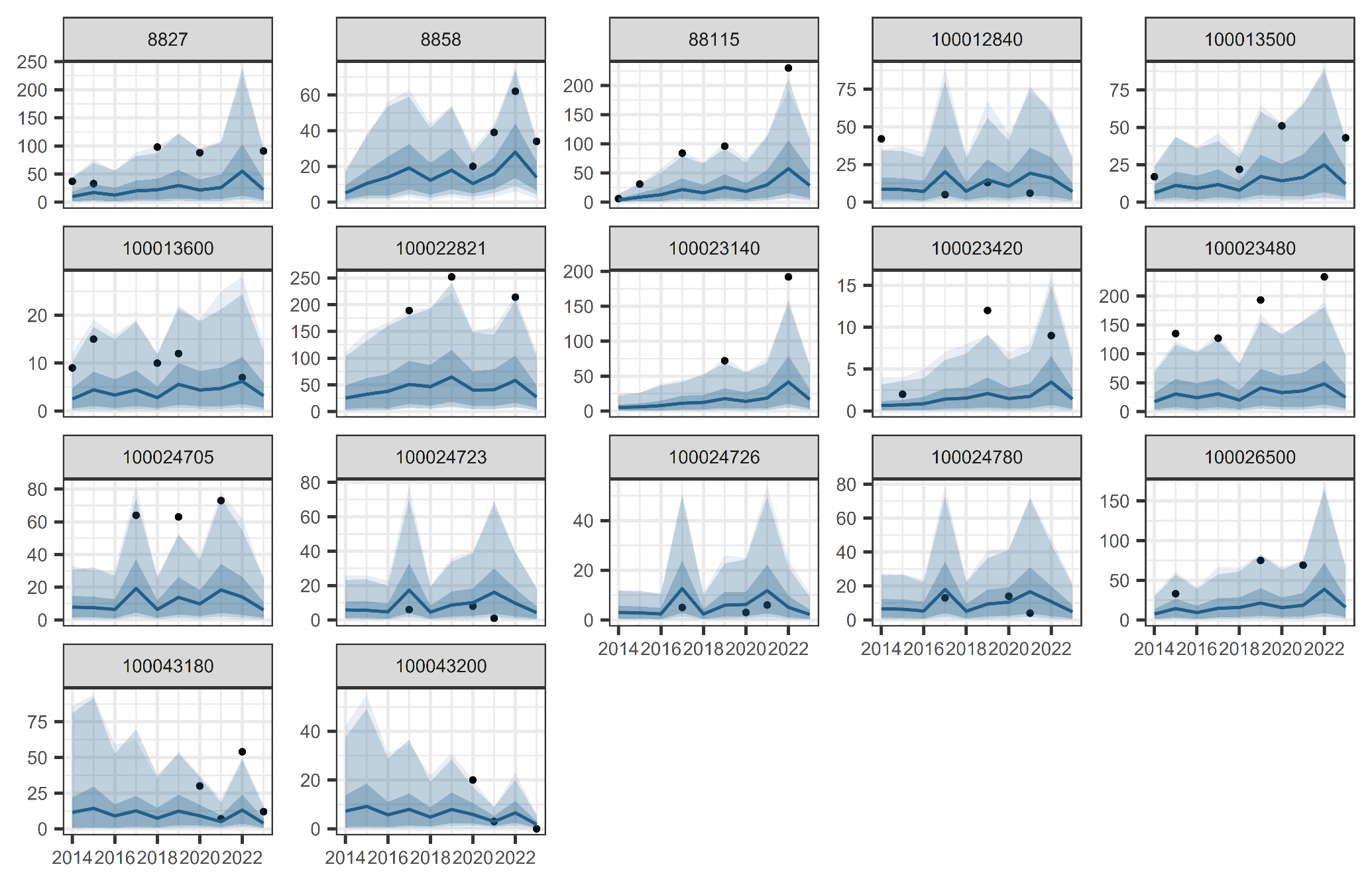
**

**Supplementary Figure 18.** Temporal interpolation of abundance for *Diplazium caudatum* populations defined by the Fern Recovery Plan of Andalusia, based on model M13. Posterior distributions of the intensity and the predictive distribution per grid cell were aggregated according to their membership in each defined population, leveraging the Bayesian framework’s capacity to propagate uncertainty through posterior summaries. The figure shows observed count data alongside the posterior mean, the 75% credible interval, the 95% credible interval of the intensity distribution, and the 95% credible interval of the posterior predictive distribution.


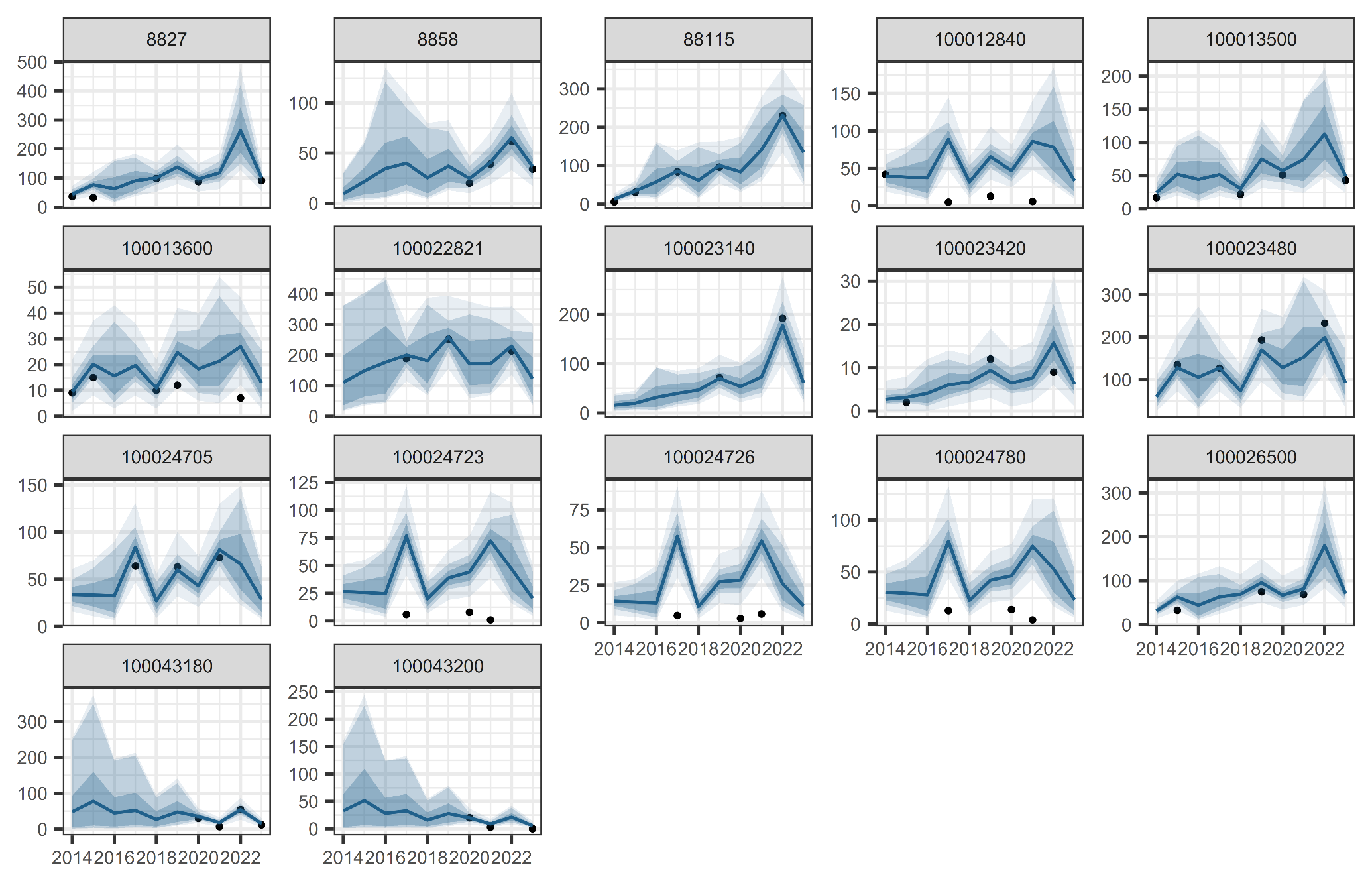


**Supplementary Figure 19.** Temporal interpolation of abundance for *Diplazium caudatum* populations defined by the Fern Recovery Plan of Andalusia, based on model M21. Posterior distributions of the intensity and the predictive distribution per grid cell were aggregated according to their membership in each defined population, leveraging the Bayesian framework’s capacity to propagate uncertainty through posterior summaries. The figure shows observed count data alongside the posterior mean, the 75% credible interval, the 95% credible interval of the intensity distribution, and the 95% credible interval of the posterior predictive distribution.


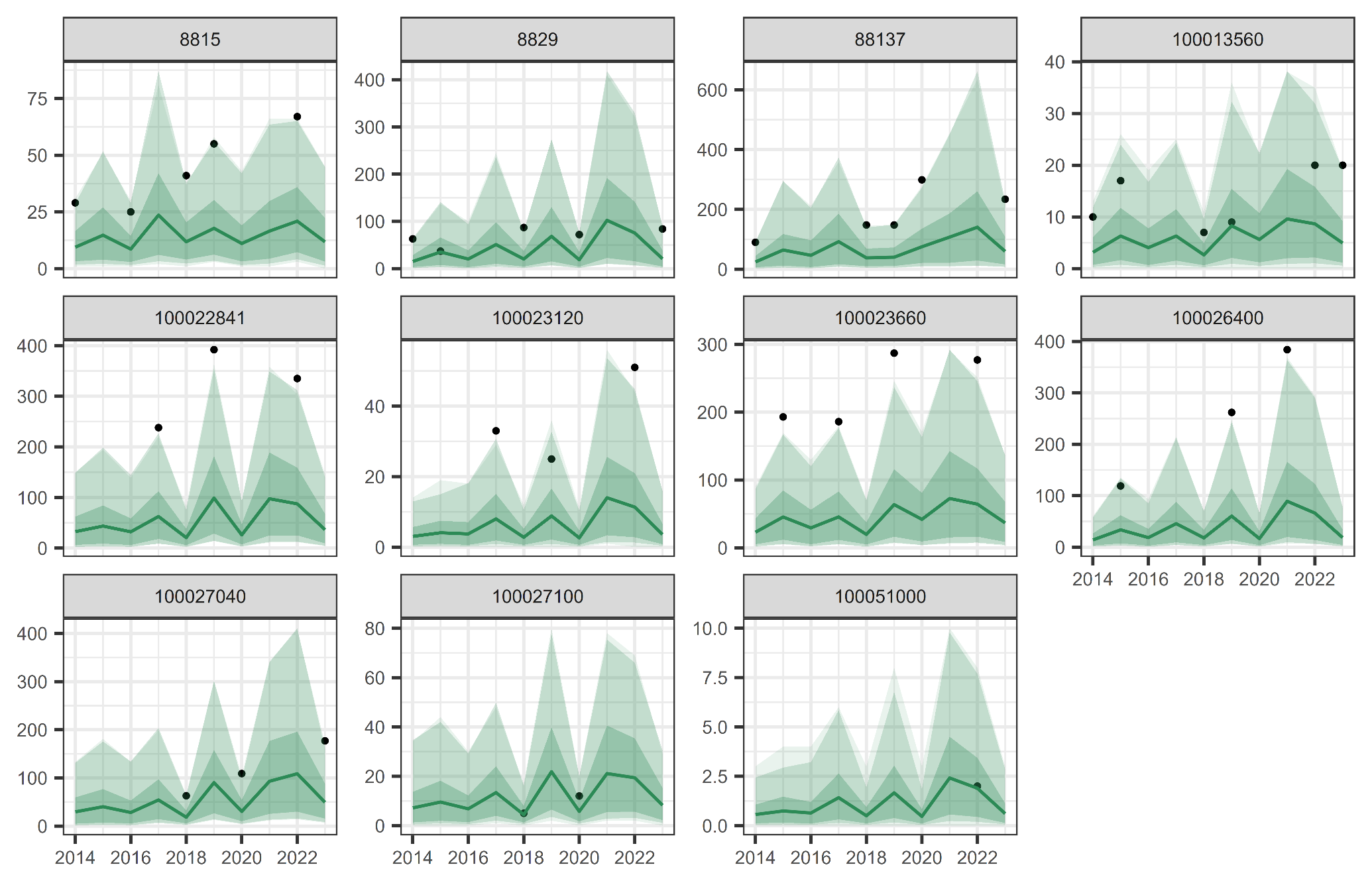


**Supplementary Figure 20.** Temporal interpolation of abundance for *Pteris incompleta* populations defined by the Fern Recovery Plan of Andalusia, based on model M13. Posterior distributions of the intensity and the predictive distribution per grid cell were aggregated according to their membership in each defined population, leveraging the Bayesian framework’s capacity to propagate uncertainty through posterior summaries. The figure shows observed count data alongside the posterior mean, the 75% credible interval, the 95% credible interval of the intensity distribution, and the 95% credible interval of the posterior predictive distribution.


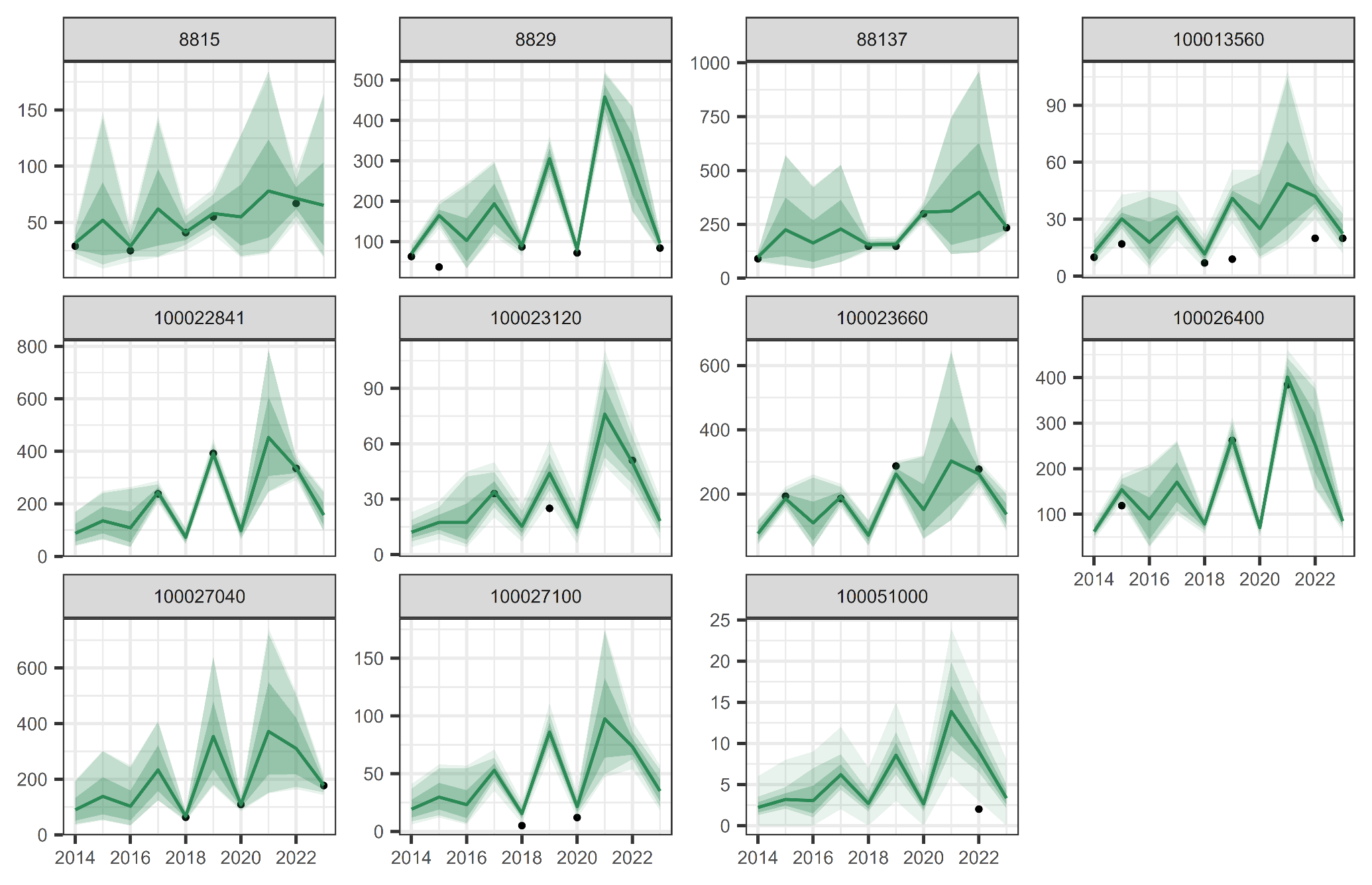


**Supplementary Figure 21.** Temporal interpolation of abundance for *Pteris incompleta* populations defined by the Fern Recovery Plan of Andalusia, based on model M21. Posterior distributions of the intensity and the predictive distribution per grid cell were aggregated according to their membership in each defined population, leveraging the Bayesian framework’s capacity to propagate uncertainty through posterior summaries. The figure shows observed count data alongside the posterior mean, the 75% credible interval, the 95% credible interval of the intensity distribution, and the 95% credible interval of the posterior predictive distribution.

| **Table S2.** Comparison of temporal interpolation capacity by species for models M13 and M21 for population abundance. pPCPI-I refers to the percentage of observations covered by the X% posterior coverage probability interval of the intensity. pPCPI-PPD denotes the percentage of observations covered by the X% posterior predictive distribution coverage probability interval. Nas refers to missing data within the temporal time series. MAE is the Mean Absolute Error calculated between observed count data and the posterior mean of the intensity. | | | | | | |
| --- | --- | --- | --- | --- | --- | --- |
|  | *Culcita macrocarpa* | | *Diplazium caudatum* | | *Pteris incompleta* | |
|  | M13 | M21 | M13 | M21 | M13 | M21 |
| 75% pPCPI-I | 12,94 | 51,76 | 24,59 | 47,54 | 10,00 | 70,00 |
| 95% pPCPI-I | 54,12 | 64,71 | 65,57 | 62,30 | 47,50 | 75,00 |
| 95% pPCPI-PPD | 76,47 | 91,76 | 81,97 | 75,41 | 45,00 | 80,00 |
| Mean 75% PCPI-I amplitude | 19,33 | 11,51 | 27,26 | 21,43 | 48,24 | 21,83 |
| Mean 95% PCPI-I amplitude | 43,60 | 19,83 | 63,00 | 36,55 | 111,14 | 37,29 |
| Mean 95% PCPI-PPD amplitude | 45,51 | 29,28 | 66,58 | 76,49 | 113,22 | 54,21 |
| Sd. 75% PCPI-I amplitude | 28,57 | 10,81 | 22,94 | 14,01 | 42,61 | 12,31 |
| Sd. 95% PCPI-I amplitude | 63,85 | 18,29 | 52,61 | 23,45 | 98,63 | 20,81 |
| Sd. 95% PCPI-PPD amplitude | 64,60 | 25,38 | 52,61 | 54,89 | 99,40 | 27,88 |
| Mean 75% PCPI-I amplitude for NAs | 22,00 | 42,69 | 21,42 | 48,11 | 51,26 | 82,91 |
| Mean 95% PCPI-I amplitude for NAs | 53,27 | 79,49 | 51,28 | 92,15 | 123,52 | 150,47 |
| Mean 95% PCPI-PPD amplitude for NAs | 54,16 | 82,91 | 54,34 | 109,31 | 124,88 | 158,16 |
| Sd. 75% PCPI-I amplitude for NAs | 33,32 | 56,69 | 17,91 | 44,25 | 53,98 | 96,99 |
| Sd. 95% PCPI-I amplitude for NAs | 78,77 | 103,58 | 42,76 | 86,61 | 133,24 | 178,71 |
| Sd. 95% PCPI-PPD amplitude for NAs | 77,64 | 102,98 | 43,45 | 92,01 | 131,28 | 177,00 |
| MAE | 30,95 | 8,39 | 41,39 | 18,32 | 86,82 | 11,46 |


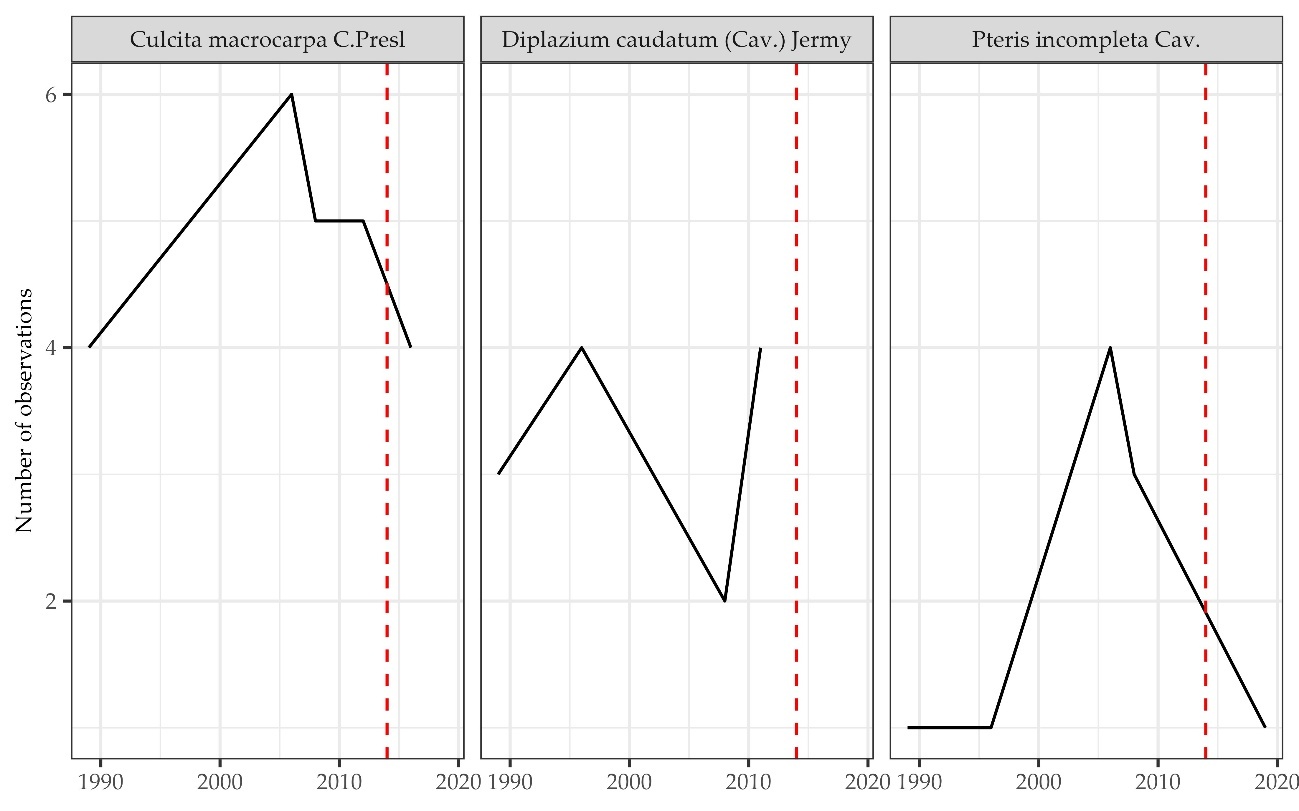


**Supplementary Figure 22.** Temporal pattern of the number of GBIF observations for the three study species. The red dashed line indicates the year 2014, when the first data from the Andalusian Fern Recovery Plan were collected, marking the point at which data integration becomes possible.


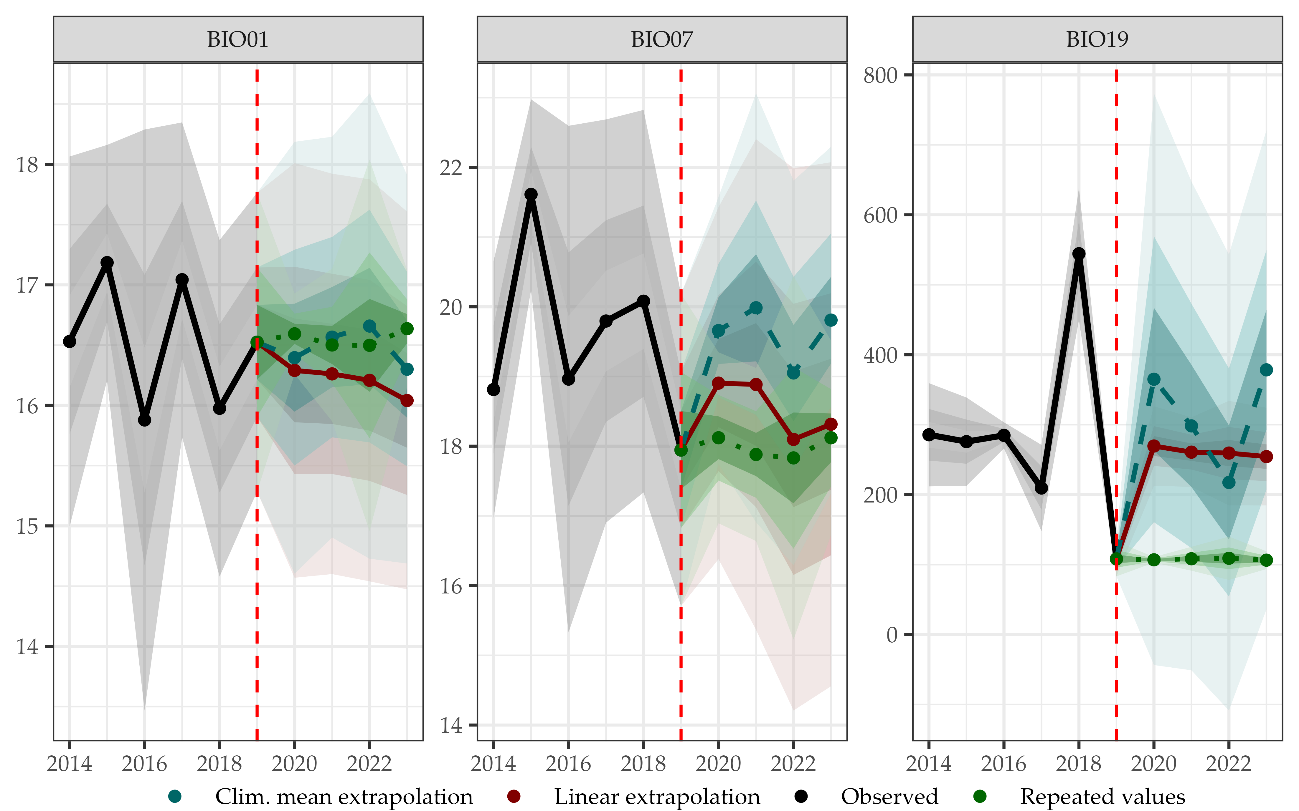


**Supplementary Figure 23.** Comparison of methods for generating annual extrapolations of bioclimatic time series. Points represent the spatial mean of extrapolated values across all grid cells within the study area for each year. Shaded intervals indicate spatial variability (±1, ±2, and ±4 standard deviations across grid cells, from lowest to highest transparency).Specifically, for each year in the extrapolation period, we calculated the linear trend-based projection for every pixel in the climate variable raster covering the study area. The plotted points show the mean value across all grid cells for each year, and the shaded intervals represent spatial uncertainty defined. The apparent variability in the extrapolation points thus reflects spatial heterogeneity across the study area


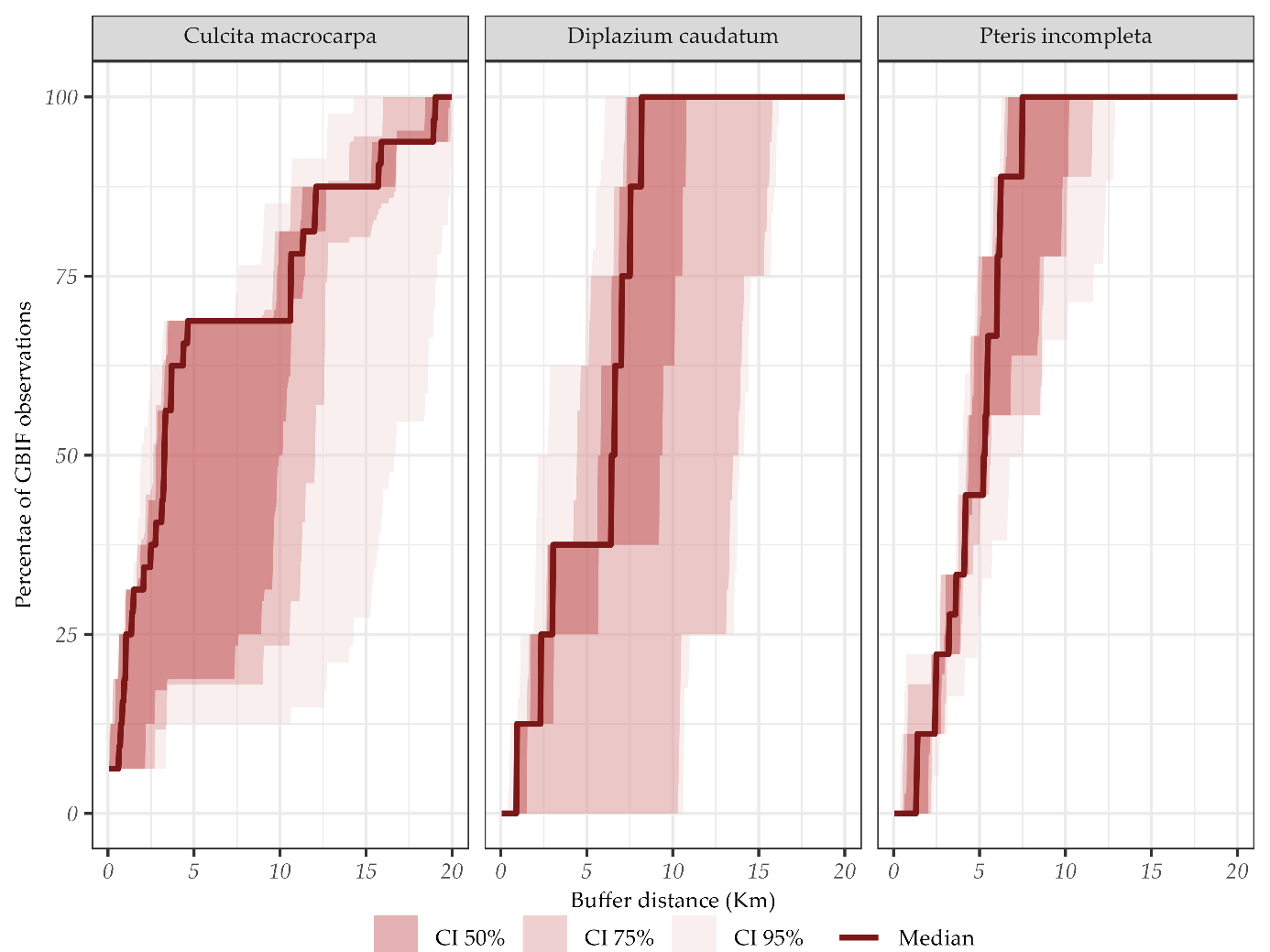


**Supplementary Figure 24.** Statistical distribution of the percentage of GBIF occurrence records within 100-m buffers around each spatial location of the structured abundance data from the Andalusian Fern Recovery Plan.
